# Investigating the role of baseline gut *Akkermansia muciniphila* and its co-metabolite palmitoleic acid in BCG vaccine efficacy: a preclinical study

**DOI:** 10.1016/j.ebiom.2026.106404

**Published:** 2026-07-23

**Authors:** Dongni Chen, Lingming Chen, Chun Chen, Yongen Yan, Xiaoyu Wu, Dongli Chen, Peibo Yuan, Xiaoxuan Long, Yi Zou, Jie Lin, Jun-Fa Xu, Jiang Pi, Gucheng Zeng, Yongjun Lu, Zhenhuang Ge

**Affiliations:** a Run Ze Laboratory for Gastrointestinal Microbiome Study, School of Life Sciences, Sun Yat-sen University, Guangzhou, 510275, China; b Institute of Laboratory Medicine, School of Medical Technology, Guangdong Medical University, Dongguan, 523808, China; c Guangdong Provincial Key Laboratory of Medical Molecular Diagnostics, The First Dongguan Affiliated Hospital, Guangdong Medical University, Dongguan, 523808, China; d School of Medicine, Shanghai General Hospital, Shanghai Jiao Tong University, Shanghai, 201620, China; e Microbiome Medicine Center, Department of Laboratory Medicine, Zhujiang Hospital, Southern Medical University, Guangzhou, 510280, China; f Dongguan Key Laboratory of Pathogenesis and Experimental Diagnosis of Infectious Diseases, The First Dongguan Affiliated Hospital, Guangdong Medical University, Dongguan, 523710, China; g Department of Microbiology, Zhongshan School of Medicine, Key Laboratory for Tropical Diseases Control of the Ministry of Education, Sun Yat-sen University, Guangzhou, 510080, China; h Guangdong Provincial Key Laboratory of Diabetology, Guangzhou Key Laboratory of Mechanistic and Translational Obesity Research, The Third Affiliated Hospital of Sun Yat-sen University, Guangzhou, 510630, China

**Keywords:** Gut microbiota, *Akkermansia muciniphila*, BCG, Palmitoleic acid

## Abstract

**Background:**

Bacillus Calmette–Guérin (BCG) is the only vaccine for tuberculosis (TB) and remains the most effective means of prevention; however, its effectiveness in humans is highly variable with unknown mechanism.

**Methods:**

Using microbiota transplantation and multi-omics strategies (16S rRNA sequencing, metabolomics, proteomics) in mouse models, we investigated the effect of pre-vaccination gut microbiota composition on BCG vaccine efficacy. Furthermore, key findings were validated in human datasets.

**Findings:**

Baseline abundance of gut microbiota, especially *Akkermansia muciniphila*, significantly affects BCG vaccine efficacy. Increasing the baseline levels of *A. muciniphila* in the gut before vaccination reduced the BCG vaccine responses, resulting in impaired protection against *Mycobacterium tuberculosis* (Mtb) infection in mice. *A. muciniphila*-contributed gut co-metabolite palmitoleic acid inhibits the vaccine responses of BCG. Palmitoleic acid acts on vaccine response through the mediation of effector protein MptpB of BCG and identified that MptpB-mediated inhibition of actin cytoskeleton remodelling to activate BCG vaccine responses is required for palmitoleic acid to regulate the efficacy of BCG vaccination. Inhibition of MptpB or actin cytoskeleton remodelling blocks the effects of higher baseline abundance of *A. muciniphila* on the efficacy of BCG vaccination. Analyses of human datasets provided hypothesis-generating support for these findings. Mechanistically, the higher baseline levels of gut *A. muciniphila* and its metabolite palmitoleic acid inhibit BCG vaccine efficacy by suppressing MptpB-mediated actin cytoskeleton remodelling.

**Interpretation:**

These results show that pre-vaccination differences in gut microbiota composition are a factor accounting for high variation in vaccine effectiveness. Stratifying by baseline gut microbiota profile and metabolism may represent a strategy to enhance future vaccine efficacy.

**Funding:**

Guang Dong Cheung Kong Philanthropy Foundation, Guangzhou key R & D project, National Natural Science Foundation of China, Key laboratory start-up project (Sixth Affiliated Hospital of Sun Yat-sen University), Guangdong Basic and Applied Basic Research Foundation, Guangzhou Basic and Applied Basic Research Foundation, Doctoral Initial Funding of Guangdong Medical University, Shenzhen Medical Research Fund, Discipline construction project of Guangdong Medical University, Project of Songshan Lake Innovation Center of Medicine & Engineering of Guangdong Medical University.

**Research in context**

**Evidence before this study**

TB remains a major global health burden, and the BCG vaccine is the only licenced vaccine available. However, its protective efficacy varies widely (0–80%) across different populations and geographic regions. According to previous studies, the gut microbiota may influence vaccine responses, including BCG. Human observational studies have reported associations between specific bacterial taxa and cytokine responses after BCG vaccination. Animal studies have shown that antibiotic-induced dysbiosis can alter vaccine immunogenicity. Nevertheless, causal evidence linking pre-vaccination gut microbiota composition to BCG efficacy, and the underlying molecular mechanisms, remained poorly understood. PubMed and Google Scholar were searched for articles from 1990 to 2025 using the terms “BCG vaccine”, “gut microbiota”, “*Akkermansia muciniphila*”, “vaccine efficacy”, and “tuberculosis”. No study to date has provided a mechanistic link between a specific gut microbe, its metabolite, and a BCG-encoded effector protein that regulates vaccine-induced protection.

**Added value of this study**

This study gives causal evidence: baseline abundance of gut *A. muciniphila* before vaccination directly affects BCG vaccine efficacy. Using faecal microbiota transplantation, oral gavage of *A. muciniphila*, and multi-omics approaches (16S rRNA sequencing, metabolomics, proteomics), we identified palmitoleic acid as an *A. muciniphila*-derived metabolite that inhibits BCG responses. Mechanistically, palmitoleic acid suppresses the phosphatase activity of the BCG-secreted effector protein MptpB, thereby blocking MptpB-mediated actin cytoskeleton remodelling, which are required for optimal vaccine-induced immunity. These findings were validated using a BCG strain lacking *mptpB* (BCGΔ*mptpB*) and by pharmacological modulation of actin polymerisation *in vivo*. Analyses of human datasets provided hypothesis-generating support for these findings.

**Implications of all the available evidence**

The results demonstrate that interindividual variation in gut microbiota composition, specifically the abundance of *A. muciniphila*, is a causal factor contributing to the variable efficacy of BCG vaccination. Screening for baseline gut *A. muciniphila* levels or its metabolite palmitoleic acid could help predict individual vaccine responses. Furthermore, modulating the gut microbiota before vaccination, for instance by reducing *A. muciniphila* abundance or inhibiting palmitoleic acid production, may represent a novel strategy to enhance BCG immunogenicity. These findings also highlight the broader principle that pre-vaccination gut microbial and metabolic profiles should be considered in the design of future vaccination programmes, including personalised vaccine strategies.

## Introduction

TB, caused by Mtb, remains a leading global health burden, with roughly 10 million new cases and 1.4 million deaths per year.^1^ The only available vaccine against TB is the BCG, a live-attenuated strain administered to over four billion individuals globally.^2^ Despite its widespread use, the effectiveness of BCG in preventing TB is suboptimal and varies significantly across countries, regions, and populations, with protection rates ranging from 0% to 80%.^3^ This variability has led to ongoing debates, a pressing need for improved vaccines and a deeper understanding of the factors influencing BCG performance.

Although numerous factors can affect vaccine efficacy, a growing body of findings from human clinical trials and animal experiments indicates that the gut microbiota contributes to regulating immune responses elicited by vaccination.^4^^,^^5^ In human cohorts, improved vaccine outcomes have been linked to particular bacterial groups. These links, however, depend on the vaccine regimen employed. Manipulating the intestinal microbiota, using interventions such as antibiotics, probiotics, genetically modified bacteria, etc., is considered a strategy to boost vaccine efficacy.^5^ Notably, factors that affect the microbiome are similar to those that impact vaccine immune responses, further underlining this mutual relationship between vaccine response and the gut microbiota.^6^

The gut microbiota is widely accepted both for its role in preserving general health and for its involvement in the gut–lung axis.^7^, ^8^, ^9^ The gut microbiome modulates immune responses through various mechanisms, including metabolite production and the regulation of immune cell differentiation and function.^9^, ^10^, ^11^ Gut dysbiosis has been shown to lead to a disrupted vaccine immune response in mice.^12^ Recent work has underscored the profound influence that gut microbiota exerts upon BCG's efficacy.^13^, ^14^, ^15^ Recent findings suggest gut microbiota along with their metabolites may affect lung immunity and inflammation, making them potential targets to mediate the efficacy of BCG.^16^ It may be possible to optimise the immune response to BCG, by modulating the gut microbiome, to improve its protective effects against TB. These studies implies that the gut microbiota could serve as one element affecting how BCG exerts its protection.

Although links between the microbiota and vaccine efficacy are increasingly documented, its causality evidence and mechanistic insights remain poorly understood. In this study, we used pre-treated animal model alongside multi-omics approach to investigate how the gut microbiota affects BCG-induced vaccine efficacy. We identified specific immunomodulatory bacterial species and elucidated the mechanisms by which bacteria-derived metabolites influence vaccine response by inhibiting the activity of key effector proteins essential for BCG efficacy. These findings were corroborated by several human datasets. Our findings offer a promising strategy for developing complementary interventions to enhance BCG vaccination and ultimately contribute to global efforts to control and eliminate TB.

## Methods

### Bacterial strains and cell culture

The BCG (Pasteur), H37Rv (ATCC 27294) and H37Ra (ATCC 25177) strains were cultured at 37 °C in liquid 7H9 (Middlebrook, #271310) supplemented with OADC (Middlebrook, #212351), 0.2% glycerol (Aladdin, #G116203), and 0.05% Tween 80 (Aladdin, #T104865). Bacterial viability was assessed by plating cells on 7H11 (Middlebrook, #212203) agar containing 0.5% glycerol and 10% OADC. Anaerobic cultivation of *A. muciniphila* (ATCC BAA-835) took place at 37 °C using BHI medium (HKM, #024053). *Escherichia coli* strains (DH5α, BL21 (DE3) and HB101) were grown in LB medium for plasmid transformation, protein expression and genetic manipulation, respectively. Culture of J774A.1 cells (RRID:CVCL_0358), Raw 264.7 (RRID:CVCL_0493), and HEK293T (RRID:CVCL_0063) was performed in DMEM containing 10% foetal bovine serum (FBS) (Gibco, #10099-141), with humidified 5% CO_2_ at 37 °C. The cell lines were obtained from a reputable source and is widely used in immunology and infectious disease research. We routinely monitor cell morphology, growth characteristics, and viability, and the cells are tested periodically for mycoplasma contamination. The relevant documentation is provided in the Supplementary Data.

The BCG strain with deletion of *mptpB* (BCGΔ*mptpB*) was generated by phage specialised transduction as described previously.^17^ PCR and sequencing verified the deletion. The BCGΔ*mptpB* strain was complemented by introducing wild-type MptpB carried on the mycobacterial shuttle vector pMV261, resulting in BCGΔ*mptpB*/C. Growth of all strains took place at 37 °C. When necessary, antibiotics were supplied at the following final concentrations: kanamycin (50 μg/mL applied to *E. coli* and 25 μg/mL to mycobacteria), together with hygromycin (150 μg/mL applied to *E. coli* and 75 μg/mL to BCG).

### Faecal microbiota transplantation

In this single experiment, a total of 36 mice were used. Microbiota donor mice consisted of 6 vaccination-naïve and 6 vaccination-adapted donors (12 donors in total), and faeces from each donor group were pooled prior to transplantation to minimise individual variation. For microbiota recipient mice, 6 recipients of C-GM and 6 recipients of B-GM were used to assess BCG vaccine responses (harvested 4 weeks after vaccination). Another 6 recipients of C-GM and 6 recipients of B-GM were used to evaluate vaccine protection; these mice were challenged with Mtb 4 weeks after BCG vaccination and then assessed for lung injury after 5 weeks of infection. Faecal microbiota transplantation was performed as we previously described,^11^ mice before vaccination were pre-treated with antibiotic solution composed of four antibiotics: ampicillin, vancomycin, neomycin and metronidazole were obtained from Sigma at the following concentrations and CAS numbers respectively: 1 g/L (7177–48-2), 0.5 g/L (1404–90-6), 1 g/L (1405–10-3), and 1 g/L (443–48-1). Antibiotic-pretreated mice were gavaged with faecal samples collected from either saline-treated (vaccination-naïve) mice or from mice that had been subcutaneously vaccinated with BCG (5 × 10^5^ CFU) and housed for 4 weeks after vaccination (“vaccination-adapted” donors). After the 4-week interval, faecal specimens were obtained from these donor mice. For faecal transplantation, specimens were suspended in sterile saline at 2 mg/mL. This mixture then stood for 2 min at room temperature. Recipient mice, after one week of broad-spectrum antibiotic pretreatment, were given 200 μL of the faecal supernatant via oral gavage twice per week over a 2-week period.

### Subcutaneous immunisation with BCG

To prepare BCG suspension, 1 mL of the mycobacterial culture in the logarithmic growth phase was harvested. The sample was then spun at 12,000 × *g* for 30 s, followed by two washes with 1 mL of PBST (PBS (Gibco, #C10010500BT) supplemented with 0.05% Tween-80). After gentle resuspension of the pellet using 1 mL PBST, a 2-min centrifugation at 500 × *g* was applied. Prior to subsequent experiments, the collected supernatant was normalised by absorbance at 600 nm (OD600). Following a 21-day incubation at 37 °C, colony-forming units (CFUs) were enumerated on the plates. Mice subcutaneously vaccinated with BCG were immunised subcutaneously with 5 × 10^5^ CFU of the BCG Pasteur strain. For assessing protection conferred by BCG immunisation, mice were challenged with Mtb via aerosol at 4 weeks post-vaccination. Mtb H37Rv was cultured in 7H9 liquid medium to the logarithmic growth phase. The bacterial culture was collected, dispersed by sonication, and filtered to obtain a single-cell suspension. Aerosol infection was performed using a commercial aerosol inhalation device (Glas-Col, USA). Briefly, mice were placed in the aerosol chamber, and the bacterial suspension was nebulised using a nebuliser. Mice inhaled the aerosol for 30 min. The initial infectious dose was consistently controlled at 150 CFU per lung for each experiment. Control mice were subcutaneously administered saline used for the preparation of BCG. For evaluating how gut microbiota affects vaccine efficacy, mice were first colonised with faecal microbiota from BCG- or saline-treated mice for 2 weeks, followed by BCG vaccination. To assess the influence of *A. muciniphila* on vaccine efficacy, mice were first colonised with *A. muciniphila* three times per week for 2 weeks, followed by BCG or BCGΔ*mptpB* vaccination. To assess the influence of palmitoleic acid on vaccine efficacy, mice were first given drinking water containing palmitoleic acid for 2 weeks, followed by BCG or BCGΔ*mptpB* vaccination. Detailed information on the treatment with *A. muciniphila* and palmitoleic acid was obtained as we previously described.^9^ To assess the influence of actin cytoskeleton remodelling on vaccine efficacy, mice were injected with jasplakinolide (JasP, an actin polymerisation inducer) and cytochalasin D (CytoD, an actin polymerisation inhibitor) when vaccinated with BCG or BCGΔ*mptpB*.

### Measurement of vaccine-induced immunity

Serum IgG concentrations were measured using an ELISA immunosorbent assay. The purified BCG vaccine protein derivative (Chengdu Institute of Bioproducts, China) was coated on microtiter plates at 100 μL per well (20 μg/mL) and then left to incubate overnight at 4 °C. After washing the plates using PBS containing 0.5% Tween-20, they were blocked with 1% bovine serum albumin. Serum samples from mice were diluted and incubated on blocked plates. HRP-conjugated anti-mouse IgG (Abmart, Cat #M21001L, RRID:AB_2713950) served as the detection reagent for antigen-specific antibodies at a dilution of 1:2000. TMB substrate was used to visualise HRP activity, after which absorbance readings were taken at 450 nm. Lung specimens were then minced and subjected to digestion using collagenase type I (Sigma–Aldrich, St. Louis, MO, USA) at 37 °C within a shaking incubator. A single-cell suspension was generated by filtering the digested tissue through a 40 μm cell strainer. The spleens were similarly digested with collagenase type IV at 37 °C in a shaking incubator. EDTA was applied to the digested material, which was then filtered through a 40 μm cell strainer to yield a splenocyte single-cell suspension. After red blood cell lysis, the pelleted cells were taken up in full RPMI 1640 medium prior to ex vivo culture. Mtb H37Rv lysates (10 μg/mL, Gene Optimal, Cat #GOMY0086) were either added to the cell culture medium or omitted and the supernatants were analysed for cytokine production. Lung TNF-α and spleen IFN-γ levels were quantified by commercial ELISA kits (TNF-α: Cloud-Clone Corp, SEA133Mu; IFN-γ: Cloud-Clone Corp, SEA049Mu) following the supplier's recommended procedures.

### Flow cytometric analysis

Experiments were conducted as we previously described.^9^ Briefly, single-cell suspensions were either left unstimulated or re-stimulated ex vivo with Mtb lysate in complete RPMI-1640 at 37 °C and 5% CO_2_ for a total of 24 h. Brefeldin A (5 μg/mL, BioLegend, #420601) and monensin (2 μM, BioLegend, #420701) were added during the final 6 h of incubation. After initial labelling of surface markers, cells were permeabilised and fixed for 30 min, then incubated with intracellular molecule antibodies for 45 min, followed by a final fixation in 2% formalin/PBS and preparation for polychromatic flow cytometry. For flow cytometric analysis, we employed the following antibody panel: anti-mouse CD4-FITC (GK1.5, BioLegend, RRID:AB_312691), CD8-BV510 (53-6.7, BioLegend, RRID:AB_2563057), TNF-α-APC (MP6-XT22, BioLegend, RRID:AB_315429), and IFN-γ-PE-Cy7 (XMG1.2, BioLegend, RRID:AB_2295770). Acquisition of data was performed on a BD LSRFortessa X-20, and data were analysed using FlowJo version 10.8.1. All antibodies employed here were purchased from commercial vendors (with respective manufacturers and catalogue numbers) and have been widely used in other studies. All antibodies were used according to the manufacturers’ recommendations. The relevant documentation is provided in the Supplementary Data.

### Mice infection and sample collection

Female C57BL/6J mice (3 weeks old) were randomly divided into experimental groups and maintained in a temperature-regulated SPF facility with ad libitum food and water under a 12-h light/dark cycle. Female mice were selected to minimise aggression-related stress confounding, ensure consistency with prior tuberculosis vaccine studies, and control for sex as a biological variable. Mice housed in the same room were used in each experiment. Before receiving the BCG or BCGΔ*mptpB* vaccine, mice were administered various pre-treatments, and then underwent a 4-week vaccination period. Subsequently, Mtb infection was induced by aerosol exposure. Infection doses of Mtb strains were prepared as previously described.^9^^,^^16^ Mice were infected with 150 CFU H37Rv or 5 × 10^5^ CFU H37Ra over a 5-week period within a Biosafety Level-3 facility. Faecal samples, mouse lungs, spleen, and serum were collected at either 4 weeks post-vaccination or 5 weeks post-infection. Faecal samples were prepared for microbiota composition analysis, metabolite profiling, and microbiota transplantation. Following collection, blood samples were left to clot for 30 min under ambient conditions, then spun at 3000 × *g* for 10 min. Both serum and faecal specimens were kept at −80 °C pending subsequent assays.

### Ethics

The animal study protocols received approval from the ethics committee of Zhongshan School of Medicine, Sun Yat-sen University (SYSU-IACUC-2023-B0435), and all procedures complied with the National Regulations for Animal Care and the ARRIVE guidelines.

### Bacterial and histopathological analysis of Mtb-infected mice

For evaluation of mycobacterial load, murine lung specimens were gently homogenised, and CFU enumeration was performed according to established protocols.^9^ To examine tissue histopathology, lung specimens underwent fixation with 10% zinc-formalin, followed by paraffin embedding. Thereafter, 5-μm-thick sections were prepared, stained with haematoxylin and eosin (H&E), and visualised on an AxioScan Z1 digital slide scanning system. Acid-fast stained images were captured using an Olympus BX51 microscope. Lung tissues received a histological score according to the degree of granulomatous change, graded as: 0 = no abnormality; 1 = minimal involvement (1–10% of tissue area); 2 = mild involvement (11–30%); 3 = moderate involvement (31–50%); 4 = marked involvement (51–80%); 5 = severe involvement (>80%). Mtb burden in the lungs was assessed by CFU counting and histopathological analysis, with histological scoring performed by investigators blinded to the experimental group allocation.

### Faecal 16S rRNA gene sequencing

Faecal specimen preparation and 16S rRNA gene sequencing followed our earlier published protocols.^9^^,^^10^ Faecal specimens were processed for bacterial DNA extraction with the QIAamp Fast DNA Stool Mini Kit (Qiagen, Cat# 51604) according to the supplier's instructions. Amplification by PCR targeted the V3–V4 hypervariable region of the 16S rRNA gene with barcoded primer pairs. Sequencing of the amplicons was performed on an Illumina NovaSeq 6000 system (Novogene Co. Ltd., China) using standard protocols. Sequencing data were analysed using QIIME (version 1.9.1). The sequence data were submitted to the Sequence Read Archive (SRA) repository. Visualisation of the resultant matrices was achieved via PCoA plots. For gut microbiota, cytokines and metabolites, heatmaps were produced employing the “heatmap” function in R. LEfSe analysis was used to determine significant changes in relative abundance through linear discriminant analysis (LDA). Correlations were evaluated employing Spearman's rank correlation coefficient. R Studio (version 3.5.0) was utilised to perform random forest analysis. For detecting *A. muciniphila*, the primer sequences are given in Table S1.

### Liquid culture, oral administration and detection of *A. muciniphila*

BHI medium served for the anaerobic culture of *A. muciniphila* at 37 °C, as we previously described.^9^^,^^10^ Bacterial growth was tracked at 12-h intervals through OD_600_ measurements taken with a Genesys spectrophotometer (Thermo Scientific). To prepare *A. muciniphila* for mouse gut colonisation, harvested cultures were then concentrated using an anaerobic saline solution supplemented with 25% glycerol. Antibiotic pre-treated mice were then orally administered 200 μL of either this bacterial suspension (with 2 × 10^8^ cells) or saline by oral gavage, on three occasions per week. To assess the colonisation of *A. muciniphila*, faecal samples were analysed for bacterial abundance by qPCR. Samples underwent genomic DNA extraction with the QIAamp DNA Tissue or Stools Mini Kit (Qiagen) according to the supplier's instructions. SYBR Green-based qPCR quantified *A. muciniphila* levels (primers listed in Table S1).

### Co-Culture of cells with live or pasteurised inactivated *A. muciniphila*

Gut *A. muciniphila* was incubated together with cells as we previously described.^9^^,^^10^ Briefly, Peripheral blood mononuclear cells (PBMCs) were separated from fresh blood anticoagulated with lithium heparin using Ficoll–Paque Plus density gradient centrifugation. The procedure involved layering Ficoll–Paque Plus beneath the blood sample, followed by centrifugation at 1000 × *g* for 20 min at 20 °C. After washing the recovered mononuclear cells with PBS (pH 7.4), they were finally taken up in RPMI medium containing 10% foetal bovine serum and maintained in culture for subsequent assays. For co-culture experiments, PBMC cells isolated from vaccinated mice were seeded in the bottom of 6-well plate (Thermo Scientific). PBMCs were first infected with BCG at a dose of 2 × 10^5^ CFU/well for 4 h at 37 °C. To eliminate extracellular bacteria, the cells were exposed to gentamicin at 50 μg/mL (Gibco, #GC19553) over a 1-h period, and then washed five times with PBS. Active or pasteurised *A. muciniphila* was then added to the upper chamber at MOI = 10^2^, and co-cultured at 37 °C for 72 h. After washing, cultured *A. muciniphila* were pelleted and re-suspended in anaerobic PBS containing 25% (v/v) glycerol. During the co-culture period, fresh active or pasteurised *A. muciniphila* was replenished every 24 h at the same MOI to maintain continuous stimulation. Pasteurisation is a well-established method for inactivating *A. muciniphila* metabolism while preserving bacterial cell components. Following the co-culture period, cellular specimens were harvested.

### Treatments of palmitoleic acid

Drinking water that contained sodium hydroxide was supplemented with palmitoleic acid (Sigma, #76169) to reach a final concentration of 36 mM as we previously reported.^9^ The control consisted of water containing an identical concentration of NaOH. Separately, 100 mg of palmitoleic acid was dissolved in 0.1 M aqueous NaOH. Inhibition of MptpB enzyme activity was induced by increasing the palmitoleic acid dose. A range of concentrations of palmitoleic acid was added to the BCG or macrophage co-cultures according to the physiological concentration in humans.

### Public dataset acquisition and analysis

Public data from population cohorts were used in this study. Data from a previous study^13^ investigating the impact of the gut microbiome in shaping immune memory responses within 321 healthy subjects following vaccination with BCG are available in the NCBI BioProject under the identifier PRJNA685797. Data from our previous research^9^ on gut microbiota dysbiosis and abnormal immune responses caused by TB infection in two independent cohorts have been deposited in the NCBI BioProject (accession: PRJNA609532). RNA-seq and ATAC-seq data from a previous study^18^ that examined how four widely used BCG strains (selected for their diverse usage and virulence, covering all four classes: I (BCG-Russia), II (BCG-Sweden), III (BCG-China), and IV (BCG-Pasteur)) differ in their capacity and underlying mechanisms for inducing trained immunity are accessible in the NCBI Sequence Read Archive under accession numbers PRJNA992960, PRJNA993298, and PRJNA993603. Unless otherwise indicated in the legend, we referred to the analysis methods and conditions used in their studies. The baseline characteristics of these datasets were detailed in Table S2.

### Actin cytoskeleton remodelling treatment

To determine the effect of actin polymerisation inhibition in mice pre-treated with *A. muciniphila*, the BCG vaccine treatments were concurrently injected with 3 μM Cytochalasin D (MCE, HY-N6682) or vehicle. To determine the effect of actin polymerisation inhibition in mice vaccinated with BCGΔ*mptpB*, the mice were concurrently injected with 3 μM Cytochalasin D or vehicle. Destabilisation of the actin cytoskeleton of cells was conducted using 1 μM Cytochalasin D. To determine the effect of actin polymerisation induction in mice vaccinated with BCG, mice were concurrently injected with 20 nM jasplakinolide (MCE, HY-P0027) or vehicle. A stock solution of the compound was prepared by dissolving it in a vehicle consisting of 10% DMSO (D2650, Sigma Aldrich), 40% PEG300 (HY-Y0873, MCE), 5% Tween 80 (HY-Y1891, MCE), and 45% saline.

### Infection of BCG in activated and resting macrophages

Based on its advantages in genetic stability and functional consistency with primary macrophages as a tool for mechanistic validation, J774A.1 macrophages, either left untreated or pre-stimulated for 16 h with recombinant IFN-γ (200 IU/mL, MCE, HY-P7025), were plated into six-well plates at 2 × 10^5^ cells per well. After washing with PBS, the culture medium was replaced with DMEM (Gibco, #C11995500BT) supplemented with 10% heat-inactivated FBS. Bacterial strains were washed twice using DMEM, then taken up in DMEM supplemented with FBS. Infection was conducted at an MOI of 1:10 using the both wild-type and mutant strains. Following infection, the cells were kept for 6 h under 5% CO_2_ at 37 °C. They were then rinsed with PBS and subsequently exposed to DMEM supplemented with 10% FBS, 1% antibiotic-antimycotic, and 20 μg/mL amikacin. At predetermined time points, lysis of the infected cells was carried out using 0.1% Triton X-100 over 15 min. Bacterial counts at various time intervals were determined by spreading serial dilutions onto 7H11 medium and incubating for 3 weeks at 37 °C.

### Infection of human monocyte-derived macrophages

Venous blood from healthy donors was subjected to density gradient centrifugation to isolate PBMCs. Subsequent purification of monocytes employed the plastic adherence method. In brief, per T25 flask, 10–15 × 10^6^ PBMCs were placed in 5 mL of complete culture medium (CTM), namely RPMI 1640 supplemented with 10% FBS, 4 mM l-glutamine, and 1% penicillin-streptomycin (Gibco, #15140122), and left to attach for 2 h at 37 °C under 5% CO_2_. Non-adherent cells were then discarded, and the adherent monolayer was gently washed twice with CTM to yield enriched monocytes. A suspension of 2 × 10^6^ monocytes was prepared in 500 μL of CTM and incubated in 24-well plates for 7 days. Recombinant human M-CSF (MCE, HY-P70488) was then added to the culture medium. Subsequently, macrophages derived from these monocytes were exposed to mycobacterial strains with an MOI of 10:1 over 2 h. After the cells received a 1-h gentamicin (50 μg/mL) treatment to eliminate extracellular bacteria, washed five times with PBS, and replenished with fresh culture medium. Cells were further incubated at 37 °C with 5% CO_2_, and colony-forming units (CFU) were counted as previously described.

### Immunoblot analysis

Immunoblotting was done using a method we earlier reported.^10^ Briefly, macrophage samples were lysed and the total protein was extracted and quantified. These proteins were subsequently resolved via electrophoresis using a 10% SDS-PAGE gel containing loading buffer. Following separation, they were electrotransferred onto PVDF membranes (Bio-Rad, #1620177) and then incubated for 1 h in 5% non-fat milk to block non-specific binding. Primary antibody incubation was carried out on the membranes overnight at 4 °C. Afterwards, secondary antibodies were applied for 1 h at room temperature. An ECL kit (Millipore, #WBKLS0500) served to visualise the protein bands, which were then imaged with a Tanon 5200 system (Tanon). Quantification of the data was performed using ImageJ (version 1.43).

### RNA extraction and real-time PCR

mRNA expression was determined as we previously reported.^10^ TRIzol reagent (Takara, Japan) was employed to extract total RNA from tissue or cell samples. The RNA concentration was then measured with a Nanodrop spectrometer. For first-strand cDNA synthesis, a Promega cDNA Synthesis Kit was used according to the supplier's instructions. qPCR was performed in triplicate using SYBR Green to assess TNF-α, IL-6, and IL-1β expression levels. Expression data were normalised to GADPH as an endogenous control and analysed using an ABI Step One Plus qPCR system (Applied Biosystems). The primers were provided in Supplementary Table S1.

### Metabolomics analysis

Frozen samples kept at −80 °C were defrosted over ice. To obtain metabolites, methanol (300 μL) was dispensed into each 100 μL specimen, mixed for 3 min, then allowed to stand at ambient temperature for 10 min. After centrifugation at 12,000 × *g* and 4 °C for 20 min, the supernatants were analysed using LC-MS. First- and second-order spectral data were qualitatively analysed by querying the self-database, the metware database, and public metabolite databases. Quantification was carried out using MRM triple quadrupole mass spectrometry. Unsupervised PCA was conducted using prcomp in R, with VIP ≥1, *P* < 0.01, and absolute FC ≥ 1.5, to screen for significantly altered metabolites. The adjusted *t*-test served to assess the statistical significance of each metabolite. The VIP values were calculated from the OPLS-DA data. Spearman correlation coefficients between samples were calculated with the cor function in R studio and displayed as heatmaps. The identified metabolites underwent annotation using the KEGG database and subsequently mapped to KEGG pathway. A hypergeometric test (based on P-value) served to determine the pathways containing significantly altered metabolites.

### Molecular docking

Molecular docking was performed using DeepMice, an AI-based online system for molecular docking and virtual screening, accessible through its web browser interface (http://www.deepmice.com/). Initially, the receptor file for docking was identified by retrieving the PDB ID 2OZ5 for MptpB from UniProt (https://www.uniprot.org/). Receptor information was uploaded using the default settings. Upon successful uploading of the PDB ID, the user interface displays information from the PDB file and initiates a protein repair. The docking pocket was selected based on the known enzymatic active site of MptpB. Subsequently, mol2-formatted small molecule files were uploaded to the online docking system. Molecular docking was then initiated and the docking results were visualised using PyMOL. Higher docking scores indicated a greater affinity.

### Expression and purification of MptpB proteins

MptpB from *M. tuberculosis* was expressed and purified, as we previously described.^19^ The point mutation MptpB(C160S) was generated using an overlap extension PCR. Briefly, plasmids for bacterial expression were generated by inserting the DNA into pET28a containing a His tag. The *E*. *coli* BL21 strain served to express His-tagged fusion proteins. Protein expression was induced with IPTG (0.1 mM) and purified using affinity chromatography (Ni^2+^ Sepharose 6 Fast Flow column from Cytiva). Protein purity was analysed using 10% SDS-PAGE, while BCA protein assay (Pierce) was used to determine protein concentrations. Rabbit polyclonal antibodies against MptpB were prepared and purified as we described previously.^20^ Recombinant 6 × His-tagged MptpB purified from *E. coli* served as an immunogen for immunising New Zealand rabbits. The antibody specific to MptpB was isolated by passing the immunised rabbit serum on protein A agarose (Sino Biological).

### Detection of actin cytoskeleton remodelling

Host cellular F-actin/G-actin ratios were measured by western blot analysis, as we previously described.^21^ HEK293T cells were plated at a density of 1 × 10^6^ cells per 60-mm dish, then transfected with pcDNA3.1-MptpB or pcDNA3.1 with the FuGENE HD transfection reagent (Promega, #E2311) as per the supplier's protocol. Following a 24-h transfection period, the cells were exposed to 10 μM Cytochalasin D or 50 μM palmitoleic acid for 1 h before harvesting. After two PBS washes, cells were subjected to lysis using buffer 1 (Lys1: 10 mM K_2_HPO_4_, pH 7.0, 100 mM NaF, 2 mM MgCl_2_, 50 mM KCl, 1 mM EDTA, 1 mM sucrose, 0.5% Triton X-100 plus 1% protease inhibitor cocktail) for 10 min, then centrifuged at 20,000 × *g* for 30 min. The resulting supernatant was taken as the soluble G-actin fraction. The insoluble F-actin present in the pellet was re-suspended in buffer 2 (Lys2: 20 mM Tris–HCl, pH 7.5, 5 mM guanidine hydrochloride, 1 mM sodium acetate, 1 mM CaCl_2_, 1 mM ATP plus 1% protease inhibitor cocktail) and left for 2 h at 4 °C with gentle agitation to convert F-actin into a soluble form (G-actin). after centrifugation of the lysate (20,000 × *g*, 30 min, 4 °C), the resulting supernatant was retained for F-actin measurement. Aliquots taken from the supernatant (G-actin) and from the resuspended pellet (F-actin) were then subjected to western blot analysis with the anti-actin antibody C4 (MAB1501, Sigma, RRID:AB_2223041, 1:5000 dilution).

### Measurement of IκBβ degradation

HEK293T cells were plated into 6-well plates at 2 × 10^6^ cells per well, then transfected with either pcDNA3.1-MptpB or pcDNA3.1 using FuGENE HD as per the supplier's protocol. Following a 24-h transfection period, the cells were incubated in the presence or absence of palmitoleic acid. Cells either remained untreated or were exposed to 20 ng/mL TNF-α (Sino Biological) over various durations. To prepare samples for western blotting, cells were disrupted using a lysis buffer composed of 25 mM Tris–HCl (pH 7.4), 150 mM NaCl, 1% NP-40, 1 mM EDTA, and 5% glycerol, plus 1% protease inhibitor cocktail (Pierce). Identical quantities of total protein were resolved via SDS-PAGE, then electrotransferred onto PVDF membranes (Bio-Rad). Immunoblotting was performed with the following primary antibodies: anti-6 × His tag (6005-1-Ig, Proteintech, RRID:AB_11232599), anti-α-tubulin (66031-1-Ig, Proteintech, RRID:AB_11042766), anti-actin C4 (MAB1501, Sigma, RRID:AB_2223041), and anti-IκBβ (8635, Cell Signalling Technology, RRID:AB_11141830).

### Detection of NF-κB signalling

Raw 264.7 cells were plated into 6-well plates at 1 × 10^6^ per well, then transfected with either pcDNA3.1-MptpB or empty pcDNA3.1 (Vector) using FuGENE HD as per the supplier's protocol. Following a 24-h transfection period, the cells were stimulated with 1 μg/mL lipopolysaccharide (LPS) for 3 h or with 10 ng/mL IFN-γ for 6 h to activate NF-κB. The assay was performed in triplicates in at least three experiments. NF-κB activation was assessed by quantifying *tnf-α*, *il-6* and *il-1β* mRNA levels. Primer sequences are provided in Table S1.

### Inhibition assays

The phosphatase activity of MptpB was determined as previously described, using a 200-μL reaction mix that contained 1.5 μg of MptpB protein in 50 mM Tris/100 mM sodium chloride buffer (pH 7.0) along with various concentrations of palmitoleic acid (0–50 μM). The mixture was incubated for 10 min at room temperature, after which p-nitrophenyl phosphate (pNPP) was added to reach a final concentration of 1.3 mM, followed by an additional 5-min incubation. Absorbance at 405 nm was then recorded in 96-well plates using an Infinite M200 PRO microplate reader (Tecan). The non-enzymatic hydrolysis of pNPP was corrected by controls reactions without MptpB. The inhibition rate was calculated by comparing its reaction rate with the no-inhibition control. All assays were performed at least triplicates.

### Proteomic analysis

To explore how MptpB functions during BCG-macrophage interactions, the murine monocyte-macrophage cell line J774.1 was infected with both mptpB-knockout (ΔmptpB) and wild-type (WT) BCG strains. Cells were harvested at 12, 24, and 48 h after infection for proteomic analysis. Cells were harvested 12, 24, and 48 h post-infection for proteomic analysis. The preparation of proteomic samples and LC-MS analyses were conducted as we previously described.^22^^,^^23^ Raw MS data files underwent processing with the commercial software Proteome Discoverer v2.4 (Thermo Fisher Scientific). Database searches were performed against *Mus musculus* in the SWISS-PROT database using the Sequest HT search engine. Significant differential expression was defined as a fold change ≥2 and an adjusted P-value ≤0.05 for the proteins. PCA analysis, KEGG enrichment, and GO enrichment were performed to determine the functional pathway of MptpB perturbation.

In the microassay screening for human interactome proteins of MptpB, a pure protein sample expressed and purified in our laboratory as previously described^19^^,^^20^ was subjected to buffer exchange using Amicon® Ultra-4 3kD filters, followed by quantification and biotinylation according to the Full Moon labelling kit protocol. The HuProt™ human proteome chip, encompassing approximately 20,000 full-length human proteins, was used for the interaction study.^24^ The chip was blocked, and the biotinylated MptpB sample (20 μg) was hybridised onto it, followed by extensive washing and scanning using a GenePix 4000 B scanner at 635 nm. Data analysis involved quality control assessments using GenePix Pro v6.0 software, normalisation, and identification of protein spots with significant interaction signals. A total of 40,480 spots representing 20,240 proteins were analysed, and proteins that interacted with MptpB were identified after applying a stringent cut-off. Subsequent bioinformatics analyses, including GO, KEGG Pathway, and GSEA, were conducted to elucidate the functional attributes of these interacting proteins and their relevance to MptpB and to screen the functional categories enriched by MptpB-interacting proteins.

### Data analysis

In vivo animal experiments included at least two biological replicates, whereas in vitro assays (including cell, bacterial and molecular studies) were conducted at least three biological replicates. Figure legends indicate the number of animals used in each study (n). Based on the resource equation method,^25^ a sample size of n = 6 mice per group was calculated. This sample size also falls within the range reported in high-impact tuberculosis studies.^9^^,^^26^ Mice were randomly assigned to groups. Blinding was maintained during vaccination, infection, and all downstream analyses. No animals were excluded from analysis. Each dot or lane represents a single mouse or a sample. Unless specified otherwise, values are shown as mean ± SD (see figure legends), and each experimental group was compared against its corresponding control.

### Statistics

Statistical analyses were performed using GraphPad Prism 6 software (GraphPad Software, USA). Distributional assumptions were assessed using the Shapiro–Wilk test, and based on this evaluation the statistical method was selected according to the experimental design and sample size. For simple two-group comparisons, normality tests have limited power and variables such as CFU counts and histology scores are inherently skewed; therefore, the Mann–Whitney test was applied as a conservative non-parametric method. Results for two-group comparisons are presented as mean ± SD together with individual data points (dot plots) to allow direct visual comparison across panels while fully revealing the underlying distribution. For multi-group or factorial designs involving three or more groups or multiple time points, one-way or two-way ANOVA with Tukey's correction for multiple comparisons was used to examine main effects and interactions, where data met normality and variance assumptions after assessment. These designs were fully balanced with equal sample sizes across groups, and the combined sample size provides robustness against mild violations of normality via the central limit theorem. Correlation analyses were uniformly performed using Spearman's rank correlation. ∗P < 0.05, ∗∗P < 0.01, ∗∗∗P < 0.001, ∗∗∗∗P < 0.0001; ns indicates not significant.

### Role of funders

The funders did not participate in the study design, data collection, analysis, result interpretation, or manuscript preparation.

## Results

### Efficacy of BCG vaccine is influenced by the difference of the baseline level of gut microbiota

**Fig. 1:**
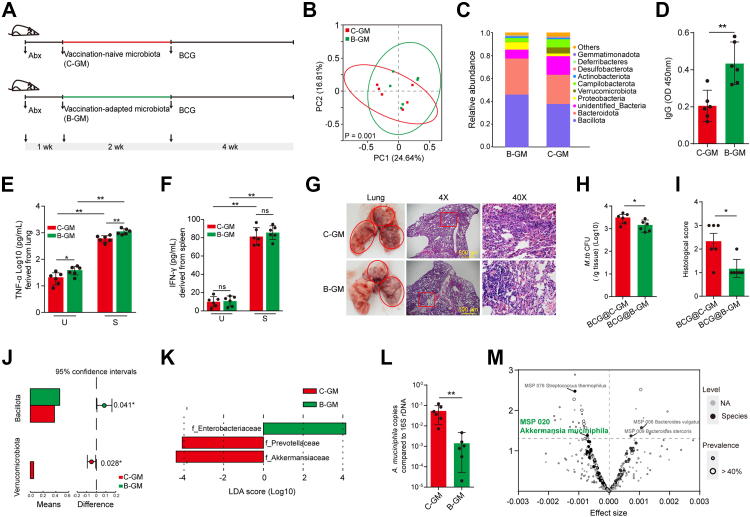
**BCG vaccine-induced immune protective responses were related to the differences in gut microbial abundance before vaccination.** (A) Experimental schema of mouse model to evaluate the influence of gut microbiota. (B) PCoA plot of gut microbiota in B-GM and C-GM treated mice before BCG vaccination. (C) Proportional distribution of intestinal bacterial taxa at the phylum rank. (D) Mycobacteria-specific IgG concentrations in sera of mice that received BCG vaccine after 4 weeks. (E and F) The levels for TNF-α in lung (E) and IFN-γ in spleen (F) cultured with (S) or without (U) Mtb lysate stimulation. (G) Haematoxylin and eosin (H & E) staining of two typical lungs derived from BCG-vaccinated mice (BCG@B-GM and BCG@C-GM) infected with Mtb for 5 weeks. (H and I) Numbers of intracellular Mtb CFU (H) and histological scores (I) in lung tissue. (J) Bacteria showing significant differences at phylum level. (K) LEfSe identified taxonomic biomarkers. (L) Quantification of *A. muciniphila* levels in faecal specimens before vaccination. (M) The associations between microbes and Mtb-specific responses were assessed using a linear model, and significant species were identified based on P < 0.05 and prevalence >40%. Data are presented as a mean ± SD. In vivo animal experiments included at least two biological replicates. N = 6 per group in the mouse model. N = 321 human subjects (M). Mann Whitney test was used to assess statistical significance for [(C), (D), (E), (F), (H), (I), (J), and (L)]. Permutation multivariate analysis of variance (PERMANOVA) test for (B). ∗P < 0.05, ∗∗P < 0.01, ∗∗∗P < 0.001, and ∗∗∗∗P < 0.0001; ns, not significant.

To address whether the difference of baseline gut microbial levels pre-vaccination affects the efficacy of BCG vaccine, we used a microbiota transplant approach in specific pathogen-free (SPF) mice, a physiologically relevant model with an established gut–immune axis.^12^^,^^15^ Co-housed littermate mice received a one-week course of broad-spectrum antibiotics to deplete the original intestinal microbial flora, then were transplanted with gut microbiota from vaccination-naive (C-GM) or vaccination-adapted (B-GM) hosts over the next 2 weeks before vaccination with BCG, allowing us to assess the causal role of pre-existing gut microbiota composition difference in shaping vaccine responses (Fig. 1A). Principal co-ordinates analysis (PCoA) demonstrated that the bacterial community in B-GM mice was distinct (*P* = 0.001) compared to control mice at the pre-vaccination time point (two weeks after transplantation), despite some overlap (Fig. 1B). In B-GM-colonised mice, the alpha diversity index revealed lower microbial richness and the abundance of bacterial phyla was also reduced compared to C-GM mice (Fig. 1C; Fig. S1A and B). These data showed that before vaccination, the difference in the baseline levels of gut microbiota has been established between the two groups of mice.

We next analysed the immune responses of B-GM- and C-GM-transplanted mice after receiving BCG vaccine for 4 weeks. Vaccine-mediated immunity is characterised by the induction of antibody responses.^12^ Thus, we first analysed the antibody response and found that although both groups of mice mounted a response to BCG vaccination, BCG-induced IgG were more significantly activated in B-GM-transplanted mice compared to those in C-GM-transplanted mice (Fig. 1D). As T cell-mediated immunity is crucial for TB vaccine protection, we first analysed by flow cytometry the responses of CD4^+^ and CD8^+^ T cells. The results showed that baseline microbiota differences significantly altered CD4^+^ and CD8^+^ TNF-α responses after BCG vaccination, whereas CD4^+^ and CD8^+^ IFN-γ responses did not differ between groups (Fig. S2). We then monitored the release of TNF-α and IFN-γ by cells derived from mouse tissues stimulated with Mtb lysates because these secreted cytokines serve as indicators of immune memory response activation during BCG vaccination.^15^ According to the results, the production of TNF-α was also higher in lung single-cell suspensions from B-GM colonised mice than in those from C-GM colonised mice, and this increase of TNF-α showed at least partial specificity towards Mtb because ex vivo re-stimulation using Mtb lysates led to a further enhancement of its production (Fig. 1E). No significant difference in IFN-γ secretion was observed between B-GM colonisation mice and C-GM colonisation mice before and after stimulation with Mtb lysates (Fig. 1F). These results indicate that the baseline differences in gut microbiota underlie the observed disparity in immune outcomes, suggesting that the pre-existing microbiota modulates the immune response to BCG.

We also investigated whether such immune response of the two groups after vaccinated with BCG affects Mtb clearance from the host. To this end, the BCG vaccinated mice BCG@C-GM and BCG@B-GM were subsequently infected with Mtb for 5 weeks. Consistent with the induced protective phenotype of BCG, although all mice were BCG-vaccinated and thus protected, the BCG@B-GM group exhibited markedly reduced haemorrhage and pathological damage within the lungs than did the BCG@C-GM control group (Fig. 1G; Fig. S3). Correspondingly, the BCG@B-GM group exhibited lower pulmonary bacillus burden and reduced pathological damage to the lungs (Fig. 1H and I). These data confirmed that gut microbiota profile in pre-vaccination B-GM group has a better vaccine efficacy of BCG.

Next, we characterised the gut microbiota features of pre-vaccination mice with an established gut microbiome. Compared with that in C-GM mice, Verrucomicrobia abundance decreased while Bacillota abundance increased in B-GM mice (Fig. 1J). Similarly, LEfSe analysis used to identify the bacterial taxa between the two groups showed a significant change in the Enterobacteriaceae, Prevotellaceae and Akkermansiaceae abundance at the rank of the bacterial family between the two groups (Fig. 1K), among which *A. muciniphila*, belonging to the family Akkermansiaceae, was the most significantly enriched species in C-GM group (namely decreased in B-GM mice) (Fig. S1C–H). Furthermore, the reduced abundance of *A. muciniphila* in pre-vaccination B-GM was confirmed by *A. muciniphila*-specific qPCR (Fig. 1L). Faecal sequencing detected no BCG reads in vaccination-adapted samples (Table S3), confirming that the observed immune differences are due to gut microbiota rather than residual BCG exposure. These suggested that before vaccination, a higher baseline level of *A. muciniphila* is associated with a decreased effectiveness of BCG.

We further investigated the relationship between the gut microbiota and the immune response to mycobacteria in human subjects. We analysed a public data^13^ investigating the impact of the gut microbiota in modulating immune memory responses within 321 healthy subjects following BCG vaccination. Faecal samples were obtained from healthy adults prior to BCG immunisation to study microbiome composition, with blood draws then performed at 2 weeks and 3 months respectively post vaccination to measure cytokine production following ex vivo Mtb stimulation. The results showed that *A. muciniphila* abundance was negatively associated with BCG vaccine-induced immunity. Specifically, it exhibited a significant negative correlation with specific immunity (P < 0.05) and an approaching significant negative correlation with trained immunity (P = 0.09), with a prevalence >40% (Fig. 1M; Fig. S4A). No marked association was observed between the levels of *A. muciniphila* and the secretion of IFN-γ or IL-6, whereas TNF-α was absent in Mtb stimulation (Fig. S4B). Interestingly, the cytokine production in the supernatant of PBMCs in our data showed that compared with healthy controls without mycobacteria infection, the individuals with mycobacteria infection showed elevated TNF-α levels, rather than IFN-γ; this response was additionally correlated with the reduced *A. muciniphila* abundance when ex vivo re-stimulated with Mtb lysates (Fig. S4C–E).

These data collectively indicate that the baseline levels of gut microbiota can mediate the efficacy of vaccines, and highlight *A. muciniphila* as a potential immunomodulatory species associated with BCG vaccination.

### Higher baseline level of pre-vaccination gut *A. muciniphila* leads to poorer vaccine responses

**Fig. 2:**
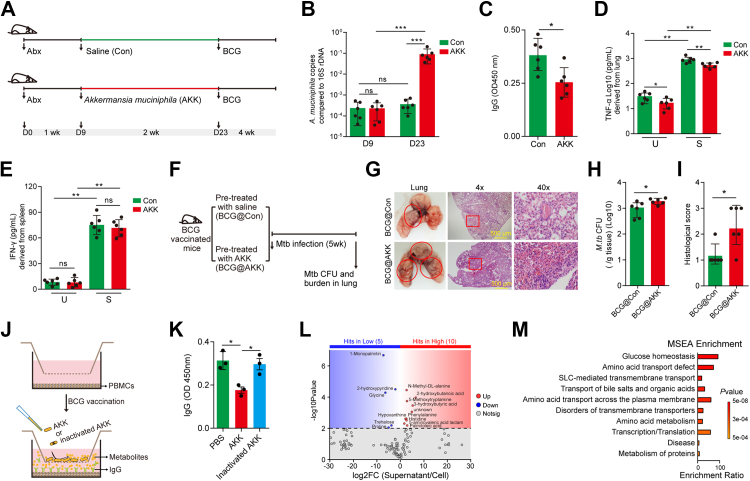
**The abundance of *A. muciniphila* before vaccination mediates the protective efficacy conferred by BCG vaccine, which is associated with the function of metabolites.** (A) Experimental schema of mouse model for assessing the influence of *A. muciniphila* (AKK) before vaccination on BCG vaccine-induced immune response. (B) *A. muciniphila* abundance in stool samples. (C) Mycobacteria-specific IgG concentrations in sera of mice that received BCG vaccine after 4 weeks. (D–E) Expression levels of TNF-α in lung (D) and IFN-γ in spleen (E) stimulated with (S) or without (U) Mtb lysates. (F) Assessment of protective efficacy of BCG-vaccinated mice (BCG@Con and BCG@AKK) after infected with Mtb for 5 weeks. (G) H&E staining of two typical lungs. (H–I) Numbers of intracellular Mtb CFU (H) and histological scores (I) in lung tissue. (J) Schematic representation of transwell setup for simulating the impact of *A. muciniphila* on IgG production of PBMCs derived from mouse. (K) IgG levels in conditioned media from PBMCs treated with *A. muciniphila*. (L) Differential metabolites in the supernatant from *A. muciniphila* versus its bacterial cell fractions (*P* < 0.01). (M) MSEA analysis of the function of *A. muciniphila*-derived metabolites on the host. Data are presented as a mean ± SD. In vivo animal experiments included at least two biological replicates, whereas in vitro assays were conducted at least three biological replicates. N = 6 per group in the mouse model. Mann Whitney test was used to assess statistical significance for [(C), (D), (E), (H), and (I)]. One-way ANOVA with Tukey correction for (K), and two-way ANOVA with Tukey correction for (B). ∗P < 0.05, ∗∗P < 0.01, ∗∗∗P < 0.001, and ∗∗∗∗P < 0.0001; ns, not significant.

We hypothesised that the difference in the baseline level of *A. muciniphila* in the gut of pre-vaccination mice is a factor leading to the variation in immune response during subsequent BCG vaccination. To address this, mice depleted the original gut microbiota received by oral gavage *A. muciniphila* or saline before BCG vaccination (Fig. 2A). As expected, increased abundance of *A. muciniphila* was verified via qPCR targeting this bacterium (Fig. 2B). The two group subsequently received the same treatment with BCG vaccine. Mice pre-treated with *A. muciniphila* showed a significantly impaired IgG antibody response to BCG vaccination compared with the control group (Fig. 2C). Similarly, *A. muciniphila* treatment resulted in reduced CD4^+^ and CD8^+^ TNF-α responses and lower levels of TNF-α expression, both of which were impaired following Mtb lysate stimulation (Fig. 2D; Fig. S5A–D). The specific response, assessed via IFN-γ levels, showed no difference with or without Mtb stimulation between the treatment of *A. muciniphila* and the control (Fig. 2E). These results reveal that before vaccination, high abundance of *A. muciniphila* diminished BCG-induced immune response to the vaccine. BCG efficacy in *A. muciniphila* (BCG@AKK) and saline (BCG@Con) mice in providing protection against Mtb infection further confirmed our findings (Fig. 2F). The group of BCG@AKK caused more lung haemorrhage and tissue damage, along with a higher pulmonary bacilli burden compared with the control group (Fig. 2G–I). Notably, body weight changes at different stages throughout the experiment did not differ significantly between the two groups (Fig. S5E–H), suggesting that the observed differences in vaccine-induced protection were not attributable to body weight-related metabolic phenotypes.

We then investigated how *A. muciniphila* mediates the vaccine response. PBMCs derived from mice were inoculated with BCG and then exposed to either live or pasteurised inactivated *A. muciniphila*, respectively (Fig. 2J). Treatments of live *A. muciniphila* significantly reduced the IgG concentrations induced by BCG vaccination (Fig. 2K), suggesting that metabolite(s) produced by *A. muciniphila* plays the role in affecting BCG vaccine response. To identify the metabolite(s), untargeted metabolomics analysis of culture supernatant and bacterial cells of *A. muciniphila* were performed. The results showed that the supernatants harboured a significantly distinct metabolite profile compared to the cells, and ten secreted metabolites and five bacterial metabolites were identified (*P* < 0.01) (Fig. 2L; Table S4). To understand the potential impact of these *A. muciniphila*-derived metabolites on the host environment encountered by BCG, we performed MSEA analysis. The result revealed that the pathways influenced by these metabolites mainly focused on glucose homoeostasis, amino acid transport, transmembrane transport, and metabolism of proteins (Fig. 2M). These pathways represent physiological processes that are required for the interaction between mycobacteria and the host, thereby highlighting the potential of *A. muciniphila*-derived metabolites to modulate the BCG-induced immune response by altering the host metabolic landscape.

Taken together, these data indicate that higher baseline level of *A. muciniphila* in pre-vaccination mice results in poorer efficacy of BCG vaccine, which is associated with the function of metabolites of gut bacteria.

### *A. muciniphila-*contributed gut co-metabolite palmitoleic acid inhibits the vaccine responses of BCG

**Fig. 3:**
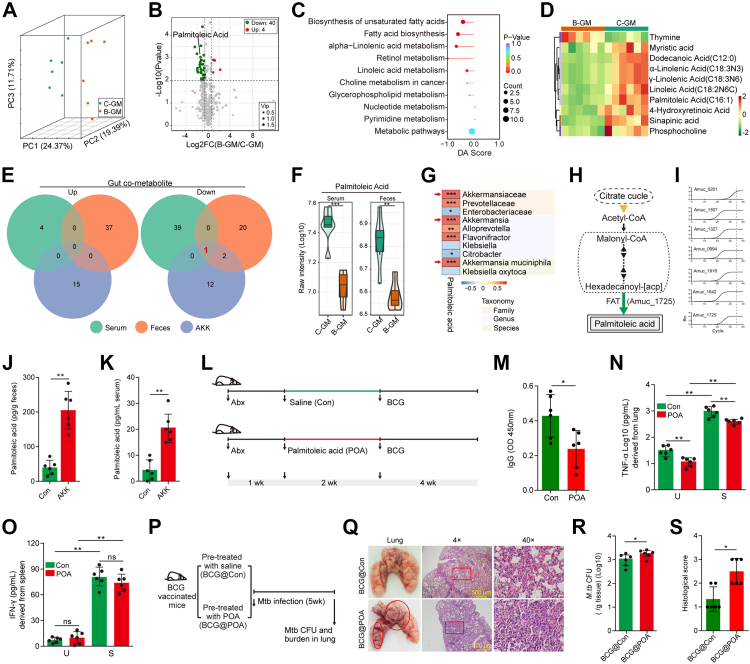
**The metabolic differences of the pre-vaccination host, particularly gut co-metabolite palmitoleic acid contributed by *A. muciniphila*, affects the protective efficacy of BCG vaccine.** (A) PCA plot depicting serum metabolite profiles of mice treated with C-GM or B-GM prior to BCG immunisation. (B) Volcano plot displaying all identified serum metabolites, where red and green symbols denote significantly differential metabolites. (C) KEGG enrichment analysis of metabolites showing differential enrichment. (D) Heatmap of KEGG enriched differentially expressed metabolites. (E) Venn diagram showing the only gut co-metabolite in sera directly associated with *A. muciniphila* levels. (F) Palmitoleic acid levels in the serum and faecal samples. (G) Heatmap illustrating the relationship between palmitoleic acid levels and the relative quantities of LEfSe-identified taxa (family, genus and species levels). (H) Schematic diagram for palmitoleic acid biosynthesis pathway in *A. muciniphila*. (I) The presence of genes encoding the palmitoleic acid biosynthetic pathway in *A. muciniphila* was verified using bacterial mRNA. (J–K) Oral administration of *A. muciniphila* (AKK) led to markedly higher 3HB levels in faecal (J) and serous (K) specimens of mice prior to BCG immunisation. (L) Experimental schema of mouse model to evaluate the influence of palmitoleic acid (POA) before vaccination on BCG vaccine-induced immune response. Mice received palmitoleic acid supplementation limited to the pre-vaccination period (for two weeks), which was terminated at the time of BCG vaccination. (M) IgG concentrations in sera of mice that received BCG vaccine after 4 weeks. (N–O) Expression levels of TNF-α in lung (D) and IFN-γ in spleen (E) stimulated with (S) or without (U) Mtb lysates. (P) Assessment of protective efficacy of BCG-vaccinated mice (BCG@Con and BCG@POA) after infected with Mtb for 5 weeks. (Q) H&E staining of two typical lungs. (R–S) Numbers of intracellular Mtb CFU (H) and histological scores (I) in lung tissue. Data are presented as a mean ± SD. In vivo animal experiments included at least two biological replicates, whereas in vitro assays were conducted at least three biological replicates. N = 6 per group in the mouse model. Mann Whitney test was used to assess statistical significance for [(F), (J), (K), (M), (N), (O), (R), and (S)]. Spearman correlation for (G). ∗P < 0.05, ∗∗P < 0.01, ∗∗∗P < 0.001, and ∗∗∗∗P < 0.0001; ns, not significant.

Given the established link between gut *A. muciniphila* and its metabolites in the BCG vaccine responses (Fig. 2J–M) and the fact that changes of microbiota are linked to alterations of metabolites in the gut, we examined the metabolome of the faeces and sera of B-GM and C-GM transplanted mice before BCG vaccination. LC-MS analysis of serum metabolites was carried out firstly. An analysis of rarefaction comparing intra-subject metabolite diversity showed that B-GM animals harboured a dissimilar metabolite profile relative to C-GM animals (Fig. 3A). Volcano plot integrating OPLS-DA (VIP >1) and t-test (P < 0.01) results revealed 44 significantly altered metabolites (Fig. 3B; Fig. S6A–C). Differential metabolite cluster analysis showed a primary change in the serum of B-GM mice was a marked reduction in many fatty acids, which were positively correlated with each other (Fig. S6D and E). KEGG pathway analysis indicated that fatty acid biosynthesis represented a prominently altered pathway in these mice (Fig. 3C). Nine fatty acids, including palmitoleic acid, were enriched in these KEGG pathway (Fig. 3D; Fig. S6F).

We subsequently assessed the faecal metabolite profiles and found that the profiles of B-GM and C-GM were distinct (Fig. S7A and B). Sixty metabolites exhibiting notable alterations and elevated VIP values were identified and were closely correlated with each other (Fig. S7C–F; Table S5). Examination of differentially expressed metabolites in sera and faeces showed that palmitoleic acid was the only core metabolite with reduced abundance in B-GM mice, and exhibited a significant statistical correlation of its levels between faecal and serum samples (Fig. S7G and H). The reduced concentration of palmitoleic acid in faeces was significantly linked to the levels of the identified intestinal microbial community, particularly *A. muciniphila* (Fig. 3E–G). Additionally, the findings indicated that the levels of palmitoleic acid rose within the culture supernatants from *A. muciniphila* (Fig. 2L). KEGG and qPCR analyses confirmed that *A. muciniphila* is capable of producing and accumulating palmitoleic acid (Fig. 3H and I). Indeed, gavage of *A. muciniphila* markedly raised the levels of palmitoleic acid within faeces as well as serum from mice (Fig. 3J and K). These findings indicate that *A. muciniphila*-derived palmitoleic acid might be involved in modulating of BCG.

We thus tested whether the increased baseline level of palmitoleic acid in pre-vaccination mice exert an inhibitory effect on vaccine efficacy of BCG. To resolve this, the mice underwent prior treatment with palmitoleic acid or saline during the two weeks before BCG vaccination (Fig. 3L). Compared to the control group, treatment before BCG vaccination markedly raised palmitoleic acid levels in both faeces as well as serum within the normal physiological range (Fig. S8). The results showed that the mice pre-treated with palmitoleic acid exhibited a significantly impaired IgG antibody response, reduced CD4^+^ and CD8^+^ TNF-α responses, and lower levels of TNF-α expression upon re-stimulation with or without Mtb lysates, with no difference in IFN-γ levels (Fig. 3M–O; Fig. S9). The BCG efficacy caused by palmitoleic acid (BCG@POA) and saline (BCG@Con) in providing protection against Mtb infection further confirmed our findings (Fig. 3P). The group of BCG@POA caused more lung haemorrhage and tissue damage along with showing a higher lung bacilli burden compared with the control group (Fig. 3Q–S). These results were similar to those of *A. muciniphila* treatment (Fig. 2), suggesting that palmitoleic acid produced by *A. muciniphila* is involved in modulating the vaccine response to BCG.

Collectively, these data suggest that the differences of gut co-metabolite of the pre-vaccination host, particularly palmitoleic acid contributed by *A. muciniphila*, affects the efficacy of BCG vaccine.

### Palmitoleic acid acts on vaccine response through the mediation of MptpB of BCG

**Fig. 4:**
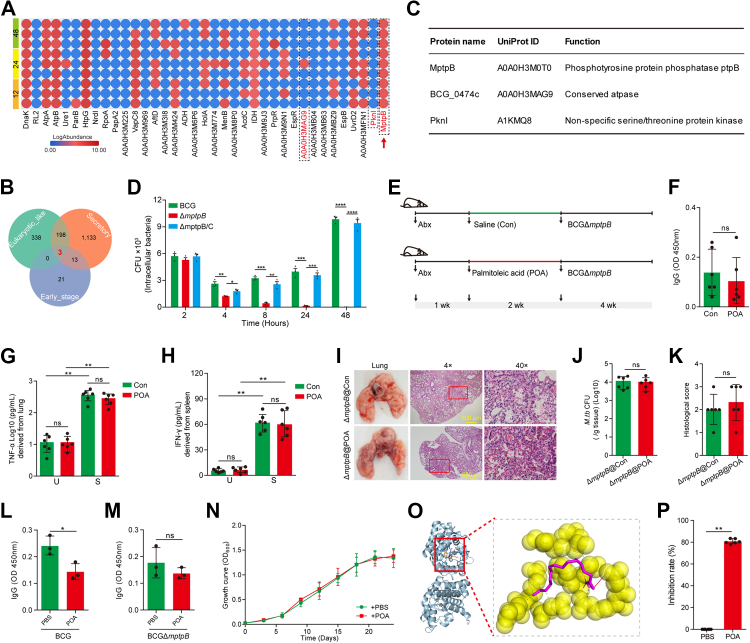
**The function of palmitoleic acid on BCG vaccination was mediated by the effector protein MptpB of BCG.** (A) Quantification of BCG proteins in mouse J774A.1 macrophages at 12, 24, 48 h post vaccination. (B) Venn diagram showed the identified BCG proteins in host cells during the early stage and secreted proteins and proteins carrying eukaryotic-type motifs or structures. (C) Description of three secreted proteins harbouring eukaryotic-like motifs. (D) Survival of intracellular bacteria in primary human monocyte-derived macrophages infected for 48 h with wild-type BCG (BCG), *mptpB*-deleted strain BCGΔ*mptpB* (Δ*mptpB*), or *mptpB*-complemented strain Δ*mptpB*/C. (E) Schematic diagram of MptpB in the function of palmitoleic acid (POA) to BCG. (F) IgG concentrations in sera of mice that received BCGΔ*mptpB* vaccine after 4 weeks. (G–H) Expression levels of TNF-α in lung (G) and IFN-γ in spleen (H) stimulated with (S) or without (U) Mtb lysates. (I) H&E staining of two typical lungs derived from BCGΔ*mptpB*-vaccinated mice (Δ*mptpB*@Con and Δ*mptpB*@POA) after infected with Mtb for 5 weeks. (J–K) Numbers of intracellular Mtb CFU (H) and histological scores (I) in lung tissue. (L–M) IgG concentrations in culture supernatants of PBMCs inoculated with BCG or BCGΔ*mptpB* after treated with palmitoleic acid. (N) In vitro growth curve of BCG in 7H9 broth treated with or without palmitoleic acid (POA). (O) Binding mode of MptpB (yellow) to palmitoleic acid (pink). (P) Inhibition of MptpB enzyme activity by palmitoleic acid. Data are presented as a mean ± SD. In vivo animal experiments included at least two biological replicates, whereas in vitro assays were conducted at least three biological replicates. N = 6 per group in the mouse model. Mann Whitney test was used to assess statistical significance for [(F), (G), (H), (J), (K), and (P)]. Student's *t*-test for (L) and (M). Two-way ANOVA with Tukey correction for (D). ∗P < 0.05, ∗∗P < 0.01, ∗∗∗P < 0.001, and ∗∗∗∗P < 0.0001; ns, not significant.

We next investigated how palmitoleic acid efficiently affects vaccine responses of BCG vaccination. Given that the host pathway influenced by palmitoleic acid is related to transmembrane transport and metabolism of protein, we analysed proteomes of host cells infected with BCG during the early stage at 12, 24, and 48 h respectively to identify the potential bacterial components derived from BCG. We found 37 proteins of BCG in host cells (Fig. 4A). Among them, three proteins were classified as BCG-secreted eukaryotic-like proteins which could act on host pathways directly (Fig. 4B). MptpB, a phosphatase essential for mycobacterial survival within host macrophages, was the most abundant protein during infection (Fig. 4C). Thus, we focused on MptpB and confirmed its role in promoting BCG survival during the early stages in primary human monocyte-derived macrophages (Fig. 4D). Furthermore, deletion of the *mptpB* gene compromised BCG survival in activated macrophages, while its survival in resting macrophages remained unchanged, underscoring the contribution of this gene to vaccine efficacy (Fig. S10). Indeed, as a live bacterial vaccine, BCG is capable of surviving within the host after vaccination, thereby inducing protective immunity against subsequent Mtb infection.^3^^,^^27^ In contrast, dead or inactivated mycobacteria or bacterial components did not induce protective immunity.^28^^,^^29^ Thus, MptpB that supports the survival of BCG within the host during the early stages is essential for successful immunisation, as demonstrated by impaired vaccine responses of BCGΔ*mptpB*-vaccinated mice compared to BCG-vaccinated mice (Fig. S11).

We next investigated whether the inhibitory effect of palmitoleic acid on vaccine responses in BCG-vaccinated hosts is related to MptpB. The experimental schema was the same as that shown in Fig. 3L, except for the use of BCGΔ*mptpB* instead of BCG (Fig. 4E). No significant disparity was found in the IgG antibody response, CD4^+^/CD8^+^ TNF-α and IFN-γ responses, or TNF-α/IFN-γ production with or without Mtb lysate stimulation between mice pre-treated with palmitoleic acid and saline (Fig. 4F–H; Fig. S12). No difference in haemorrhage and pathological impairment of the lungs and pulmonary bacilli burden was also observed in the group of Δ*mptpB*@POA and Δ*mptpB*@Con (Fig. 4I–K). Indeed, PBMCs inoculated with BCG or BCGΔ*mptpB* and then treated with palmitoleic acid showed no difference in the production of IgG in BCGΔ*mptpB* group (Fig. 4L and M). These findings revealed that the inhibitory effect of palmitoleic acid on vaccine responses to BCG vaccination is mediated by MptpB.

We next aimed to elucidate how palmitoleic acid specifically suppresses MptpB. First, palmitoleic acid had no direct bactericidal effect because it did not suppress the survival or growth rate of BCG cells (Fig. 4N). Second, the molecular docking results indicated that, among the differential metabolites, palmitoleic acid exhibited a strong interaction with MptpB (Fig. 4O; Fig. S13). Third, when incubated with palmitoleic acid, the phosphatase activity of MptpB exhibited marked dose-dependent inhibition, likely via multiple interaction sites and residues (Fig. 4P; Fig. S14). These data strongly suggest that palmitoleic acid may exert its effects on BCG through MptpB-mediated pathways.

Collectively, these results demonstrate that the role of palmitoleic acid on BCG vaccination is directly mediated by the effector protein MptpB of BCG and is related to the host pathway targeted by MptpB.

### MptpB regulates actin cytoskeleton remodelling to activate BCG vaccine responses

**Fig. 5:**
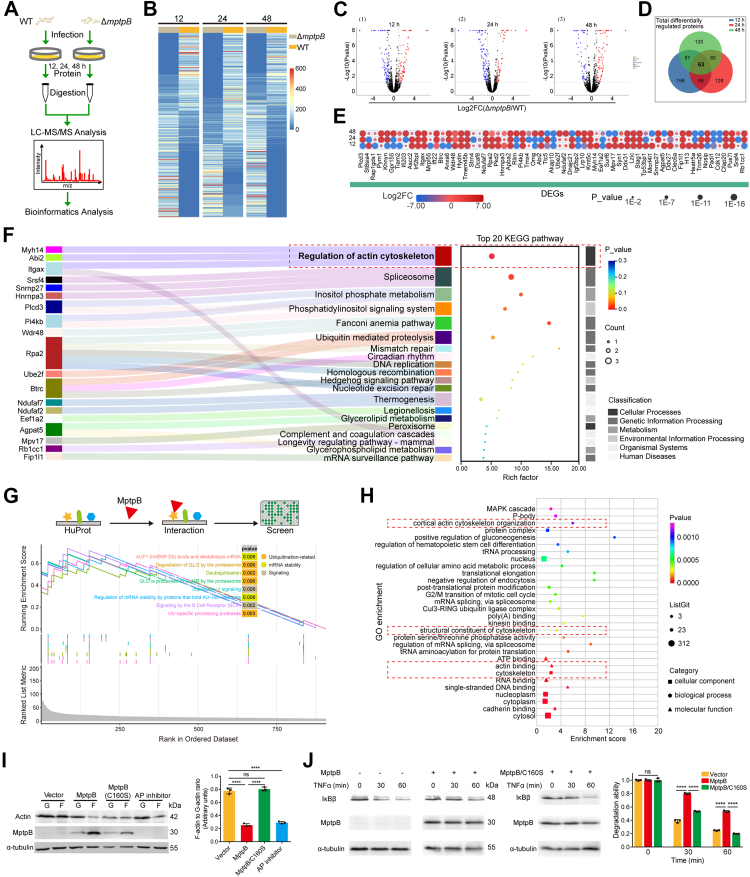
**Proteomic analysis of MptpB-induced signalling reveals actin cytoskeleton remodelling to be a pathway underlying its function.** (A) Schematic representation of the workflow of the quantitative proteome in BCG- or BCGΔ*mptpB*-infected J774A.1 macrophages in the early stages at 12, 24, and 48 h. (B) Quantification of MptpB-regulated proteins at indicated time points. (C) Significant differentially expressed proteins with an adjusted P < 0.05 and above or below the threshold of +1 and −1 log2(Δ*mptpB*/WT). (D) Venn diagram showed the proteins regulated by MptpB at all three time points. (E) Heatmap dynamic comparison of the significant proteins at 12, 24, and 48 h. (F) KEGG enrichment results for the top 20 pathways affected by MptpB. (G) GSEA enrichment plot of HuProt™ human proteome microarray identified human proteins that interacted with MptpB. (H) Functional enrichment analysis of MptpB-interacting proteins. (I) Western blotting determination of F-actin and G-actin levels and their ratio in HEK293T cells treated with Vector, MptpB, and MptpB(C160S) (enzyme activity-inactive variant). Cytochalasin D, an actin polymerisation inhibitor, was used as positive control. (J) Western blotting quantification of IκBβ by MptpB in HEK293T cells stimulated with TNF-α. Data are presented as a mean ± SD. In vitro assays were conducted at least three biological replicates. One-way ANOVA with Tukey correction for (I), and two-way ANOVA with Tukey correction for (J). ∗P < 0.05, ∗∗P < 0.01, ∗∗∗P < 0.001, and ∗∗∗∗P < 0.0001; ns, not significant.

To uncover the pathways regulated by MptpB during the early stage of BCG vaccination within host cells, we analysed three proteomes of host cells infected with BCG (WT) and BCGΔ*mptpB* (Δ*mptpB*) at 12, 24, and 48 h using label-free quantitative proteomics for assessing protein abundance levels (Fig. 5A). We identified 337, 310, and 300 proteins that significantly differentially expressed at 12, 24, and 48 h, respectively (Fig. 5B and C). Among these, 63 proteins were differentially expressed at all three time points, suggesting their role in MptpB regulation to promote BCG survival within host cells (Fig. 5D and E). Several actin cytoskeleton-related GO terms in the functional categories were observed at all three time points (Fig. S15), and the Voronoi treemap for the visualisation of the functional relatedness of these proteins demonstrated that most were related to the categories of actin cytoskeleton, protein modification, and signalling (Fig. S16A). Furthermore, KEGG enrichment analysis to determine the functional pathway of MptpB perturbation revealed that the pathway regulating the actin cytoskeleton was the most significantly enriched (Fig. 5F), and three enriched proteins in this pathway were also highly expressed in the lung tissue of human compared with other tissues (Fig. S16B), further suggesting the potential role of the pathway affected by these proteins in pulmonary diseases, such as TB.

We also screened a human proteome microarray (HuProt™) to identify the host proteins that interact with MptpB. GSEA showed that significant functional categories enriched by MptpB-interacting proteins were primarily responsible for the functions of ubiquitination-related, mRNA stability, and signalling (Fig. 5G). Functional enrichment analysis showed these proteins were also significantly enriched in pathways responsible for actin cytoskeleton regulation (Fig. 5H; Fig. S17A and B), aligning with the findings from proteome profiling of BCG-infected macrophages. A combined analysis of the data of macrophage proteomics and HuProt™ revealed that the proteins involved in the actin cytoskeleton pathway regulated by MptpB interact strongly and tightly (Fig. S17C).

Disruption of the host cell actin cytoskeleton promotes the survival and proliferation of intracellular pathogens, primarily through dynamic conversion between G-actin (globular) and F-actin (fibrous), as indicated by the F-actin/G-actin ratio.^30^^,^^31^ MptpB is a eukaryotic-like protein tyrosine phosphatase and phosphoinositide phosphatase and the Cys160→Ser (C160S) mutation abolishes its enzymatic function.^32^^,^^33^ To investigate how MptpB regulates actin cytoskeleton remodelling, MptpB and C160S mutants were expressed in host cells and the F-actin/G-actin ratio was measured via western blotting. The results demonstrated that MptpB significantly reduced the F-actin/G-actin ratio, while that in cells expressing the C160S mutant protein was like that in the negative control group transfected with the vector alone (Fig. 5I). The activation of NF-κB enables cells to express and release numerous inflammatory cytokines, such as TNF-α/IL-6/IL-1β, aiding the host in resisting and eliminating intracellular bacteria.^34^ We found that MptpB could downregulate the degradation of IκBβ (Fig. 5J), an inhibitor of NF-κB^35^, suppressing host cell NF-κB signalling pathway (TNF-α/IL-6/IL-1β) activation (Fig. S18).

Based on these findings, we assessed whether the efficacy and protective effects of MptpB-mediated BCG vaccination in mice were related to the inhibition of G-actin conversion to F-actin. The vaccine response in BCG-vaccinated mice was blocked by an actin polymerisation inducer, whereas the response in BCGΔ*mptpB*-vaccinated mice was rescued by an actin polymerisation inhibitor (Fig. S19). The vaccinated mice pre-treated with actin polymerisation inducer (BCG@AP inducer) or actin polymerisation inhibitor (Δ*mptpB*@AP inhibitor) were subsequently infected with Mtb for 5 weeks. In BCG-vaccinated mice, the group of BCG@AP inducer showed severe lung infection outcomes relative to the control group. In the BCGΔ*mptpB*-vaccinated mice, the group of Δ*mptpB*@AP inhibitor showed improved lung infection outcomes compared with the control group (Fig. S20). These observations confirmed our hypothesis.

Collectively, these results indicate that MptpB inhibits polymerisation of actin cytoskeleton in hosts, which depends on its enzymatic activity, and confirms that MptpB-mediated inhibition of actin cytoskeleton remodelling is necessary for BCG to activate the vaccine response.

### Actin cytoskeleton remodelling is required for palmitoleic acid to regulate the efficacy of BCG vaccination

**Fig. 6:**
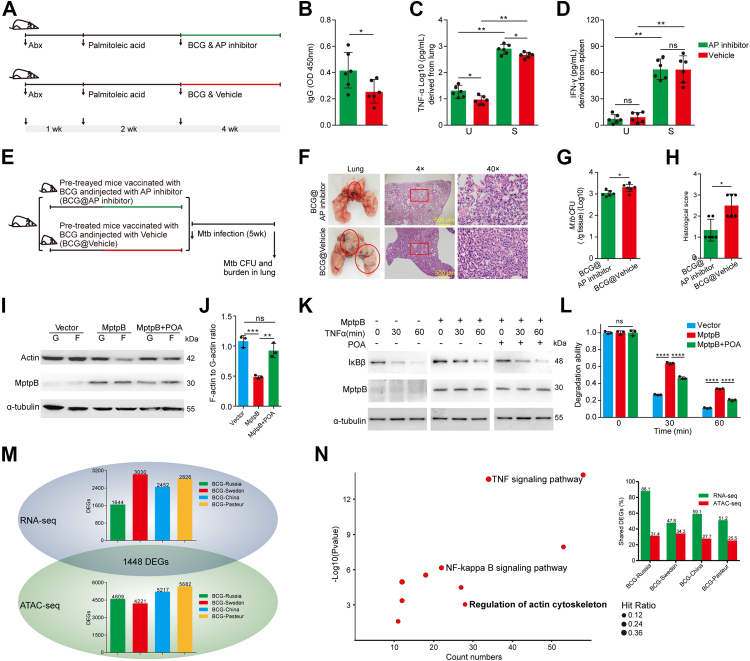
**Inhibition of actin cytoskeleton remodelling blocks the regulatory role of palmitoleic acid on the efficacy of BCG vaccination.** (A) Schematic diagram of the influence of G-actin polymerisation into F-actin inhibition (AP inhibitor) on the function of palmitoleic acid to BCG. (B) IgG concentrations in sera of mice that received BCG and AP inhibitor after 4 weeks. (C–D) Expression levels of TNF-α in lung (C) and IFN-γ in spleen (D) derived from BCG-vaccinated animals cultured with (S) or without (U) Mtb lysate stimulation. (E) Assessment of protective efficacy of BCG-vaccinated mice (BCG@Vehicle and BCG@AP inhibitor) after infection with Mtb for 5 weeks. (F) H&E staining of two typical lungs. (G–H) Numbers of intracellular Mtb CFU (G) and Histological scores (H) in lung tissue. (I–J) Western blotting (I) and quantification (J) of F-actin and G-actin and their ratios in HEK293T cells treated with Vector, MptpB, MptpB plus palmitoleic acid (POA). (K–L) Western blotting (K) and quantification (L) of IκBβ by MptpB and palmitoleic acid (POA) in cells stimulated with TNF-α. (M) Combined ATAC-seq and RNA-seq analyses identified 1448 common genes linked to the induction of immunity and protection across four commonly employed BCG strains (Russia, Sweden, China and Pasteur). (N) Functional enrichment analysis was performed on proteins identified from four representative BCG strains, revealing common signalling pathways that can be induced by different vaccines. Data are presented as a mean ± SD. In vivo animal experiments included at least two biological replicates, whereas in vitro assays were conducted at least three biological replicates. N = 6 per group in the mouse model (A). Mann Whitney test was used to assess statistical significance for [(B), (C), (D), (G), and (H)]. One-way ANOVA with Tukey correction for (J), and two-way ANOVA with Tukey correction for (L). ∗P < 0.05, ∗∗P < 0.01, ∗∗∗P < 0.001, and ∗∗∗∗P < 0.0001; ns, not significant.

Given that the function of palmitoleic acid on BCG vaccination relies on MptpB and that MptpB-mediated inhibition of actin cytoskeleton remodelling is crucial for BCG to activate the vaccine response, we investigated the effect of actin polymerisation inhibition on the efficacy of BCG vaccine with the pre-treatment of palmitoleic acid (Fig. 6A). The actin polymerisation inhibitor rather than the vehicle induced more IgG and higher levels of TNF-α, resulting in an enhanced BCG-induced vaccine response (Fig. 6B–D) and blocked the attenuating effect of palmitoleic acid on the BCG vaccine (Fig. 3). The protective efficacy of these vaccinated mice against Mtb infection also showed that the BCG@AP inhibitor group had significantly reduced pulmonary haemorrhage and pathological damage and led to a lower pulmonary bacillus burden than the control group (Fig. 6E–H). Finally, we demonstrated that palmitoleic acid blocked the ability of MptpB to inhibit the polymerisation of the actin cytoskeleton and delay the degradation of IκBβ (Fig. 6I–L). These data reveal that palmitoleic acid inhibits the function of MptpB-mediated actin cytoskeleton remodelling in BCG to modulate the vaccine efficacy of BCG.

Different BCG strains exhibit varying immunological effects and TB prevention efficacies, which are attributed to their distinct effects on the immune response.^36^ We thus analysed a public data^18^ to investigate differences in the ability and underlying mechanisms to induce vaccine responses across four common BCG strains selected for their usage breadth and virulence, representing all four categories: I (BCG-Russia), II (BCG-Sweden), III (BCG-China), and IV (BCG-Pasteur). Integrated analysis of multi-omics with RNA-seq and ATAC-seq data indicated that the four BCG strains elicited distinct vaccine responses (In BCG-Russia, BCG-Sweden, BCG-China, and BCG-Pasteur, 1644, 3030, 2452, and 2826 differentially expressed genes by RNA sequencing; 4609, 4221, 5217, and 5682 gene chromosome structures altered by ATAC sequencing), there was an intersection of 1448 shared genes (Fig. 6M). KEGG enrichment analysis of shared genes showed significant enrichment of the TNF signalling pathway, NF-κB signalling pathway, and regulation of the actin cytoskeleton identified in our data (Fig. 6N), suggesting a role of these pathways for all BCG strains.

Taken together, higher baseline levels of palmitoleic acid attenuates BCG vaccine efficacy by inhibiting the function of MptpB on actin cytoskeleton polymerisation within the host cells. Most notably, the importance of actin cytoskeleton regulation in maintaining vaccine response during BCG vaccination is collectively emphasised in multi-omics data analyses from our and other studies.

### Inhibition of MptpB or actin cytoskeleton remodelling blocks the effects of higher baseline levels of *A. muciniphila* on the efficacy of BCG vaccination

Given that *A. muciniphila*-derived palmitoleic acid is the dominant metabolite involved in suppressing MptpB-mediated BCG vaccine responses, we hypothesised that the effects of higher baseline level of *A. muciniphila* on the efficacy of BCG vaccination will be blocked by inhibiting the activity of MptpB or actin cytoskeleton remodelling. To address this, mice depleted the original gut microbiota were orally administered *A. muciniphila* or saline before BCG vaccination (Fig. S21A). The experimental schema was the same as that shown in Fig. 2A, except for the use of BCGΔ*mptpB* instead of BCG (Fig. 2). As expected, the baseline level of *A. muciniphila* was increased (Fig. S21B). The two groups subsequently received the same treatment with the BCGΔ*mptpB* vaccine. Comparisons between mice pre-treated with *A. muciniphila* and those given saline revealed no significant differences in either IgG antibody responses or TNF-α/IFN-γ production, irrespective of Mtb lysate stimulation (Fig. S21C–E). The examination of the efficacy of BCGΔ*mptpB* caused by *A. muciniphila* (Δ*mptpB*@AKK) and saline (Δ*mptpB*@Con) in providing protection against Mtb infection showed that there were no differences in haemorrhage and pathological impairment of the lungs and pulmonary bacilli burden (Fig. S21F–I). These results indicate that *A. muciniphila* affects the efficacy of BCG vaccination dependent on the function of MptpB in BCG.

**Fig. 7:**
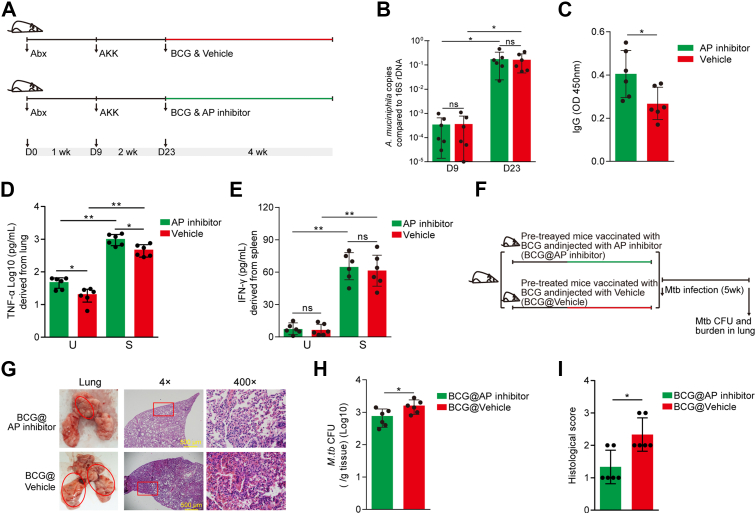
**Gut *A. muciniphila* regulates actin cytoskeleton remodelling for affecting the efficacy of BCG vaccination.** (A) Schematic representation of the influence of G-actin polymerisation into F-actin inhibition (AP inhibitor) on the function of *A. muciniphila* (AKK) to BCG. (B) *A. muciniphila* abundance in stool samples. (C) IgG concentrations in sera of mice that received BCG and AP inhibitor after 4 weeks. (D–E) Expression levels of TNF-α in lung (D) and IFN-γ in spleen (E) derived from BCG-vaccinated animals cultured with (S) or without (U) Mtb lysate stimulation. (F) Assessment of protective efficacy of BCG-vaccinated mice (BCG@Vehicle and BCG@AP inhibitor) after infection with Mtb for 5 weeks. (G) H&E staining of two typical lungs. (H–I) Numbers of intracellular Mtb CFU (H) and histological scores (I) in lung tissue. Data are presented as a mean ± SD. In vivo animal experiments included at least two biological replicates. N = 6 per group in the mouse model (A). Mann Whitney test was used to assess statistical significance for [(C), (D), (E), (H), and (I)]. Two-way ANOVA with Tukey correction for (B). ∗P < 0.05, ∗∗P < 0.01, ∗∗∗P < 0.001, and ∗∗∗∗P < 0.0001; ns, not significant.

Next, we evaluated MptpB-regulated actin cytoskeleton remodelling on the impact of *A. muciniphila* for BCG vaccination (Fig. 7A). We found that the baseline level of *A. muciniphila* was increased but with no differences between the two groups (Fig. 7B). The treatment of actin polymerisation inhibitor rather than the vehicle during BCG vaccination induced more IgG and higher levels of TNF-α, resulting in an enhanced BCG-induced vaccine response (Fig. 7C–E). The protective efficacy of these vaccinated mice against Mtb infection also showed that the BCG@AP inhibitor group had significantly reduced pulmonary haemorrhage and pathological damage and led to a lower pulmonary bacillus burden than the BCG@Vehicle group (Fig. 4F–I). In addition, there was no difference in the levels of *A. muciniphila* and palmitoleic acid between the groups before the Mtb infection, i.e. after vaccinated with BCG (Fig. S22). These data suggest that actin cytoskeleton remodelling mediates the vaccine efficacy of *A. muciniphila* on BCG.

In summary, the present findings demonstrated that higher baseline level of gut *A. muciniphila* inhibits the efficacy of BCG vaccination by producing palmitoleic acid to inactivate the function of actin cytoskeleton remodelling of MptpB in BCG.

## Discussion

Although vaccination represents the most efficient strategy for kerbing the transmission of infectious diseases, the immune responses it elicits differ markedly among individuals and across geographical populations worldwide.^5^ Variability in BCG vaccine efficacy remains a significant challenge in the global fight against TB.^3^ This study elucidates that difference in gut microbiota composition serves as a main factor that can account for variations in immunisation outcomes. By integrating animal models, multi-omics approaches, and analysis of several human datasets, we reveal that the efficacy of BCG vaccination in host is affected by the baseline levels of gut microbiota and metabolites before vaccination, highlighting gut microbiota composition and function as a determinant in shaping vaccination responses. Specifically, we discovered that higher baseline levels of *A. muciniphila* and its metabolite palmitoleic acid could significantly inhibit the BCG-elicited immune response in an MptpB-dependent manner, reducing its protective efficacy. This finding provides causal evidence and mechanistic insights into the variability in BCG vaccine efficacy and offers potential strategies to optimise BCG vaccination and improve TB prevention outcomes.

The gut–lung axis serves as an essential bridge connecting gut microbiota and pulmonary immunity.^7^^,^^8^ Our study aligns with earlier studies demonstrating that the gut microbiome is a modulator of vaccine response and immunity. Variability in cytokine expression following BCG immunisation correlates with microbiota abundance, as the modulation of gut microbiota profoundly influences vaccine response.^13^ One study shows that lactic acid bacteria diminish the viability and persistence of BCG, potently antagonise BCG, and modulate BCG-driven immune activation in human macrophages.^37^ A randomised controlled clinical trial evaluated the influence of healthy adults on the immune response to ORV finds that the immunogenicity of ORV was increased at day 7 post-vaccination in the group with modulated gut microbiota by vancomycin antibiotics treatment.^38^ Based on the findings of this study that the gut microbiota, particularly *A. muciniphila*, is involved in modulating the immune reaction towards BCG vaccination, we propose that gut *A. muciniphila* in healthy humans may affect the immunogenicity of the BCG vaccine via its metabolite palmitoleic acid. This suggests a strategy for optimising the BCG vaccine performance by editing the gut microbiota.

Commensal bacterial metabolites contribute to host-microbiota communication and influence host well-being.^39^ Our study identified palmitoleic acid, a metabolite produced by *A. muciniphila*, as a factor in modulating the efficacy of the BCG vaccine. Palmitoleic acid impaired BCG vaccine efficacy through suppression of MptpB function, suggesting that modulating specific intestinal metabolites could enhance BCG vaccine efficacy. Moreover, our previous research shows that oral treatment with palmitoleic acid produced by *A. muciniphila* strongly inhibits TB infection and survival via epigenetic suppression of tumour necrosis factor within Mtb-infected mice.^9^ Gewirtz and colleagues reported that both germ-free and antibiotic-treated mice displayed elevated rotavirus-specific antibody titres in serum and mucosa, as well as reduced viral infection, indicating that the intestinal microbiota dampens antibody responses.^40^ These findings indicate that microorganisms and metabolites with anti-TB properties should be considered with caution for optimal BCG vaccination, as they may impair vaccine efficacy. Indeed, *A. muciniphila* colonises the human gut from early life (detectable as early as 7 days) and is present in breast milk, suggesting vertical transmission from mother to infant,^41^^,^^42^ highlighting microbiota-mediated regulation of host vaccine responsiveness and its congruence with the variability in BCG immunogenicity across populations and developmental stages.

Our results confirm that the respiratory immune system is modulated by microbial ligands and metabolites originating from the gut microbiome. In addition, alterations in respiration modulate the functional state of the intestinal microbiome, leading to alterations in the abundance and metabolism of gut bacteria. Compared with normal mice without BCG vaccination, mice exhibited reduced *A. muciniphila* and palmitoleic acid levels after BCG vaccination, indicating that changes in the lungs affected the gut. Furthermore, compared with the control group treated with BCG alone, the groups pre-treated with faecal microbiota transplantation, *A. muciniphila*, and palmitoleic acid achieved successful interference in host-level before BCG vaccination, which was characterised by increased bacterial abundance and metabolite concentration. However, after BCG vaccination and vaccine training, these colonisations were significantly reduced compared with those before vaccination and were at low levels, similar to those in the control mice treated with BCG alone. These baseline differences in the gut with pre-BCG vaccination led to the subsequently observed differences in pulmonary vaccine response, whereas the establishment of vaccine immunity also induced the reshaping of gut microbiota and metabolism. Our findings are supported by previous studies showing that changes in lung tissue (i.e., receiving the BCG vaccine) cause alterations in the gut microbiota, including a reduction in *A. muciniphila* abundance.^13^^,^^14^^,^^16^ Our study provides evidence for a bidirectional gut–lung axis in which signal exchange regulates microbial–immune interactions, emphasising the importance of reciprocal communication between distant tissues and the gut in gut microbiota intervention.

Several limitations should be acknowledged. First, broad-spectrum antibiotic treatment may alter immune tone and trained immunity, potentially confounding microbiota-specific effects. Second, the study used only one mouse strain (C57BL/6) and one sex, limiting generalisability. Third, the lack of longitudinal immune profiling prevents assessment of dynamic changes in vaccine-induced immunity over time. Fourth, the link between MptpB-mediated actin remodelling and immune activation remains indirect; it may affect antigen processing/presentation via endocytosis, immunological synapse formation, or innate immune signalling, requiring further study. Future studies employing multi-strain, both sexes and longitudinal sampling will be necessary to more comprehensively elucidate the mechanisms.

Host genetic factors, food intake, and probiotic and antibiotic consumption significantly influence the composition of the gut microbiota and their metabolites,^43^, ^44^, ^45^, ^46^ indicating the complexity of optimising BCG vaccination. The combined effects of these factors may lead to regional and population differences in the response to the BCG vaccine. Therefore, developing personalised BCG vaccination strategies tailored to different genetic backgrounds and environmental factors may be useful for enhancing the global TB prevention outcomes. In summary, our data suggests that stratifying individuals according to gut microbiota composition or gut metabolites profile could serve as strategies for optimal BCG vaccine efficacy.

## Contributors

DC, LC, CC, YL, ZG planned the experiments. DC, LC, CC, YY, XW, DC, PY, XL, YZ, JL performed the experiments and analysis. JFX, JP, GZ provided the research resources. DC, LC, YL, ZG wrote the draft of the manuscript. DC, LC, CC, and ZG accessed and verified the underlying data supporting the study. All authors contributed to the edits of the manuscript. All authors read and approved the final version of the manuscript.

## Data sharing statement

Data of 16S rRNA sequencing are available in a public repository (https://dataview.ncbi.nlm.nih.gov/) with the accession number PRJNA1191378. The proteome microarray data will be available at the OMIX database (https://ngdc.cncb.ac.cn/omix/) with the accession number OMIX016042. The proteomics data are available at the ProteomeXchange Consortium (http://proteomecentral.proteomexchange.org) via the iProX partner repository with the dataset identifier PXD076732. All study data are included in the article and/or supporting information. Further enquiries should be directed to the corresponding author (gezhh7@mail.sysu.edu.cn).

## Declaration of interests

The authors declare that they have no competing interests.

## Appendix A Supplementary data

Supplementary Figures and Tables

Western Blots
