# Supplementary material for: Investigating the role of baseline gut Akkermansia muciniphila and its co-metabolite palmitoleic acid in BCG vaccine efficacy: a preclinical study
Source: eBioMedicine. 2026 Jul 23;130:106404. doi: 10.1016/j.ebiom.2026.106404 (PMC13427514; doi:10.1016/j.ebiom.2026.106404)
Supplement: Supplementary Figures and Tables [file mmc1.pdf]

Supplementary information

Investigating the role of baseline gut *Akkermansia muciniphila* and its co-metabolite in BCG vaccine efficacy: a preclinical study

Dongni Chen[#], Lingming Chen[#], Chun Chen[#], Yongen Yan, Xiaoyu Wu, Dongli Chen, Peibo Yuan, Xiaoxuan Long, Yi Zou, Jie Lin, Jun-Fa Xu, Jiang Pi, Gucheng Zeng, Yongjun Lu, Zhenhuang Ge[*]

Supplementary figures S1 to S22 (page 2 to page 25)

Supplementary tables S1 to S5 (page 26 to page 39)

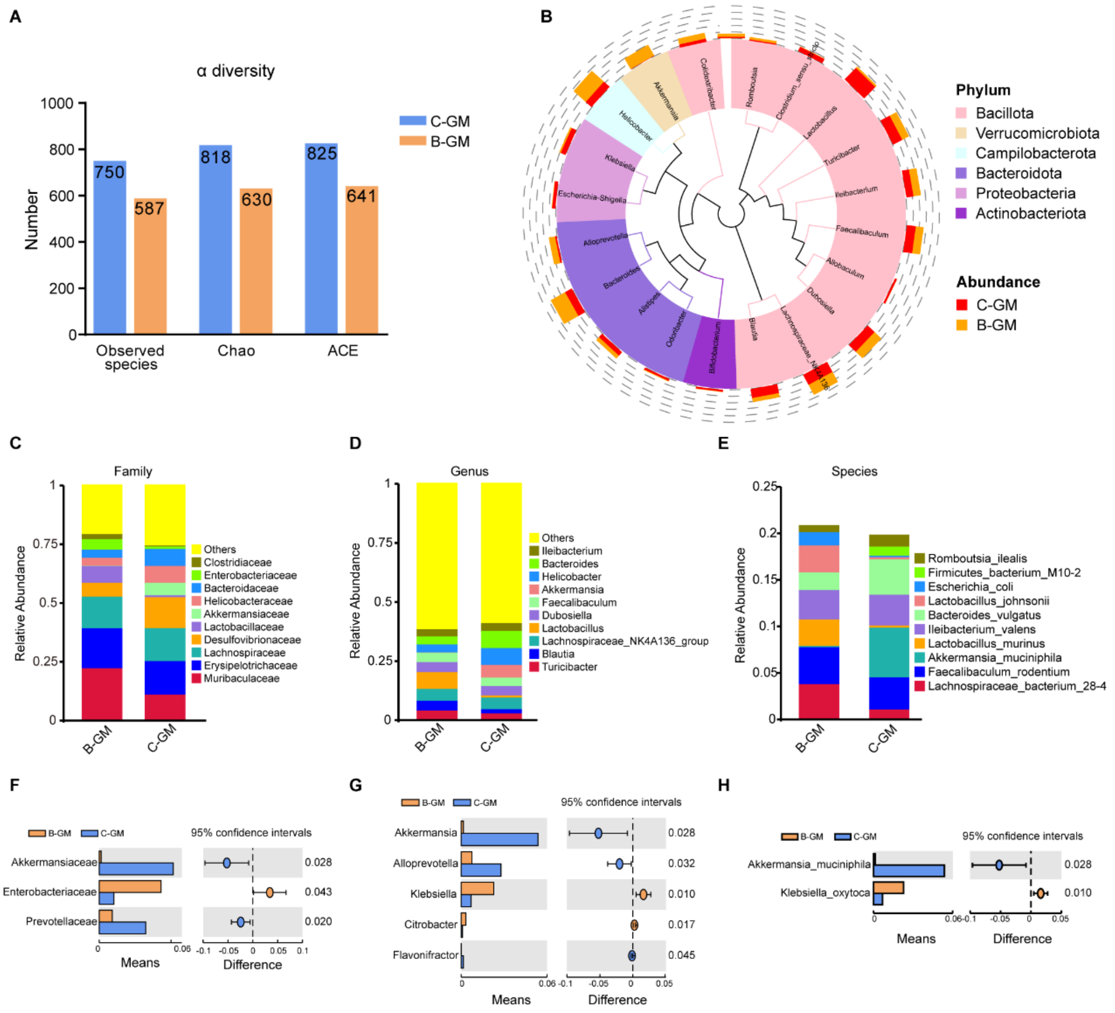

Figure S1. Gut microbiota profiles of mice before BCG vaccination. (A) Alpha diversity of microbiota. (B) The top 20 evolutionary trees at the genus level (C-E) Relative abundance of gut bacteria in the faecal samples at family (C), genus (D), and species (E) levels. (F-H) Bacteria with significant differences at family (F), genus (G), and species (H) levels. Data are presented as a mean ± SD. In vivo animal experiments included at least two biological replicates. N = 6 per group in the mouse model. Mann Whitney test was used to assess statistical significance *P < 0.05, **P < 0.01, ***P < 0.001, and ****P < 0.0001; ns, not significant.

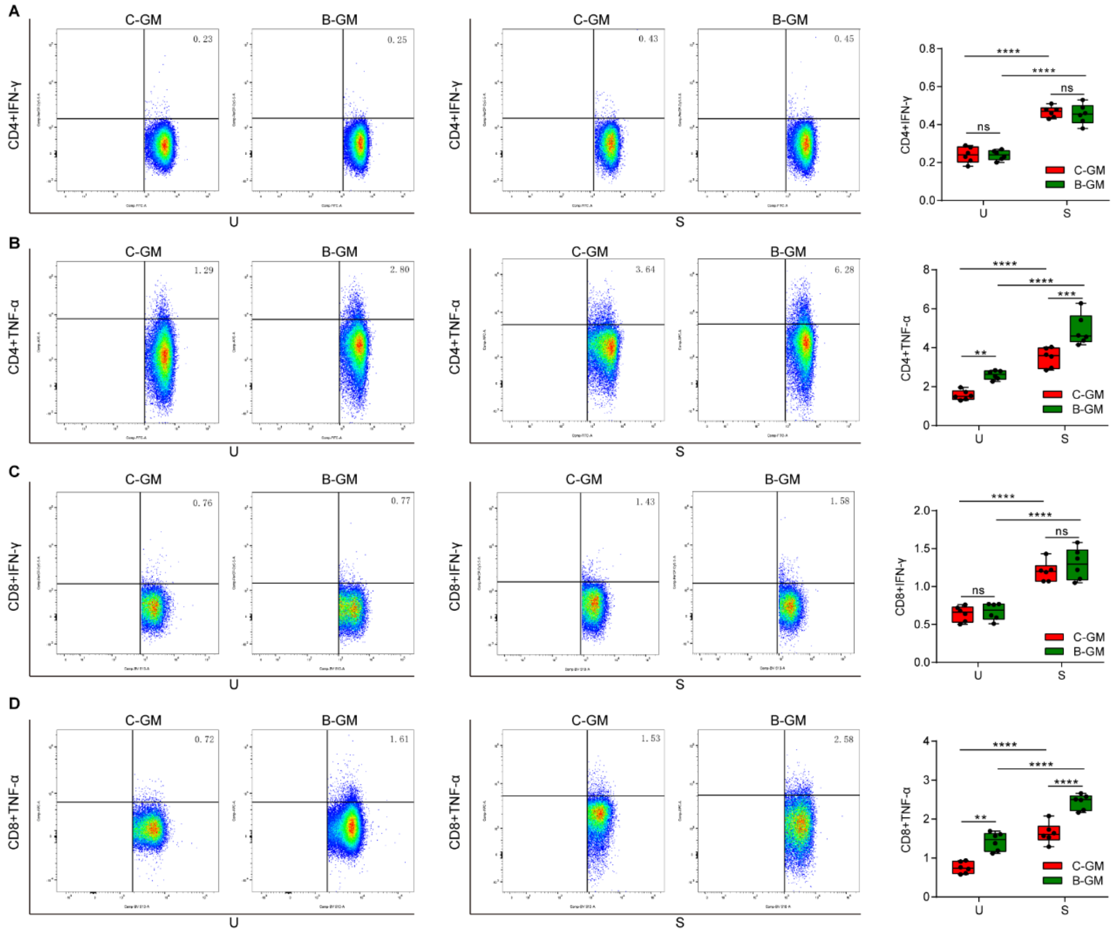

Figure S2. Baseline gut microbiota composition influences CD4⁺ and CD8⁺ T cell responses following BCG vaccination. Mice were pre-treated with gut microbiota from vaccination-naive (C-GM) or vaccination-adapted (B-GM) donors and then vaccinated with BCG. Four weeks after vaccination, cells were isolated from the lungs and spleens, cultured either without (U) or with (S) Mtb lysate stimulation, and then analysed by flow cytometry. Panels show the expression of $CD4^+$ IFN-γ (A), $CD4^+$ TNF-α (B), $CD8^+$ IFN-γ (C), and $CD8^+$ TNF-α (D) cells in the lung and spleen. Box-and-whisker plots show the center line as the median and the box limits as the first and third quartiles (n = 6 mice per group). Statistical comparisons were performed using two-way ANOVA with Tukey's correction; *P < 0.05, **P < 0.01, ***P < 0.001, ****P < 0.0001; ns, not significant.

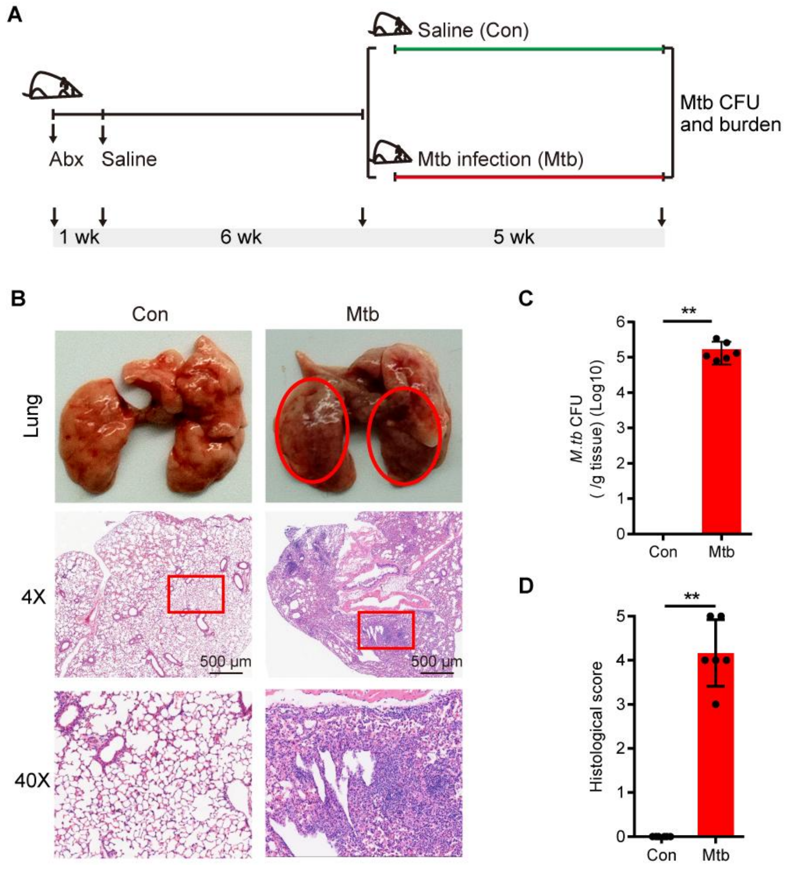

Figure S3. Histopathological assessment of naive (non-BCG-vaccinated) mice following Mtb infection. (A) Assessment of sham-immunised mice infected with Mtb for 5 weeks. (B) H&E staining of two typical lungs. (C) Numbers of intracellular Mtb CFU in lung tissue. (D) Histological scores of lung tissue.

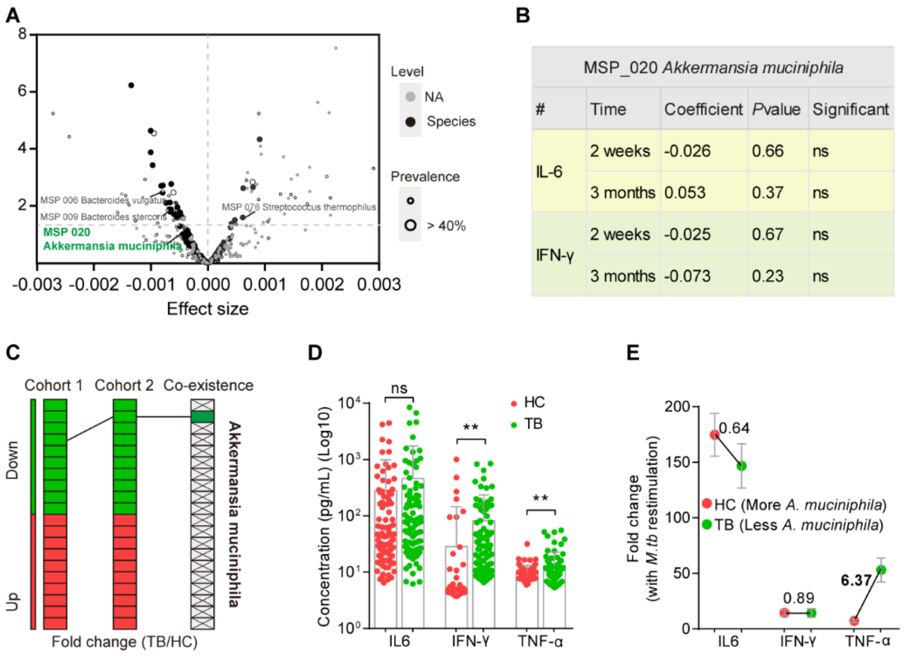

Figure S4. The immune response of human subjects infected with Mtb and treated with BCG vaccine. (A) Significant species associated with trained immune responses subject to $P < 0.05$ and prevalence > 40%. (n = 321 healthy human participants received BCG vaccine, as previously reported cohort)[13]. (B) The abundance of *A. muciniphila* is not significantly correlated with the response phenotypes of IL-6 and IFN-γ. (C) The top ten species enriched in HC or individuals with TB from two cohorts (Cohort 1, HC = 28, TB = 26; Cohort 2, HC = 17, TB = 19) as we previously reported[9]. (D) Expression levels of TNF-α, IFN-γ, and IL-6 in the culture supernatants of PBMCs derived from participants with TB (n = 96) and HC (n = 96). (E) Changes in TNF-α, IFN-γ, and IL-6 levels in cultured supernatants of PBMCs after ex vivo re-stimulation with Mtb lysates as we previously reported[9]. $^*P < 0.05$; ns indicates no significance.

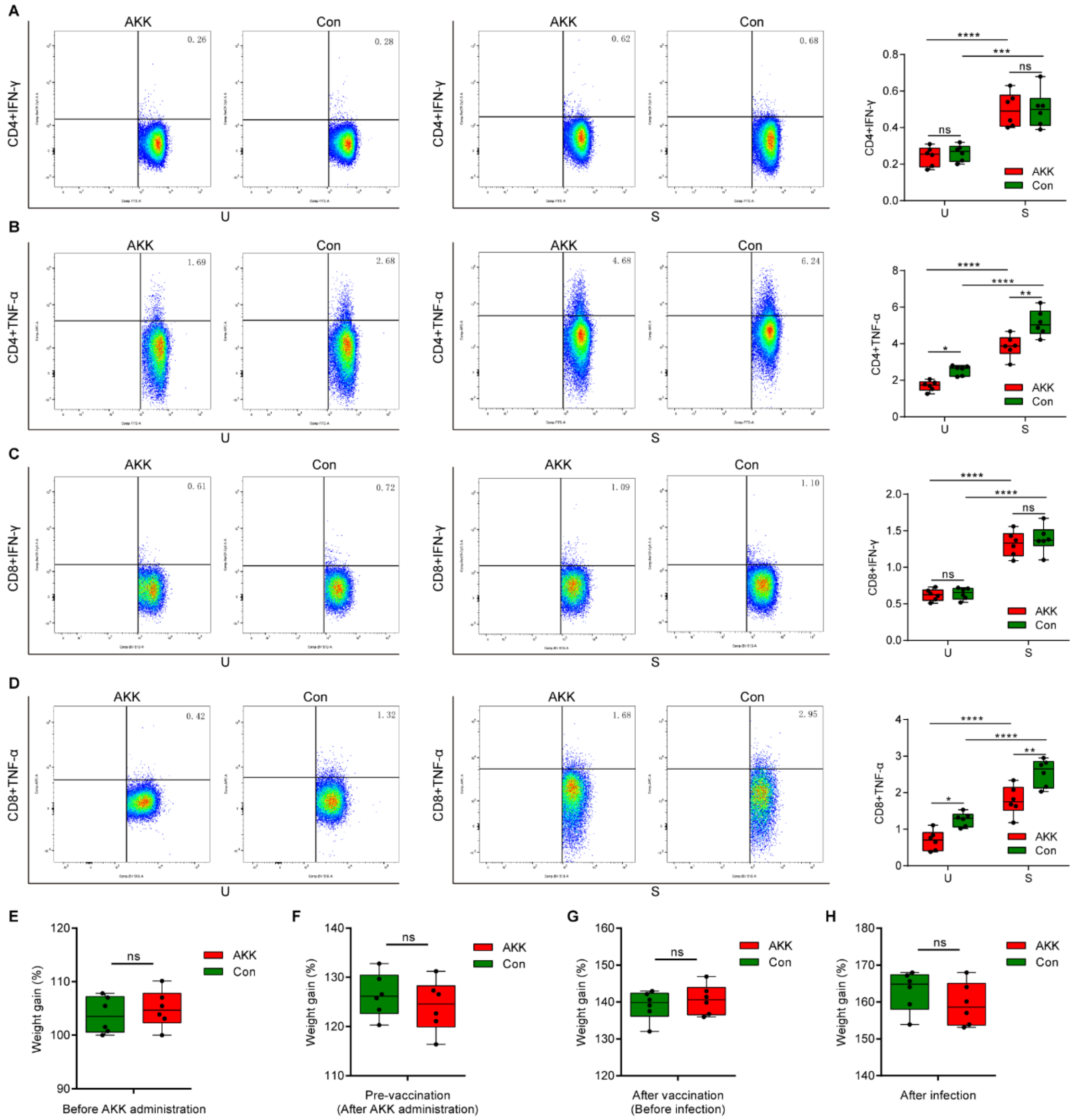

Figure S5. The abundance of *A. muciniphila* before vaccination mediates CD4$^+$ and CD8$^+$ T cell responses following BCG vaccination. Mice were pre-treated with *A. muciniphila* (AKK) or saline as control (Con) and then vaccinated with BCG. Four weeks after vaccination, cells were isolated from the lungs and spleens, cultured either without (U) or with (S) Mtb lysate stimulation, and then analysed by flow cytometry. Panels show the expression of CD4$^+$ IFN-γ (A), CD4$^+$ TNF-α (B), CD8$^+$ IFN-γ (C), and CD8$^+$ TNF-α (D) cells in the lung and spleen. (E-F) Body weight changes at different stages throughout the experiment, before AKK administration (E), Pre-vaccination (After AKK administration) (F), After vaccination (Before infection) (G), After

infection (H). Box-and-whisker plots show the center line as the median and the box limits as the first and third quartiles (n = 6 mice per group). Statistical comparisons were performed using two-way ANOVA with Tukey's correction for [(A), (B), (C), (D)]. Mann Whitney test was used to assess statistical significance for [(E), (F), (G), (H)]. $^{*}P < 0.05$, $^{**}P < 0.01$, $^{***}P < 0.001$, $^{****}P < 0.0001$; ns, not significant.

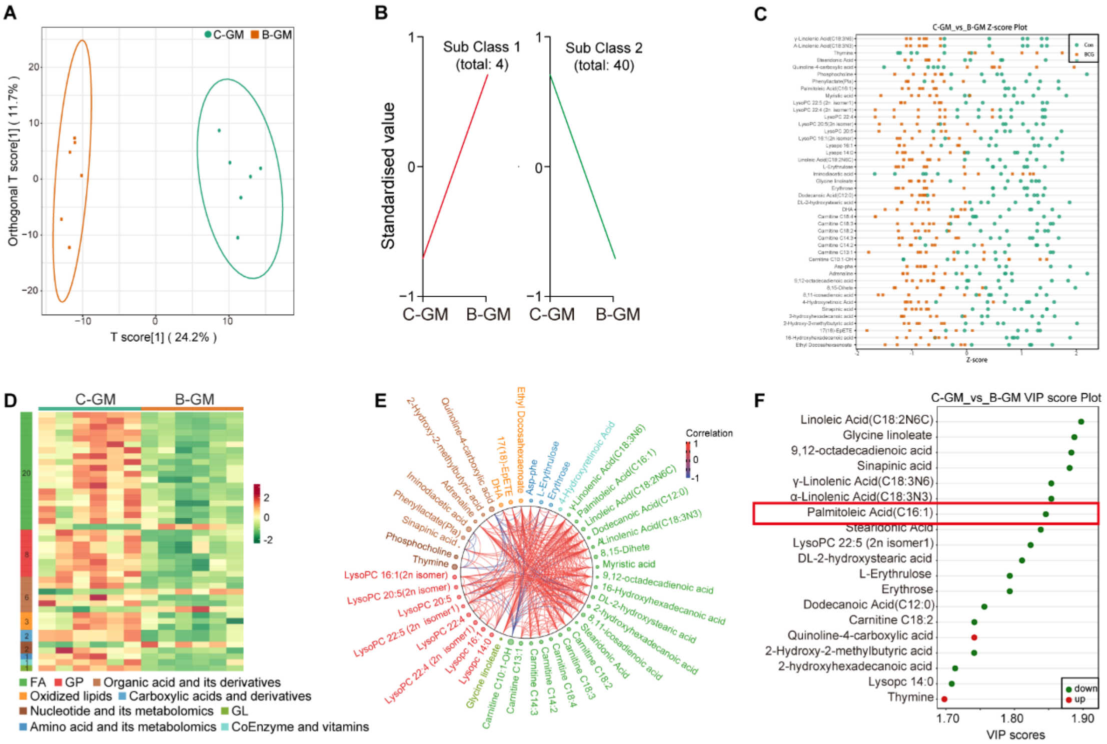

Figure S6. Metabolic profiles in the sera of mice before BCG vaccination. (A) OPLS-DA plot of serum metabolites from C-GM and B-GM transplanted mice. (B) K-means analysis of content change trends of metabolites in different samples. (C) Z-scores of differentially expressed metabolites in different samples. (D) Heatmap showing differentially enriched metabolites in sera between the C-GM and B-GM groups. (E) Correlation between the identified metabolites. The red line indicates a positive correlation, whereas the blue line indicates a negative one. (F) The VIP score of the differential metabolites reveals their potentially important role.

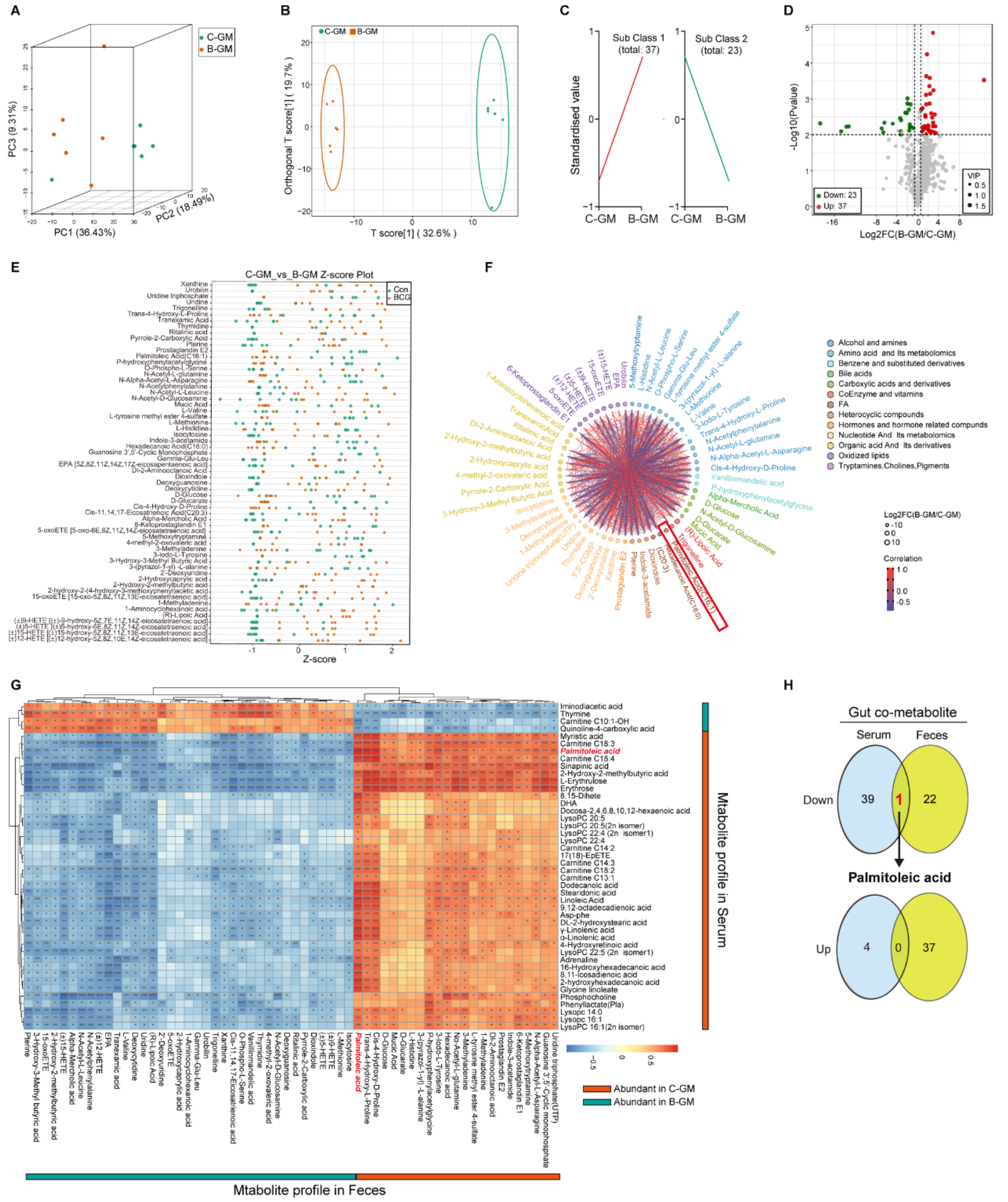

Figure S7. Metabolic profiles in the faeces of mice before BCG vaccination. (A) PCA plot of faecal metabolites from C-GM and B-GM transplanted mice. (B) OPLS-DA plot of faecal metabolites from C-GM and B-GM mice (BCG). (C) K-means analysis of content change trends of metabolites in different samples. (D) Volcano plot of all metabolites found in faecal samples. The red and green points indicate significant differential metabolites with a variable importance in projection (VIP) score > 1, P < 0.01, and FC > 1.5. (E) Z-scores of differentially expressed metabolites in different

samples. (F) Correlation between the identified metabolites. (G) Heatmap showing positive (red) and negative (blue) correlations between faecal (X axis) and serum (Y axis) metabolites measured in B-GM mice. *$P < 0.05$, **$P < 0.01$, and ***$P < 0.001$ determined by the Spearman correlation. (H) Venn diagram showing the only gut co-metabolite palmitoleic acid in sera directly associated with faecal concentration.

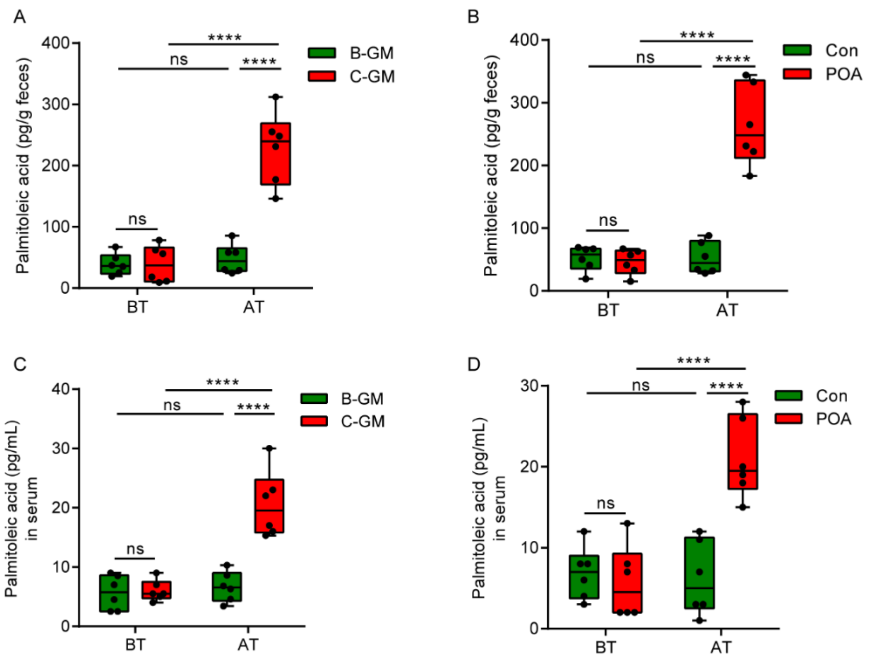

Figure S8. Palmitoleic acid concentrations in faeces and serum of mice before and after microbiota transplantation or palmitoleic acid treatment. Antibiotic-pretreated mice were subjected to either treatment with faecal microbiota transplantation (C-GM or B-GM) or palmitoleic acid (POA) for two weeks. Faecal and serum samples were collected before (BT) and two weeks after (AT) the respective treatments. Panels show POA concentrations in faeces and serum, respectively. Of note, the POA concentrations in treated mice remained within the normal physiological range observed in untreated mice, indicating that the treatment induced differences between groups while maintaining physiological relevance. Box-and-whisker plots show the center line as the median and the box limits as the first and third quartiles (n = 6 mice per group). Statistical comparisons were performed using two-way ANOVA with Tukey's correction; *$P < 0.05$, **$P < 0.01$, ***$P < 0.001$, ****$P < 0.0001$; ns, not significant.

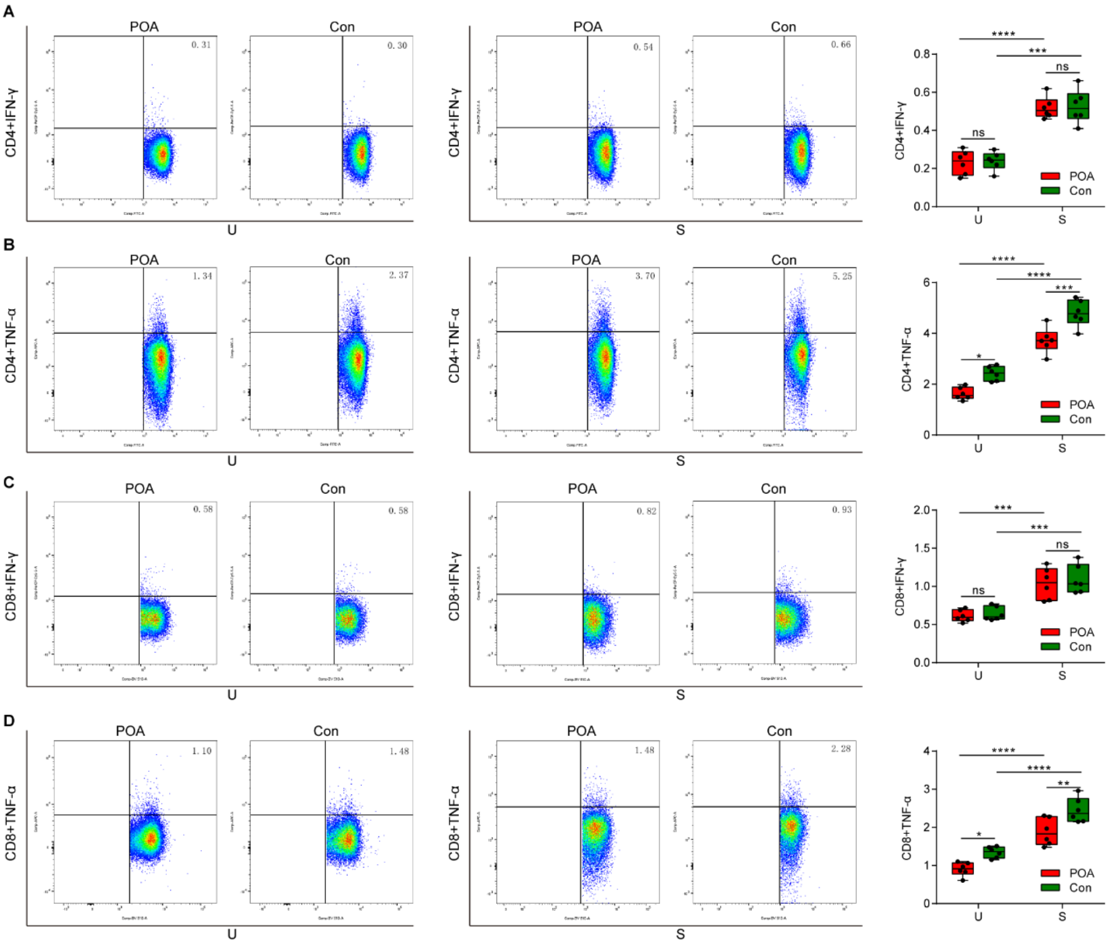

Figure S9. Treatment with palmitoleic acid before vaccination mediates CD4⁺ and CD8⁺ T cell responses following BCG vaccination. Mice were pre-treated with palmitoleic acid (POA) or saline as control (Con) and then vaccinated with BCG. Four weeks after vaccination, cells were isolated from the lungs and spleens, cultured either without (U) or with (S) Mtb lysate stimulation, and then analysed by flow cytometry. Panels show the expression of $CD4^+$ IFN-γ (A), $CD4^+$ TNF-α (B), $CD8^+$ IFN-γ (C), and $CD8^+$ TNF-α (D) cells in the lung and spleen. Box-and-whisker plots show the center line as the median and the box limits as the first and third quartiles (n = 6 mice per group). Statistical comparisons were performed using two-way ANOVA with Tukey's correction; *$P < 0.05$, **$P < 0.01$, ***$P < 0.001$, ****$P < 0.0001$; ns, not significant.

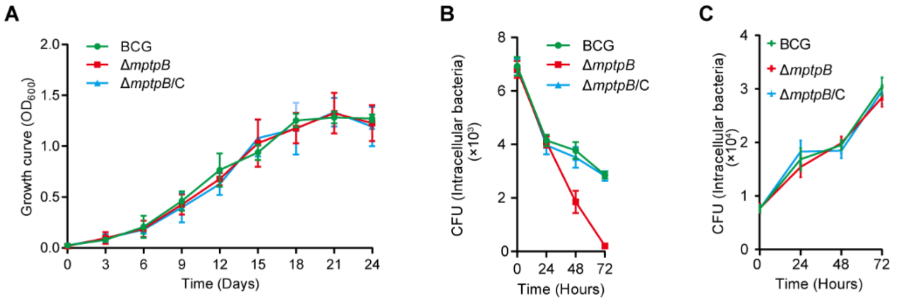

Figure S10. MptpB promotes the survival of mycobacteria in macrophages. (A) The in vitro growth curve of the wild-type (BCG), *mptpB* mutant (Δ*mptpB*), and complementation (Δ*mptpB/C*) BCG strains. (B-C) Survival of different BCG strains in activated J774A.1 macrophages (B) and resting macrophages (C).

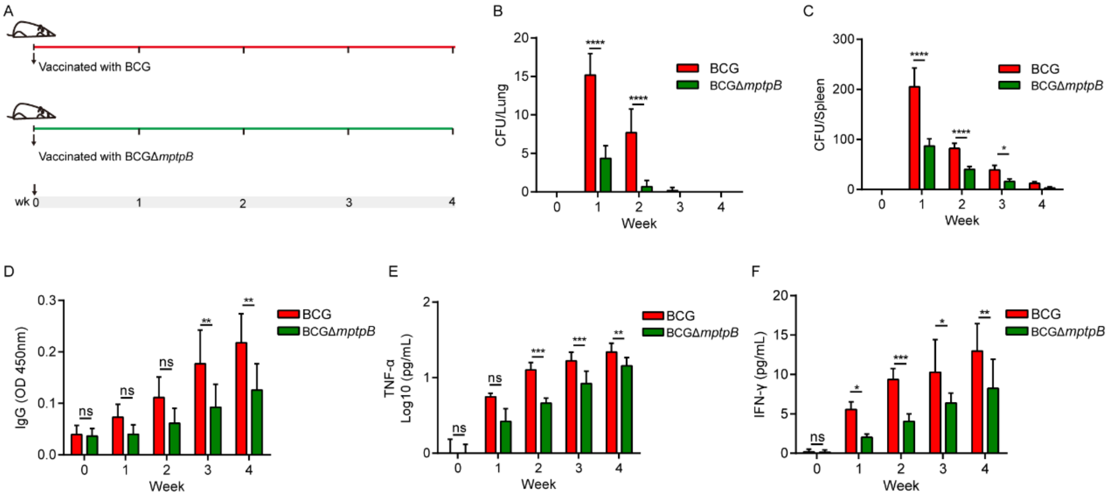

Figure S11. Early intracellular survival of BCG is required for effective immune priming. Mice were subcutaneously vaccinated with either BCG or BCGΔ*mptpB* (A). At weeks 1, 2, 3, and 4 post-vaccination, bacterial loads (CFU) in the lungs (B) and spleen (C), serum IgG levels (D), pulmonary TNF-α (E), and splenic IFN-γ (F) were measured. The graphs show that BCGΔ*mptpB*-vaccinated mice had significantly lower live bacterial counts in both lungs and spleen at indicated time points compared to BCG-vaccinated mice, accompanied by reduced serum IgG, pulmonary TNF-α, and splenic IFN-γ responses. Data are presented as mean ± SD (n = 6 mice per group). Statistical comparisons were performed using two-way ANOVA with Tukey's correction; *P < 0.05, **P < 0.01, ***P < 0.001, ****P < 0.0001.

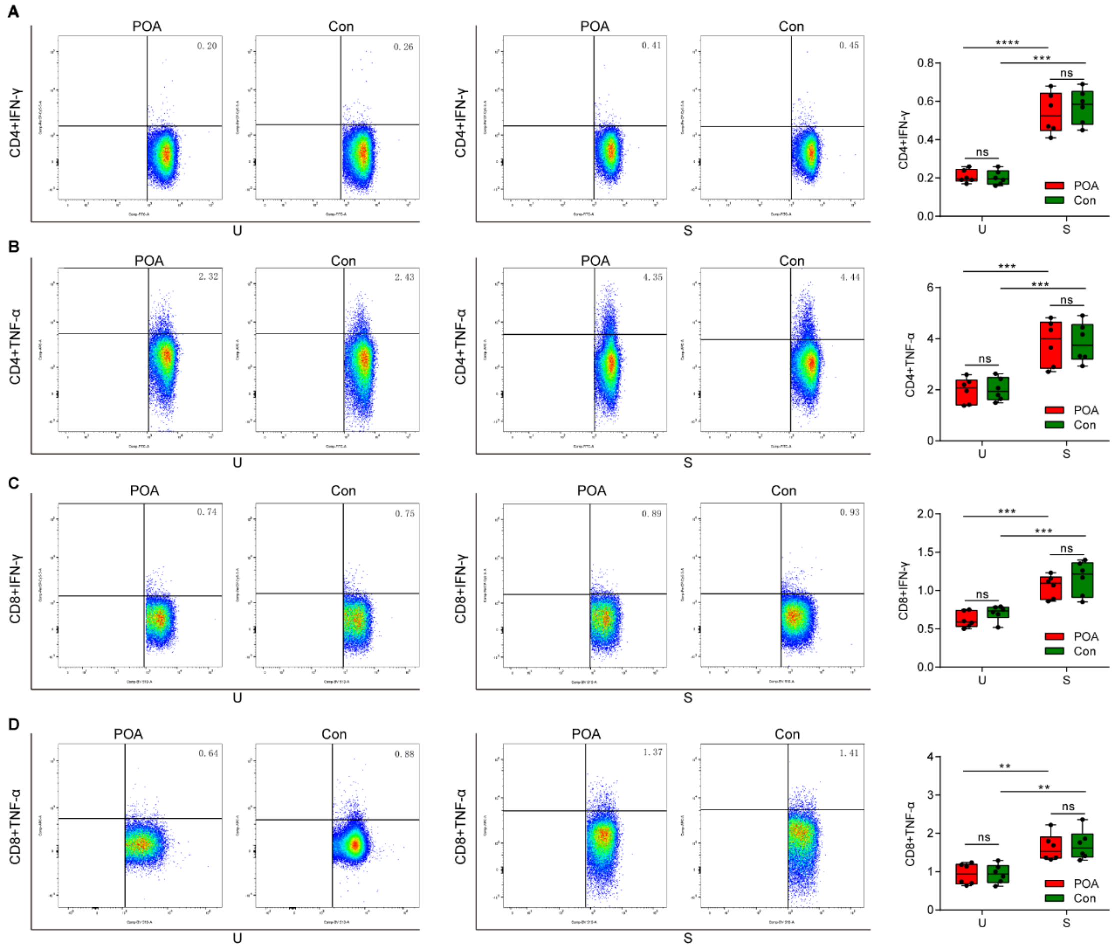

Figure S12. Palmitoleic acid pre-treatment before BCGΔ*mptpB* vaccination does not alter CD4⁺/CD8⁺ T cell responses. Mice were pre-treated with palmitoleic acid (POA) or saline as control (Con) and then vaccinated with BCGΔ*mptpB*. Four weeks after vaccination, cells were isolated from the lungs and spleens, cultured either without (U) or with (S) Mtb lysate stimulation, and then analysed by flow cytometry. Panels show the expression of $CD4^+$ IFN-γ (A), $CD4^+$ TNF-α (B), $CD8^+$ IFN-γ (C), and $CD8^+$ TNF-α (D) cells in the lung and spleen. Box-and-whisker plots show the center line as the median and the box limits as the first and third quartiles (n = 6 mice per group). Statistical comparisons were performed using two-way ANOVA with Tukey's correction; *P < 0.05, **P < 0.01, ***P < 0.001, ****P < 0.0001; ns, not significant.

Protein	Metabolite Log2FC / P-value	Docking	Score
MptpB	Thymine (1.21 / 0.0013**)	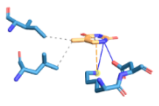	1.22 Low
	Myristic acid (-1.54 / 0.0019**)	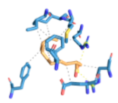	3.95 Low
	Dodecanoic Acid (-1.82 / 0.0043**)	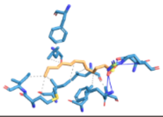	6.69 High
	α-Linolenic Acid (-1.41 / 0.0016**)	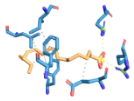	5.23 Medium
	γ-Linolenic Acid (-1.41 / 0.0016**)	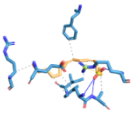	7.40 High
	Linoleic Acid (-0.78 / 0.00075***)	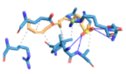	7.38 High
	Palmitoleic Acid (-1.37 / 0.00093***)	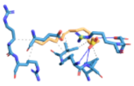	6.90 High
	4-Hydroxyretinoic Acid (-1.22 / 0.0047**)	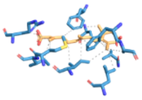	5.84 Medium
	Sinapinic Acid (-2.39 / 0.00033***)	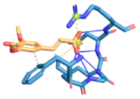	5.82 Medium
	Phosphocholine (-0.78 / 0.0055**)	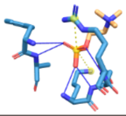	6.49 High

Figure S13. Docking analysis reveals an interaction between MptpB and the enriched serum metabolites. Binding modes of metabolites to MptpB protein using DeepMice tool. The foldchange (BCG-vaccinated mice versus control mice) and VIP values of the metabolites are shown below. The green dots indicate the significance of the metabolites in both groups, with larger ones being more important. Metabolites are shown in the ball-and-stick model in yellow, and the polar hydrogen bonds formed between MptpB and metabolites are shown as dashed lines. Metabolites with higher scores indicated a greater affinity for MptpB.

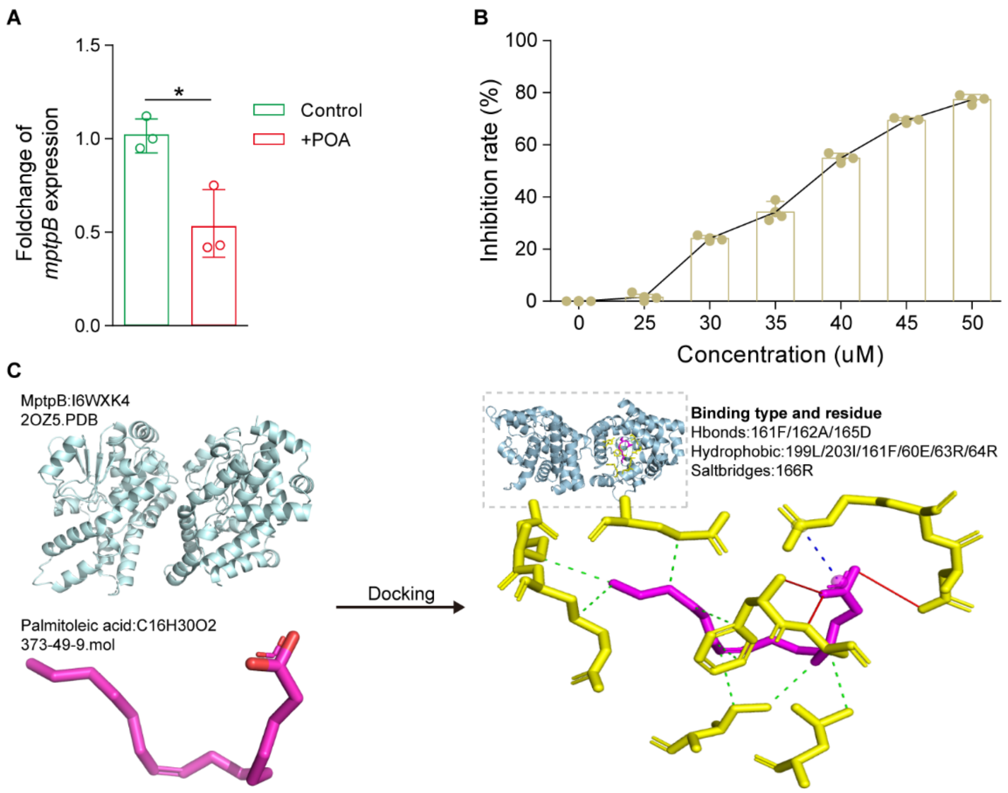

Figure S14. Palmitoleic acid inhibits MptpB expression and enzymatic activity. (A) Quantitative PCR analysis of *mptpB* mRNA expression in BCG cells treated with palmitoleic acid (POA) or saline (Con). Data are presented as the mean ± SD and represent three biological replicates. **P* < 0.05, Mann Whitney test. (B) Measurement of the dose-dependent inhibitory effect of palmitoleic acid on MptpB enzyme activity. (C) Details of palmitoleic acid binding to MptpB. Metabolites are shown in pink in the ball-and-stick model, and the type and number of bonds formed between MptpB and palmitoleic acid are shown.

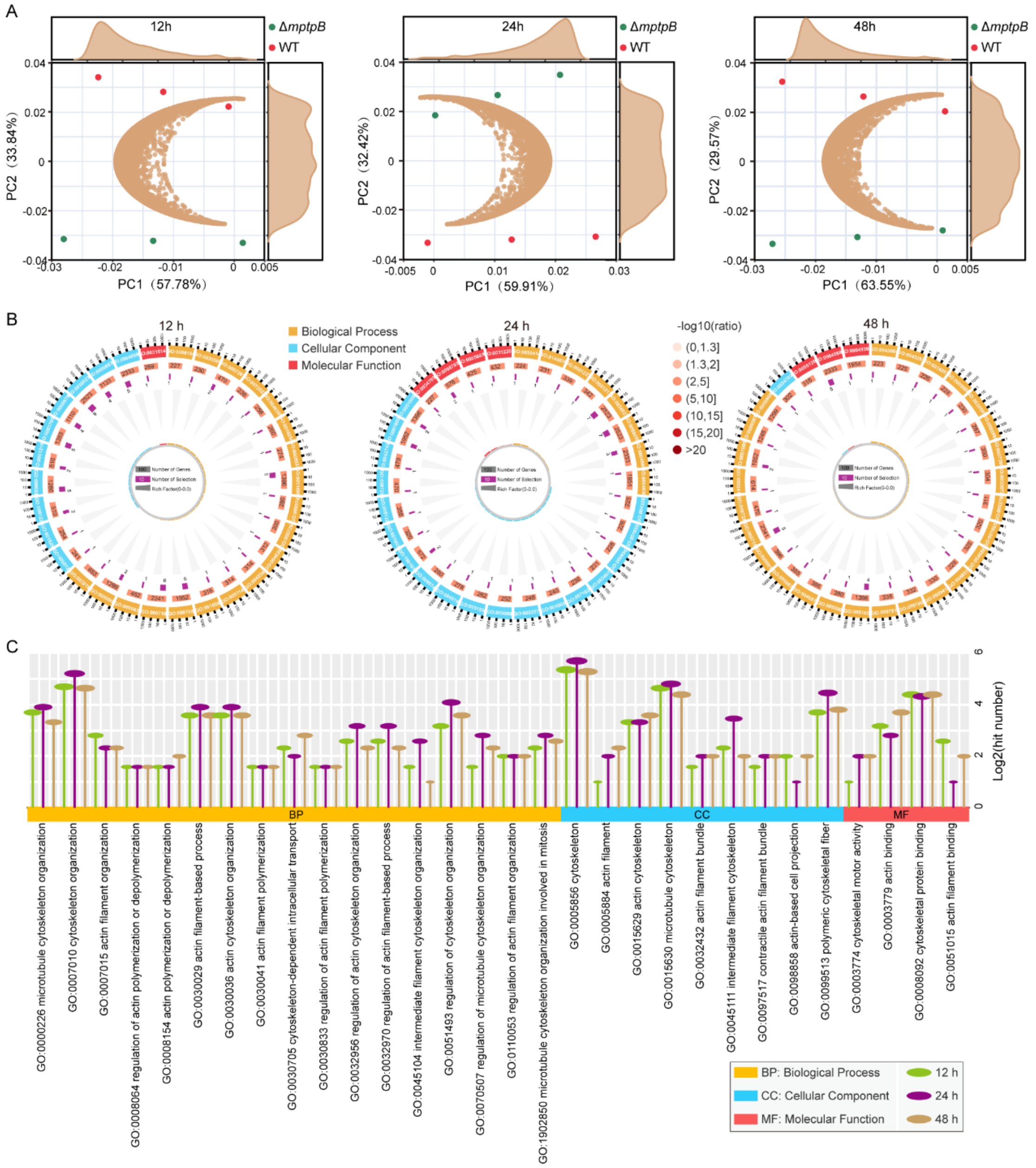

Figure S15. Proteomic profiles of macrophages infected with BCG or BCGΔ*mptpB*. (A) PCA plots showing significant differences in protein expression profiles between cells infected with *mptpB* knockout BCG strain (Δ*mptpB*) and wild-type BCG strain (WT) in the early stage at 12, 24, and 48h. (B) GO enrichment analysis of the MptpB-regulated proteins identified at indicated time points. (C) MptpB-regulated proteins are enriched in numerous actin cytoskeleton-related pathways at all three time points.

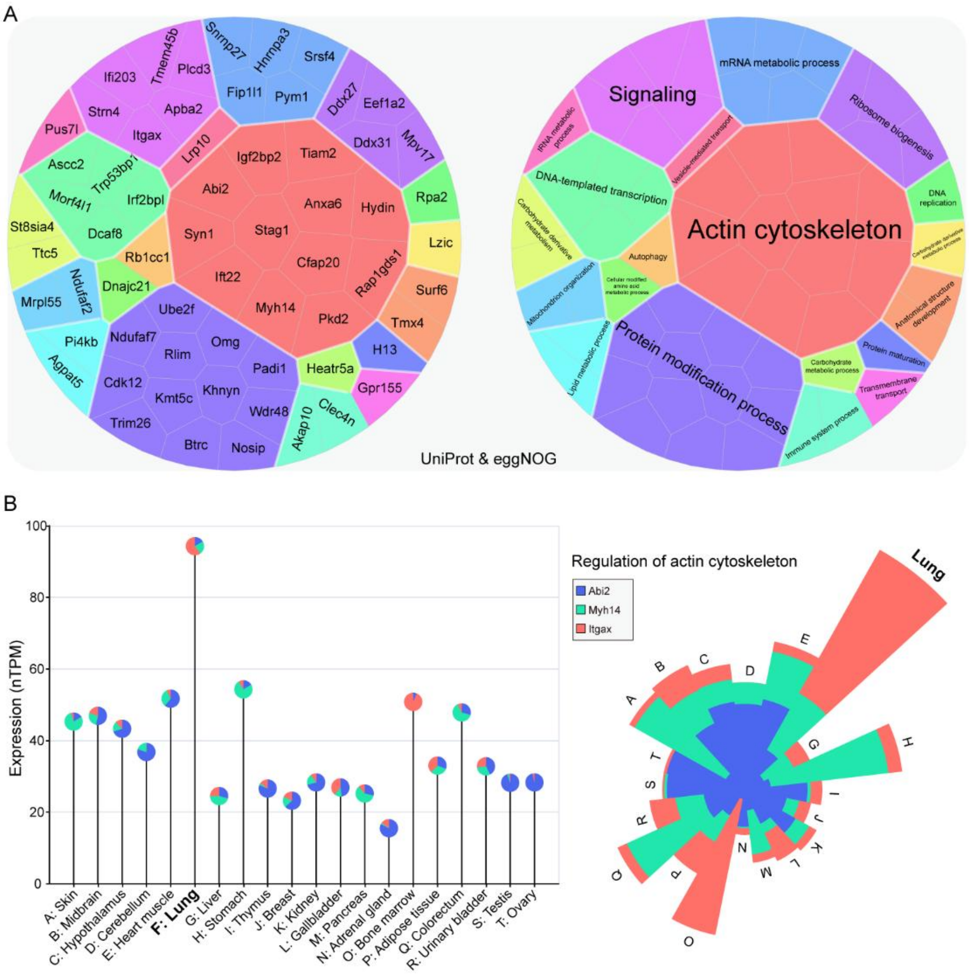

Figure S16. The Voronoi treemap visualises 63 proteins regulated by MptpB at all three time points. (A) The Voronoi treemap visualises hierarchically organised information on 63 MptpB-regulated proteins in the early stages of BCG vaccine infected macrophages. Proteins were clustered according to their functional categories. (B) Proteins associated with the regulation of the actin cytoskeleton reported in the Human Protein Atlas database (https://www.proteinatlas.org/) regarding their expression levels across various human tissues. The actin cytoskeleton-associated proteins Abi2, Myh14, and Itgax are highly expressed in human lung tissue.

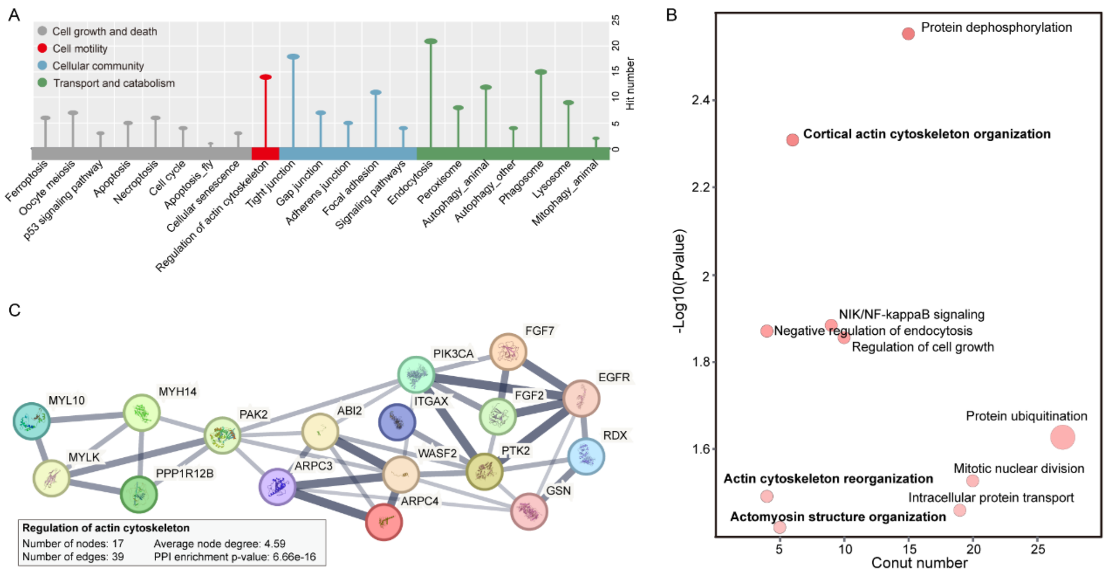

Figure S17. MptpB-regulated proteins are related to the functional category of the actin cytoskeleton. (A) Functional enrichment analysis of the MptpB-interacting proteins in HuProt™ human proteome microarray showed these proteins were also clustered in the actin cytoskeleton regulation pathway. (B) Functional enrichment analysis of the molecular functions of proteins identified in HuProt™ revealed that the actin cytoskeleton pathways were mostly significantly enriched. (C) Integrated network analysis of the actin cytoskeleton-associated proteins regulated by MptpB in macrophage proteome data and HuProt™ human proteome microarray. An enrichment analysis was performed to extract the significant subnetworks of a complex network. The thick edges indicate a required interaction score of at least 0.7, and the thin edges indicate a required interaction score of at least 0.4. The edges with high values ranged from thin to thick.

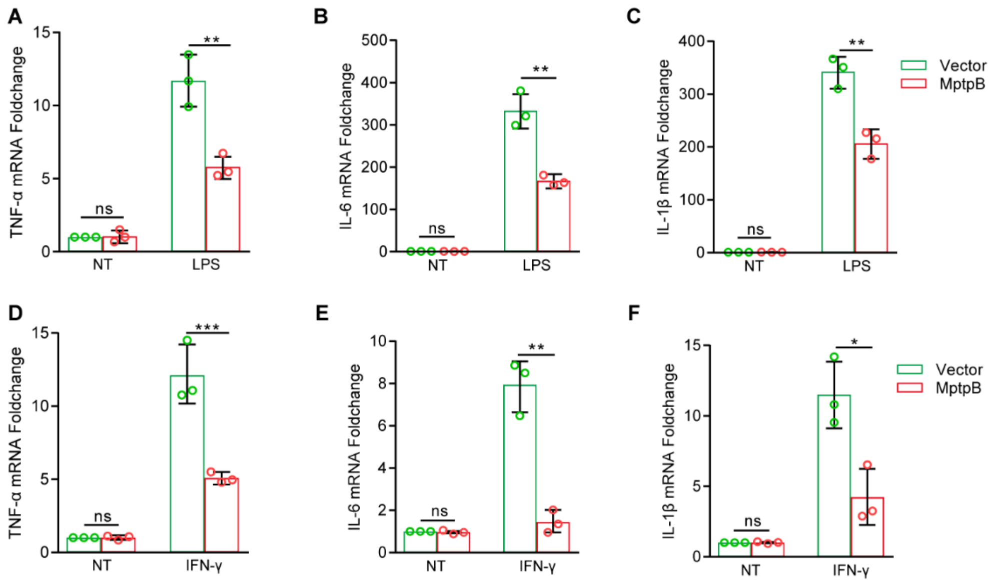

Figure S18. MptpB inhibits the activation of the NF-κB pathway in host cells, manifested as a reduction in cytokines. (A-C) Quantitative PCR analysis of TNF-α (A), IL-6 (B), and IL-1β (C) mRNA in Raw264.7 macrophages transfected with MptpB and stimulated with LPS. (D-F) Quantitative PCR analysis of TNF-α (D), IL-6 (E), and IL-1β (F) mRNA levels in macrophages transfected with MptpB and stimulated with IFN-γ. Data are representative of three biological replicates. Data are presented as mean ± SD. Mann Whitney test was used to assess statistical significance. *$P < 0.05$, **$P < 0.01$, and ***$P < 0.001$; ns, not significant.

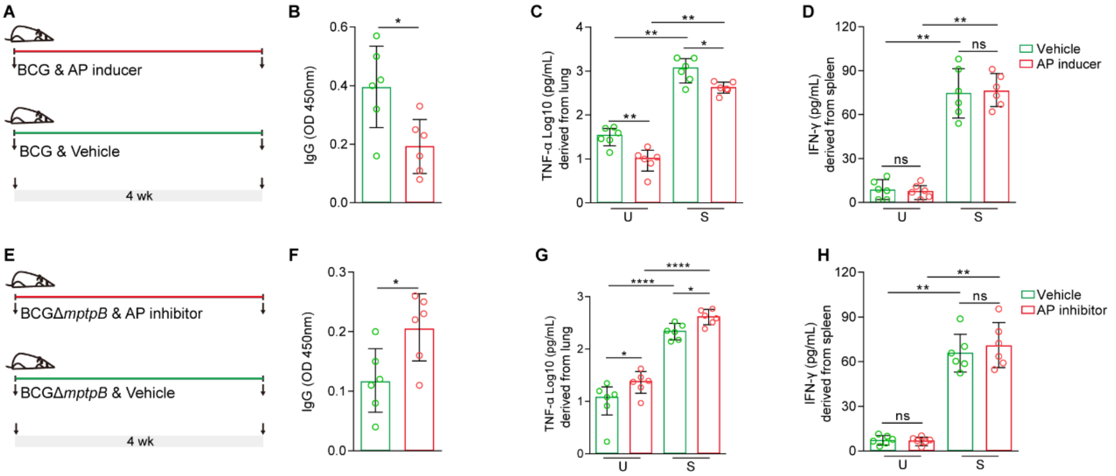

Figure S19. Regulation of the actin cytoskeleton pathway is activated by MptpB to mediate BCG-induced immunity. (A) Schematic diagram of the effect of actin polymerization induction on the function of BCG vaccine. Jasplakinolide (JasP) is an inducer of actin polymerization. (B) IgG concentrations in sera of mice that received BCG and actin polymerization inducer after 4 weeks. (C-D) Expression levels of TNF-α in lung (C) and IFN-γ in spleen (D) derived from BCG-vaccinated animals cultured with (S) or without (U) Mtb lysate stimulation. (E) Schematic diagram of the effect of actin polymerization inhibition on the function of BCG vaccine. Cytochalasin D (CytoD) is an inhibitor of actin polymerization. (F) IgG concentrations in sera of mice that received BCG and actin polymerization inhibitor after 4 weeks. (G-H) Expression levels of TNF-α in lung (G) and IFN-γ in spleen (H) derived from BCG-vaccinated animals cultured with (S) or without (U) Mtb lysate stimulation. Data are presented as mean ± SD. In vivo animal experiments included at least two biological replicates. N = 6 per group in the mouse model. Mann Whitney test was used to assess statistical significance. *P < 0.05, **P < 0.01, ***P < 0.001, and ****P < 0.0001; ns, not significant.

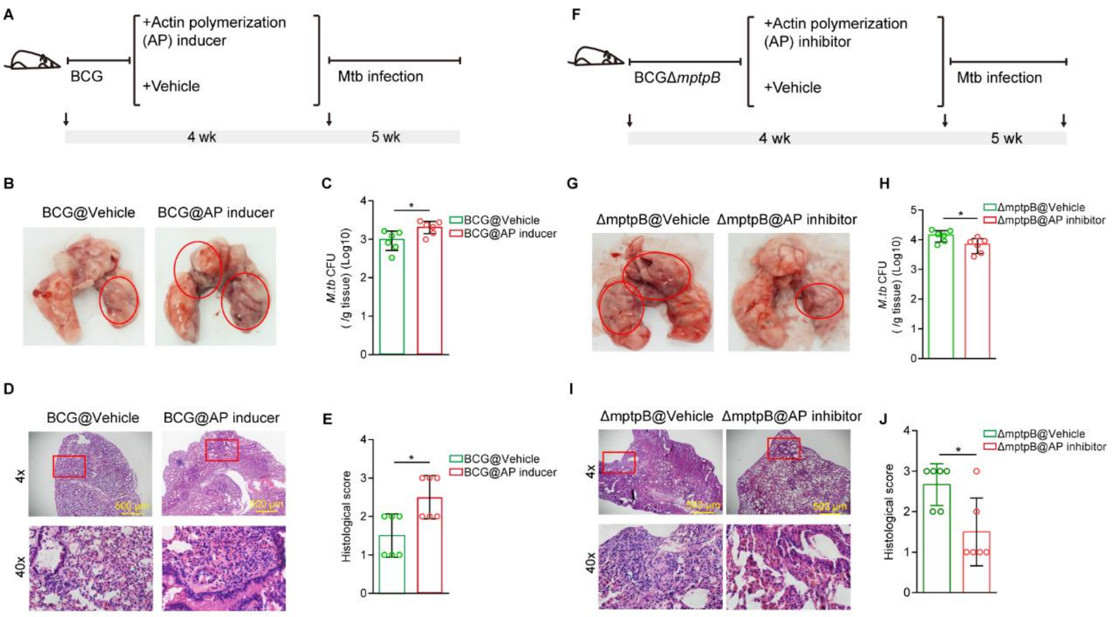

Figure S20. Actin cytoskeleton pathway regulated by MptpB affects the protective efficacy of BCG. (A) Assessment of the effect of induction of actin polymerization on the protective efficacy of BCG in mouse model of Mtb infection for 5 weeks. Jasplakinolide (JasP) is an inducer of actin polymerization. (B) Two lungs representative of Mtb-infected mice. (C) Numbers of intracellular Mtb CFU. (D) H&E staining of two representative lungs. (E) Histological scores of the lung tissue of Mtb-infected mice. (F) Assessment of the effect of inhibition of actin polymerization on the protective efficacy of BCG in mouse model of Mtb infection for 5 weeks. Cytochalasin D (CytoD) is an inhibitor of actin polymerization. (G) Two lungs representative of Mtb-infected mice. (H) Numbers of intracellular Mtb CFU. (I) H&E staining of two representative lungs. (J) Histological scores of the lung tissue of Mtb-infected mice. Data are presented as mean ± SD. In vivo animal experiments included at least two biological replicates. N = 6 per group in the mouse model. Mann Whitney test was used to assess statistical significance. *P < 0.05, **P < 0.01, ***P < 0.001, and ****P < 0.0001; ns, not significant.

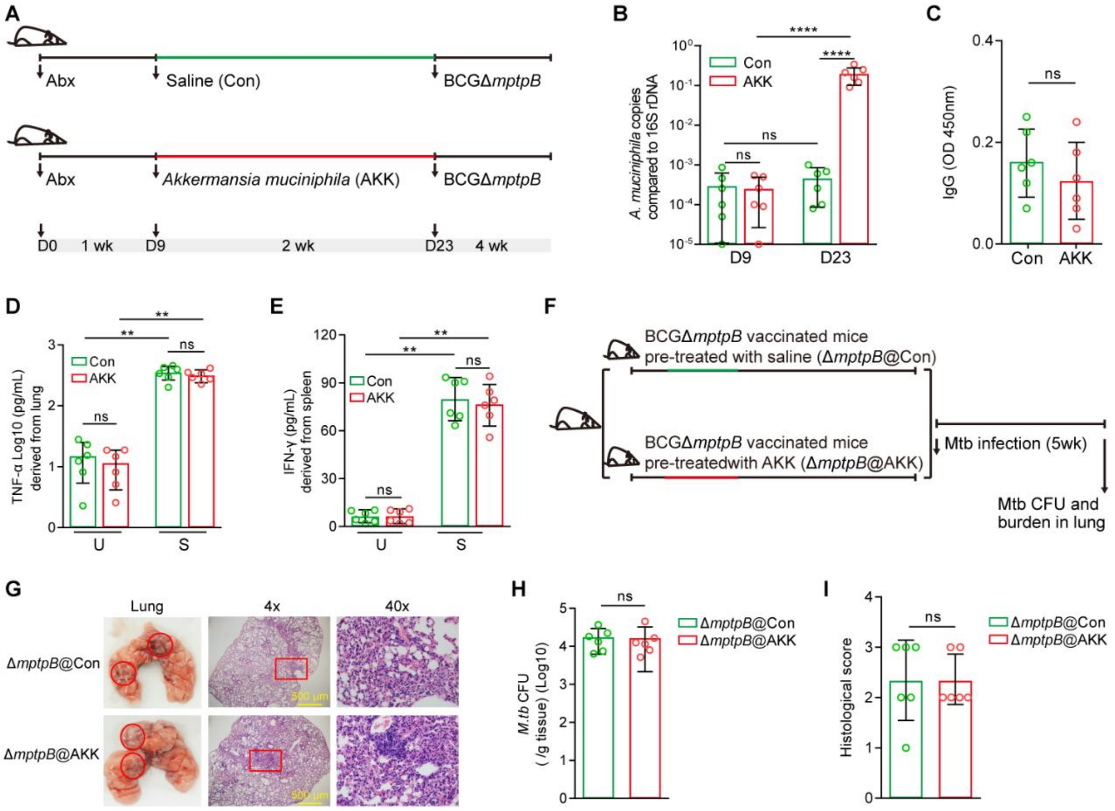

Figure S21. The function of *A. muciniphila* on BCG vaccination is mediated by the effector protein MptpB of BCG.

(A) Schematic diagram of MptpB in the function of *A. muciniphila* to BCG. (B) *A. muciniphila* abundance in stool samples. (C) IgG concentrations in sera of mice that received BCGΔ*mptpB* vaccine after 4 weeks. (D-E) Expression levels of TNF-α in lung (D) and IFN-γ in spleen (E) stimulated with (S) or without (U) Mtb lysates. (F) Assessment of protective efficacy of BCGΔ*mptpB*-vaccinated mice (Δ*mptpB*@Con and Δ*mptpB*@AKK) after infected with Mtb for 5 weeks. (G) H&E staining of two representative lungs. (H-I) Numbers of intracellular Mtb CFU (H) and histological scores (I) in lung tissue. Data are presented as mean ± SD. In vivo animal experiments included at least two biological replicates. N = 6 per group in the mouse model. Mann Whitney test was used to assess statistical significance. Two-way ANOVA with Tukey correction for (B). *P < 0.05, **P < 0.01, ***P < 0.001, and ****P < 0.0001; ns, not significant.

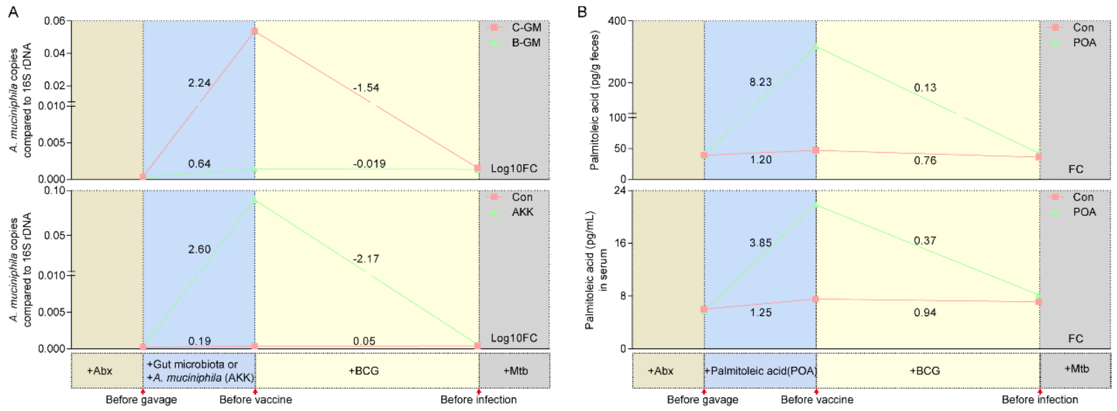

Figure S22. Longitudinal follow-up of *A. muciniphila* and palmitoleic acid during the experimental schema of mice. (A) Analysis of *A. muciniphila* abundance in faecal samples of mice subjected to microbiota transplantation or *A. muciniphila* gavage. (B) Concentration of palmitoleic acid in faecal and serum samples of mice treated with dietary palmitoleic acid. Data are presented as mean ± SEM. Samples were collected at the indicated time points, as shown in the experimental schema. N = 6 per group.

Table S1 Primers used in this study

V3-V4	forward primer 5′-ACTCCTACGGGAGGCAGCA-3′
	reverse primer 5′-GGACTACHVGGGTWTCTAAT-3′
A. muciniphila	forward primer 5′-CAGCACGTGAAGGTGGGGAC-3′
	reverse primer 5′-CCTTGCGGTTGGCTTCAGAT-3′
16S rDNA	forward primer 5′-CGGTGAATACGTTCCCGG-3′
	reverse primer 5′-TACGGCTACCTTGTTACGACTT-3′
IL6	forward primer 5′-CTGCAAGAGACTTCCATCCAG-3′
	reverse primer 5′-AGTGGTATAGACAGGTCTGTTGG-3′
TNF-α	forward primer 5′-CCTGTAGCCCACGTCGTAG-3′
	reverse primer 5′-GGGAGTAGACAAGGTACAACCC-3′
IL-1β	forward primer 5′-GAAATGCCACCTTTTGACAGTG-3′
	reverse primer 5′-TGGATGCTCTCATCAGGACAG-3′
ACTB	forward primer 5′-GTGACGTTGACATCCGTAAAGA-3′
	reverse primer 5′-GCCGGACTCATCGTACTCC-3′

Table S2 Baseline characteristics of public data

Source	Region	Cohort/ group	Sample size		dataset	Age	Gender	BMI
Human	Europe	Western european	321		PRJNA685797	23 (18-75)	F: 183 M: 138	83% (18.5-25)
	Asia	Shenzhen (China)	90	54	PRJNA609532	38.3 (22-66)	F: 18 M: 36	72% (18-25)
		Foshan (China)		36		33.92 (17-57)	F: 13 M: 23	75% (18-25)
Cell	-	RNA-seq<br>ATAC-seq	3 sample per group		PRJNA992960<br>PRJNA993298<br>PRJNA993603	BCG-Russia, BCG-Sweden, BCG-China, BCG-Pasteur		

Tbale S3 Characteristics of gut microbiota in vaccination-adapted mice

Taxonomy	C-GM	C-GM	C-GM	C-GM	C-GM	C-GM	B-GM	B-GM	B-GM	B-GM	B-GM	B-GM
Citrobacter_freundii	0	0.001920218	0.00016518	0.001094318	0	0.000454245	0.001899571	0.00156921	0.001135613	0.000887843	0.003675256	0.000702015
Herbaspirillum_huttiense	0	0	0	0	0	0	0	0	0	0	0	0
Akkermansia_muciniphila	0.007164685	0.023228444	0.036876445	0.050689627	0.083106211	0.120973736	2.06E-05	0.000784605	0.000309713	0.001053023	0.001631153	0.004645689
Klebsiella_oxytoca	0	0.001920218	0.008155765	0.014329369	0	0.01647671	0.032953419	0.023104559	0.015650809	0.016538652	0.036153783	0.012409151
Lactobacillus_murinus	0.002415758	0.007040799	0.001094318	0.000103238	0.002560291	0.000227123	0.042657747	0.076602246	0.04503221	0.002333168	0.003159068	0.00074331
Faecalibaculum_rodentium	0.012471093	4.13E-05	0.013937066	0.081805418	0.064936406	0.032953419	0.000619425	0.001383383	0.001114965	0.013028576	0.14114635	0.081309878
Lactobacillus_johnsonii	4.13E-05	0.012388503	8.26E-05	0	0	0.000867195	0.055190783	0.06225223	0.051040634	0.00156921	0.003489428	2.06E-05
Lachnospiraceae_bacterium_28-4	0.036215725	6.19E-05	0.006421374	0.007164685	0.013049224	0.007474397	0.00033036	0.005099934	8.26E-05	0.19503634	0.029298811	0.003943674
Escherichia_coli	6.19E-05	0.009724975	0.000123885	0.000144533	0	8.26E-05	0.059051867	0.014535844	0.010881236	0.000144533	0.000640073	2.06E-05
Ileibacterium_valens	0.015113974	0.000227123	0.043111992	0.036442848	0.075507929	0.02797737	0.005409647	0.004914106	0.009993393	0.053270565	0.054199703	0.061467625
Parabacteroides_goldsteinii	0.002002808	0.002870003	0.000557483	0.000929138	0.000433598	0.000949785	0.014866204	0.013338289	0.00935332	0.000371655	0.000103238	0.001527915
Bacteroides_acidifaciens	0.01796333	0.018355633	0.008176412	0.003964321	0.004934754	0.006999504	0.006421374	0.006565907	0.004728279	0.00976627	0.006545259	0.008011232
Corynebacterium_kutscheri	0	0.004026264	0	0	0	0	0	0	0	4.13E-05	0.000123885	0
Bacteroides_vulgatus	0.074950446	0.002498348	0.036649323	0.029505286	0.04329782	0.042079617	2.06E-05	0	0	0.033614139	0.017901388	0.063635613
Lactobacillus_reuteri	0.00016518	0.015795342	6.19E-05	0	6.19E-05	0.000227123	0.023083911	0.029959531	0.019718368	0.000516188	0.00090849	0
Mucispirillum_schaedleri	0.001053023	2.06E-05	0.00066072	0.001362735	0.000763958	0.00099108	0.00099108	0.002333168	0.000392303	0.000309713	0.000392303	0.016001817
Staphylococcus_aureus	0	0.000227123	0	4.13E-05	0	0	0.000123885	0	0.000123885	6.19E-05	0.000227123	0
Enterococcus_casseliflavus	2.06E-05	0.00033036	0	8.26E-05	6.19E-05	6.19E-05	0	0	0.000144533	0	0	0
Firmicutes_bacterium_M10-2	0	0.058122729	0	0	0	0	0	0	0	0	0	0
Romboutsia_ilealis	0.005657417	0.039643211	0.002828708	0.006297489	0.008217707	0.001032375	8.26E-05	0	2.06E-05	0.008320945	0.020151966	0.002085398
Staphylococcus_sciuri	0	0.00016518	0	0	0	0	0	0	0	0	8.26E-05	0

Bifidobacterium_pseudolongum	0.000227123	0.004170796	0.000970433	0.000763958	0.000805253	0.000454245	0.005905187	0.031033201	0.008548067	0.000619425	0.000640073	0.000805253
Bacteroides_caecimuris	0.002663528	0.002209283	0.00049554	0.00016518	0.00049554	0.000433598	0.001053023	0.00107367	0.001713743	0.000681368	0.000557483	0.000681368
Lachnospiraceae_bacterium_DW59	0.000949785	0.000123885	0.01009663	0.007102742	0.002250578	0.003159068	0.001610505	0.000640073	0.000929138	0.000433598	2.06E-05	0.002250578
Blautia_sp_YL58	0	0.006152957	0	0.000268418	6.19E-05	0.000309713	0	0	0	0	0	4.13E-05
Bacillus_niabensis	0	0	0.001816981	0.003055831	2.06E-05	0	0	0	0	0	0.011707136	2.06E-05
Pseudomonas_fragi	2.06E-05	0	0	0	0	2.06E-05	0	0	0	0	0.025644202	2.06E-05
bacterium	0.000123885	0	0.000227123	0.000268418	0	0	6.19E-05	0	0	0	0.003489428	0
Lachnospiraceae_bacterium_615	0.000433598	0	0.00107367	0.0008259	0.002931946	0.00107367	0.000103238	0.000970433	0.000123885	0.000268418	8.26E-05	4.13E-05
Proteus_vulgaris	0	0	0	4.13E-05	0	0	0.000722663	0.000516188	0.00074331	0	0	0
Desulfovibrio_fairfieldensis	0.004067559	0.000763958	0.00836224	0.006049719	0.010654113	0.007763462	0.001259498	0.000103238	0.000268418	0.004418566	0.001775685	0.017529732
Dorea_sp_5-2	0.013049224	0.001631153	0.00819706	0.007412455	0.007556987	0.004170796	0.003097126	0.003489428	0.002147341	0.004625041	0.004769574	0.012925339
Clostridiales_bacterium_CIEAF_020	0.000206475	0.001775685	0.000103238	0	0.000227123	0	8.26E-05	0.00107367	0.000103238	0	0	0.000103238
Lachnospiraceae_bacterium_COE1	0.002704823	2.06E-05	0.009869508	0.000185828	0.000371655	0.000681368	0.00016518	0.000702015	0.000144533	0.00049554	0.000185828	0.00140403
Ralstonia_pickettii	0	0	0	0	0	0	0	0	0	4.13E-05	0	0
Clostridium_sp_CL-6	0	0	0.000681368	0.007061447	0.001135613	0.00861001	0	0	0	0	0	0.00156921
Clostridium_sp_Clone-44	2.06E-05	0	2.06E-05	0.000144533	0.000289065	0	0.000371655	0.000309713	0.000206475	0.000123885	6.19E-05	0
Helicobacter_typhlonius	0	8.26E-05	0	2.06E-05	0	0	0	0	0	0	0	0
Bacteroides_ovatus	0.001114965	0.00016518	0.000351008	0.000289065	0.000289065	0.000681368	4.13E-05	0	6.19E-05	0.000185828	6.19E-05	0.00033036
Acinetobacter_johnsonii	0	0	0	0	2.06E-05	0	0.00016518	0	0	0	0.006421374	0
bacterium_YC-ZSS-LKJ57	0	0	0.00033036	0.000392303	0	0	0	0	0	0	0.000103238	0
Psychrobacter_alimentarius	0	0	0	0	0	0	0	0	0	0	0.005058639	0
Paenibacillus_xylanexedens	0	0	0	0	0	0	0	0	0	0	0.000103238	0

Halomonas_sp	0	0	0	0	0	0	0	0	0	0	2.06E-05	0
Phascolarctobacterium_faecium	0	0	6.19E-05	0.001218203	0.000185828	0.00090849	0	0	0	0	0	8.26E-05
Lactococcus_garvieae	4.13E-05	0.001858276	0	0	0	0	0	6.19E-05	0	0	0	0
Candidatus_Arthromitus_sp_SFB-mouse-NL	0	0.000433598	0	0	0	0	0.000454245	0.000351008	0.000185828	0.000144533	0.000536835	0
Lactobacillus_intestinalis	0	0.004170796	0	0	0	0.000536835	0.00074331	0	0	4.13E-05	0	0
Bacillus_sp_Y1	6.19E-05	0	4.13E-05	4.13E-05	0	0	8.26E-05	0	0	0	0.003799141	0
Burkholderiales_bacterium_YL45	0	0.000454245	2.06E-05	0.000867195	8.26E-05	0.000702015	0	0	0	0	0	0.000144533
Prevotella_sp_RS2	0	0	0	0	0	0.003923026	0	0	0	0	0	0
Dialister_propionicifaciens	0	0.002518996	0	0	0	0	0	0	0	0	0	0
Bacillus_alkalitelluris	2.06E-05	0	0	2.06E-05	0	0	0	0	0	0	0.002601586	0
Prevotella_corporis	0	0.001693095	0	0	0	0	0	0	0	0	0	0
Microvirga_aerilata	8.26E-05	0	4.13E-05	4.13E-05	0	0	8.26E-05	0	0	0	0	0
Pseudomonas_oryzihabitans	0	0	0	0	0	0	0	0	0	0	0.000103238	0
Bifidobacterium_animalis	0	2.06E-05	0	0	0	0	0	0.000123885	0	0	0	0
Fusobacterium_periodonticum	0	2.06E-05	0	0	0	0	0	0	0	0	0	0
Prevotella_sp_DJF_LS16	0	0	0	0	0	0.002808061	0	0	0	0	0	0
Photobacterium_halotolerans	0	0	0	0	0	0	0	0	0	0	0.001197555	0
Clostridioides_difficile	6.19E-05	0.002539643	2.06E-05	0.000557483	0.000227123	0.001259498	0	0	0	0	0	4.13E-05
Enterococcus_faecalis	0	2.06E-05	0	0	0.000557483	0	0	0	0.000784605	8.26E-05	0.000227123	0
Archangium_gephyra	0.000103238	0	0.000289065	0.000433598	0	0	0.00016518	0	0	0	6.19E-05	0
Prevotella_timonensis	0	0.001383383	0	0	0	0	0	0	0	0	0	0
Parabacteroides_sp	0	0.000103238	0	0	0	0	0	0	0	0.000268418	0.00024777	0

Eubacterium_sp_14-2	0.000619425	0	0.00049554	0.00016518	0.000123885	4.13E-05	6.19E-05	2.06E-05	0	0	0.000371655	0.000516188
Veillonellaceae_bacterium_DNF00626	0	0.001982161	0	0	0	0	0	0	0	0	0	0
Corynebacterium_variabile	0	0	0	0	0	0	0	0	0	0	2.06E-05	0
Rhodococcus_erythropolis	0	0	0	0	0	0	0.001858276	0	0	0	0	0
Lolium_perenne	4.13E-05	0	4.13E-05	4.13E-05	0	0	0	0	2.06E-05	0	8.26E-05	0
Campylobacter_hominis	0	0.000474893	0	0	0	0	0	0	0	0	0	0
Corynebacterium_mastitidis	0	0	0	0	4.13E-05	0	0	0	4.13E-05	0	0	0
Blautia_obeum	0	6.19E-05	0	0	0	0.000640073	0	0	0.00016518	0	0	0
Bacillus_anthracis	0	0	0	0	0	0	6.19E-05	2.06E-05	0	0	0	0
Porphyromonadaceae_bacterium_DJF_B175	0	0	0	0	0	0.001342088	0	0	0	0	0	0
Dialister_sp_Marseille-P5638	0	0	0	2.06E-05	0	0	0	0	0	0	0	0
Bacteroides_uniformis	0	6.19E-05	0.000123885	0.000206475	4.13E-05	0.00016518	2.06E-05	0	0	2.06E-05	0	0.000144533
Kocuria_rosea	4.13E-05	0	0.000123885	0.00016518	0	0	0.000123885	0	0	0	0.001259498	4.13E-05
Halomonas_alkaliphila	0	0	0	0	0	0	2.06E-05	0	0	0	4.13E-05	0
Exiguobacterium_sp_AT1b	0	0	0	0	0	0	0	0	0	0	0.000846548	0
Pseudomonas_azotoformans	0	0	0	0	0	0	0	0	0	0	0.001300793	0
Clostridium_sp_SY8519	0	0	0	0	0	0.000185828	0	0	0	0	0	0
Lactococcus_lactis	2.06E-05	0	0	4.13E-05	0.000185828	4.13E-05	0	0	0	0	0.000309713	8.26E-05
Bacteroidales_bacterium_KA00251	0	0.001218203	0	0	0	0	0	0	0	0	0	0
Rothia_aeria	0	0	0	0	0	0	0	0	0	0	2.06E-05	0
Porphyrobacter_mercurialis	0	0	0.000144533	0.000268418	0	0	0	0	0	0	0	0
Terribacillus_aidingensis	0	0	0	0	0	0	0	0	0	0	0.000309713	0

Ruminococcus_sp_N15.MGS-57	0	2.06E-05	0	0	0	0.000433598	0	0	0	0	0	0
Acutalibacter_muris	0.000784605	0	0.000536835	0.000289065	0.000536835	0.00016518	8.26E-05	0.000227123	0.00016518	0.000474893	0.000474893	0.00041295
Brochothrix_thermosphacta	0	0	0	0	0	0	0	0	0	0	0.00016518	0
Alcanivorax_venustensis	0	0	0	0	0	0	0	0	0	0	0.000474893	0
Microbulbifer_sp_YIM_C306	0	0	0	0	0	0	0	0	0	0	0.00016518	0
Prevotella_disiens	0	0.00057813	0	0	0	0	0	0	0	0	0	0
Acinetobacter_guillouiae	0	0	0	0	0	0	6.19E-05	0	0	0	0.000929138	0
Bacillus_halmapalus	0	0	6.19E-05	0	0	0	0	0	0	0	0.000144533	0
Anaerostipes_hadrus	0	0.000123885	0	0	0	2.06E-05	0	0	0	0	0	0
Bacteroides_fragilis	0	0.000846548	0	0	0	0	0	0	0	0	0	0
Clostridium_sp_Culture-27	0	0.00016518	0	0	0	0	0.000227123	0.000268418	0.000433598	0	2.06E-05	0
Weissella_cibaria	0	0	0	0	0	0	0	0	0	2.06E-05	0	0
Prevotella_colorans	0	0.00074331	0	0	0	0	0	0	0	0	0	0
Microbulbifer_okinawensis	0	0	4.13E-05	8.26E-05	0	0	0	0	0	0	0.000206475	0
Sutterella_parvirubra	0	0	0	0	0	0	0	0	0.000681368	0	0	0
Nitrospira_bacterium_SG8_3	0	0	0.00066072	0.000227123	0	0	0	0	0	0	0.00016518	0
Bacteroides_cellulosilyticus	0	0	0	0	2.06E-05	0	0	0	0	0	0	0
Streptococcus_salivarius_subsp_thermophilus	0.000144533	0	4.13E-05	6.19E-05	6.19E-05	6.19E-05	0	0	0	4.13E-05	0.00066072	6.19E-05
Alistipes_inops	0.00049554	0	0.000123885	0.000309713	0.000268418	0.00016518	2.06E-05	2.06E-05	0	0.000268418	0.00016518	0.00033036
Parabacteroides_sp_CT06	0	4.13E-05	4.13E-05	0.000392303	0.000103238	0.000392303	0	0	0	0	0	8.26E-05
Fictibacillus_arsenicus	0	0	0.00057813	0.000103238	0	0	4.13E-05	0	0	0	0.000144533	0
Erysipelatoclostridium_ramosum	0	0.000351008	0	0	0	0	0	0	0	0	0	0

bacterium_MI-37	0	0	0	0	0	0	0	0	0	0	0.000309713	0
Bradyrhizobium_elkanii	0.00016518	0	4.13E-05	0	0	0	0	0	0	0	0	0
Cutibacterium_acnes	0	0	4.13E-05	0	0	0	0.000123885	0.00016518	2.06E-05	0	0	0
[Clostridium]_leptum	0	0.000185828	0	0	0	0	0.000227123	0.00049554	0.000289065	0	0	0
Prevotella_buccalis	0	0.000103238	0	0	0	0	0	0	0	0	0	0
Comamonas_denitrificans	6.19E-05	0	4.13E-05	2.06E-05	0	0	0.00049554	0	0	0	6.19E-05	0
Cystobacter_fuscus	0	0	0	6.19E-05	0	0	0	0	0	0	0	0
Brevundimonas_vesicularis	4.13E-05	0	0	0	0	0	8.26E-05	0	4.13E-05	0	2.06E-05	0
Rothia_kristinae	0	0	0	0	0	0	0	0	0	0	0.000268418	0
Lactobacillus_delbrueckii	4.13E-05	0	0	0	4.13E-05	4.13E-05	0	0	0	2.06E-05	8.26E-05	2.06E-05
Parasutterella_secunda	0	0	0	0	0	0.000227123	0	0	0	0	0	0
Streptococcus_merionis	0	0.000123885	0	0	0	0	0	0	0	0	0	0
Novosphingobium_sp_P6W	0	8.26E-05	0	6.19E-05	0	0	0	0	0	0	0.000103238	0
Peptoniphilus_sp_BV3AC2	0	0.000392303	0	0	0	0	0	0	0	0	0	0
Neorhizobium_sp_NCHU2750	0	0	0	0	0	0	8.26E-05	0	0	0	0.000144533	0
Azospirillum_sp_47_25	0.00024777	0.000371655	8.26E-05	2.06E-05	0	2.06E-05	2.06E-05	6.19E-05	0	8.26E-05	0.000144533	0
Solibacillus_silvestris	0	0	0	0	0	0	0	0	0	0	4.13E-05	0
Facklamia_tabacinasalis	0	2.06E-05	6.19E-05	0	0	0	0	0	0	0	8.26E-05	0
Bacteroides_caccae	0	0.000351008	0	0	0	0	0	0	0	0	0	0
Anoxybacillus_flavithermus	0	0	0	6.19E-05	0	0	4.13E-05	0	0	0	0	0
Porphyromonadaceae_bacterium_C941	0	0.00033036	0	0	0	0	0	0	0	0	0	0
Dermacoccus_nishinomiyaensis	0	0	0	0	0	0	0	0	0	0	0.000309713	0

Arenimicrobium_luteum	2.06E-05	0	0	0	0	0	0	0	0	0	0	0
Kofleria_flava	0	0	0	2.06E-05	0	0	0	0	0	0	0	0
Halomonas_sp_1513	0.000289065	0	0	0	0	0	0.000268418	0	0	0	0	0
Firmicutes_bacterium_CAG_194_44_15	0	0	0	0	0	0	0	0	2.06E-05	0	0	0
Parabacteroides_merdae	6.19E-05	0	0	0	0	0	0	0	0	0	0	0
Actinomycetales_bacterium	2.06E-05	0	0	2.06E-05	0	0	8.26E-05	0	0	0	0	0
Nitrospira_sp	0	0.000103238	0	0	0	0	0	0	0	0	0	0
Peptoniphilus_lacrimalis	0	0.000227123	0	0	0	0	0	0	0	0	0	0
Bacteroides_coprocola	0	0	0	0	0	8.26E-05	0	0	0.000268418	4.13E-05	0	0
Nordella_oligomobilis	4.13E-05	0	0	0	0	0	0.000103238	0	0	0	0	0
Pantoea_cypripedii	0	6.19E-05	0	2.06E-05	0	0	6.19E-05	0	0	0	0.000123885	0
Vibrio_metschnikovii	0	0	0	0	0	2.06E-05	0	0	0	0	4.13E-05	0
Isoptericola_halotolerans	0	0	4.13E-05	0.000103238	0	0	0	0	0	0	4.13E-05	0
Lysinibacillus_massiliensis	4.13E-05	0	0	0	0	0	0	0	0	0	4.13E-05	0
Hymenobacter_sp_29F	4.13E-05	0	2.06E-05	0	0	0	0	0	0	0	0	0
Paenibacillus_prosopidis	4.13E-05	0	0.000103238	0.000123885	0	0	0	0	0	0	2.06E-05	0
Peptoniphilus_sp_KHD5	0	0.000227123	0	0	0	0	0	0	0	0	0	0
Hydrogenophilus_thermoluteolus	4.13E-05	0	4.13E-05	0	2.06E-05	0	0	0	0	0	4.13E-05	4.13E-05
Massilia_albidiflava	0	0	0	4.13E-05	0	0	0	0	0	0	4.13E-05	0
Prevotella_bivia	0	8.26E-05	0	0	0	0	0	0	0	0	0	0
Ruminococcaceae_bacterium_GD6	4.13E-05	2.06E-05	0	0.000103238	2.06E-05	0.00016518	2.06E-05	4.13E-05	0	2.06E-05	0.000144533	0
Brachybacterium_paraconglomeratum	0	0	0	0	0	0	0	0	0	0	0.000185828	0

Aerococcus_urinaeequi	0	0	0	2.06E-05	0	0	0	0	0	0	4.13E-05	0
Bacteroidaceae_bacterium_DJF_B220	0	0	0	0	0	0.000103238	0	0	0	0	0	0
Clostridium_bornimense	0	0	0	0	0	0.000185828	0	0	0	0	0	0
Clostridium_sp_K4410.MGS-306	0	4.13E-05	4.13E-05	4.13E-05	0	2.06E-05	2.06E-05	0.000185828	0	0.00016518	0	4.13E-05
Thermomonas_hydrothermalis	0	0	0	0	0	0	0	0	0	0	0.00016518	0
Saccharothrix_texasensis	2.06E-05	0	0	0	0	0	4.13E-05	0	0	0	0	0
Bacteroides_massiliensis	0	0.000144533	0	0	0	0	0	0	0	4.13E-05	4.13E-05	0
Rhizobium_sp_NT-26	2.06E-05	0	0	8.26E-05	0	0	0	0	0	0	2.06E-05	0
Gaiella_sp_EBR4-RS1	6.19E-05	0	6.19E-05	0	0	0	4.13E-05	0	0	0	0	0
Clostridium_perfringens	0	0	0	0	0	0	0.000103238	0	0	0	0	0
Anaerofustis_stercorihominis	0	0	0	0	0	0	8.26E-05	8.26E-05	6.19E-05	4.13E-05	0	0
Paenibacillus_thermoaerophilus	0	0	0.000123885	0	0	0	0	0	0	0	2.06E-05	0
Helicobacter_bilis	0	0.000123885	0	0	6.19E-05	0	0	0	0	0	4.13E-05	4.13E-05
Inquilinus_limosus	0	0	0	8.26E-05	0	0	0	0	0	0	0	0
Turicibacter_sp_LA61	0	0.000123885	0	0	0	0	4.13E-05	8.26E-05	4.13E-05	0	0	0
Oceanobacillus_picturae	0	0	2.06E-05	0	0	0	0	0	0	0	8.26E-05	0
Actinomadura_sp	6.19E-05	0	0	4.13E-05	0	0	0	0	0	0	0	0
Lactobacillus_iners	8.26E-05	4.13E-05	0	0	0	0	0	0	0	0	0	0
delta_proteobacterium_WX81	0.000103238	0	0	0	0	0	0	0	0	0	0	0
bacterium_YE57	0	0	0.000103238	2.06E-05	4.13E-05	0	0	0	0	0	0	4.13E-05
Palleronia_marisminoris	0	0	0	0	0	0	0.000103238	0	0	0	0	0
bacterium_3-5_YC-ZSS-LKJ-3024	0	0	0.000103238	8.26E-05	0	0	0	0	0	0	0	0

Megasphaera_elsdenii	0	0	0	0	0	0.000103238	0	0	0	0	0	0
Streptococcus_agalactiae	0	0	0	0	0	0	0	0	0	0	0.000103238	0
Lysobacter_yangpyeongensis	0	0	0	0	0	0	4.13E-05	0	0	0	0	0
Veillonella_magna	8.26E-05	0	2.06E-05	0	0	0	0	0	0	0	0	0
Streptococcus_hyointestinalis	0	6.19E-05	0	2.06E-05	4.13E-05	0	0	0	0	0	0	0
Cellvibrio_diazotrophicus	0	0	6.19E-05	0	0	0	0	0	0	0	0	0
Campylobacter_concisus	0	0	0	0	0	0	0	0	0	0	0	0
Ruminococcaceae_bacterium_Marseille-P3738	0	0	0	0	0	2.06E-05	6.19E-05	0	0	0	0	0
Rothia_mucilaginosa	0	0	0	0	0	0	0	2.06E-05	0	0	0	0
Rhodospirillaceae_bacterium_PS4B-B496	0	0	0	0	0	0	0	0	0	0	2.06E-05	0
Streptococcus_respiraculi	0	0	2.06E-05	0	0	2.06E-05	0	0	0	0	0	0
Sphingobacterium_faecium	0	0	0	0	0	0	0	0	0	0	6.19E-05	0
Coxiella_burnetii	0	0	0	4.13E-05	0	0	0	0	0	0	6.19E-05	0
Christensenella_sp_Marseille-P2437	0	4.13E-05	0	0	0	0	0	0	4.13E-05	0	0	0
Anaerococcus_lactolyticus	4.13E-05	4.13E-05	0	0	0	0	0	0	0	0	0	0
Others	0.794805088	0.75861001	0.793979187	0.725140403	0.673294516	0.697720515	0.735319623	0.715415428	0.811983812	0.649446647	0.599108028	0.703625702

Table S4 Significantly altered metabolites of *A. muciniphila*

Name	Log2FC	pvalue
1-Monopalmitin	-7.199	2.117E-07
glycine	-6.369	5.309E-05
proline	-4.975	0.00916
trehalose	-3.765	0.00671
2-hydroxypyridine	-2.092	3.229E-05
5-aminovaleric acid lactam	1.706	0.00509
hypoxanthine	2.190	0.00254
phenylalanine	2.562	0.00246
histidine	2.642	0.00315
N-Methyl-DL-alanine	2.673	3.624E-05
5-Methoxytryptamine	2.836	0.000185
palmitoleic acid	2.925	0.00678
unknown	4.908	0.000899
3-hydroxybutyric acid	5.251	0.000355
2-hydroxybutanoic acid	6.547	0.000110

Table S5 Significant differential metabolites in faecal samples between B-GM and C-GM

Compounds	VIP	p	FC	Log2FC	Type
(±)12-HETE	1.562	0.008	4.677	2.226	up
(±)15-HETE	1.590	0.000	3.113	1.638	up
(±)5-HETE	1.409	0.006	4.004	2.001	up
(±)9-HETE	1.409	0.006	4.004	2.001	up
(R)-Lipoic Acid	1.747	0.000	6847.463	12.741	up
15-oxoETE	1.467	0.003	2.738	1.453	up
1-Aminocyclohexanoic acid	1.346	0.003	2.825	1.498	up
1-Methyladenine	1.408	0.008	0.322	-1.637	down
2'-Deoxyuridine	1.430	0.008	6.355	2.668	up
2-hydroxy-2-(4-hydroxy-3-methoxyphenyl)acetic acid	1.431	0.002	1.563	0.644	up
2-Hydroxy-2-methylbutyric acid	1.481	0.003	9.753	3.286	up
2-Hydroxycaprylic acid	1.419	0.007	4.021	2.008	up
3-(pyrazol-1-yl) -L-alanine	1.307	0.006	0.349	-1.520	down
3-Hydroxy-3-Methyl Butyric Acid	1.326	0.009	11.332	3.502	up
3-Iodo-L-Tyrosine	1.484	0.005	0.000	-18.733	down
3-Methyladenine	1.476	0.002	0.306	-1.710	down
4-methyl-2-oxovaleric acid	1.458	0.009	5.506	2.461	up
5-Methoxytryptamine	1.232	0.005	0.036	-4.806	down
5-oxoETE	1.335	0.010	3.041	1.605	up
6-Ketoprostaglandin E1	1.283	0.009	0.010	-6.613	down
Alpha-Mercholic Acid	1.326	0.000	7.604	2.927	up
Cis-11,14,17-Eicosatrienoic Acid(C20:3)	1.525	0.001	5.228	2.386	up
Cis-4-Hydroxy-D-Proline	1.606	0.001	0.255	-1.972	down
Deoxycytidine	1.532	0.000	4.970	2.313	up
Deoxyguanosine	1.474	0.006	7.606	2.927	up
D-Glucarate	1.434	0.010	0.105	-3.250	down
D-Glucose	1.322	0.005	0.348	-1.524	down
Dioxindole	1.479	0.001	3.099	1.632	up
Dl-2-Aminooctanoic Acid	1.394	0.006	0.000	-13.690	down
EPA	1.590	0.001	3.009	1.589	up
Gamma-Glu-Leu	1.358	0.006	2.532	1.340	up
Guanosine 3',5'-Cyclic Monophosphate	1.483	0.008	0.000	-14.709	down
Hexadecanoic Acid(C16:0)	1.565	0.001	0.374	-1.418	down
Indole-3-acetamide	1.453	0.004	0.109	-3.201	down
Isocytosine	1.410	0.006	4.020	2.007	up
L-Histidine	1.231	0.006	0.402	-1.315	down
L-Methionine	1.316	0.008	1.739	0.798	up
L-tyrosine methyl ester 4-sulfate	1.292	0.005	0.012	-6.408	down
L-Valine	1.354	0.006	1.903	0.928	up
Mucic Acid	1.454	0.008	0.106	-3.239	down

N-Acetyl-D-Glucosamine	1.290	0.002	1.904	0.929	up
N-Acetyl-L-Leucine	1.470	0.006	10.420	3.381	up
N-Acetylphenylalanine	1.495	0.008	8.727	3.125	up
N-Alpha-Acetyl-L-Asparagine	1.359	0.002	0.234	-2.097	down
Nα-Acetyl-L-glutamine	1.636	0.003	0.096	-3.375	down
O-Phospho-L-Serine	1.568	0.000	4.731	2.242	up
Palmitoleic Acid(C16:1)	1.466	0.007	0.564	-0.826	down
P-hydroxyphenylacetylglycine	1.547	0.002	0.196	-2.354	down
Prostaglandin E2	1.384	0.007	0.008	-6.913	down
Pterine	1.550	0.000	3.406	1.768	up
Pyrrole-2-Carboxylic Acid	1.415	0.006	2.968	1.569	up
Ritalinic acid	1.477	0.003	6.814	2.769	up
Thymidine	1.492	0.001	8.091	3.016	up
TranexamicAcid	1.281	0.007	2.131	1.092	up
Trans-4-Hydroxy-L-Proline	1.572	0.001	0.256	-1.968	down
Trigonelline	1.334	0.006	2.793	1.482	up
Uridine	1.530	0.005	7.720	2.949	up
Uridine triphosphate(UTP)	1.483	0.006	0.000	-13.271	down
Urobilin	1.473	0.003	6.046	2.596	up
Xanthine	1.462	0.001	4.944	2.306	up
