# Supplementary figures and images for: Investigating the role of baseline gut Akkermansia muciniphila and its co-metabolite palmitoleic acid in BCG vaccine efficacy: a preclinical study
Source: eBioMedicine. 2026 Jul 23;130:106404. doi: 10.1016/j.ebiom.2026.106404 (PMC13427514; doi:10.1016/j.ebiom.2026.106404)

## **Western blots**

Figure 5I

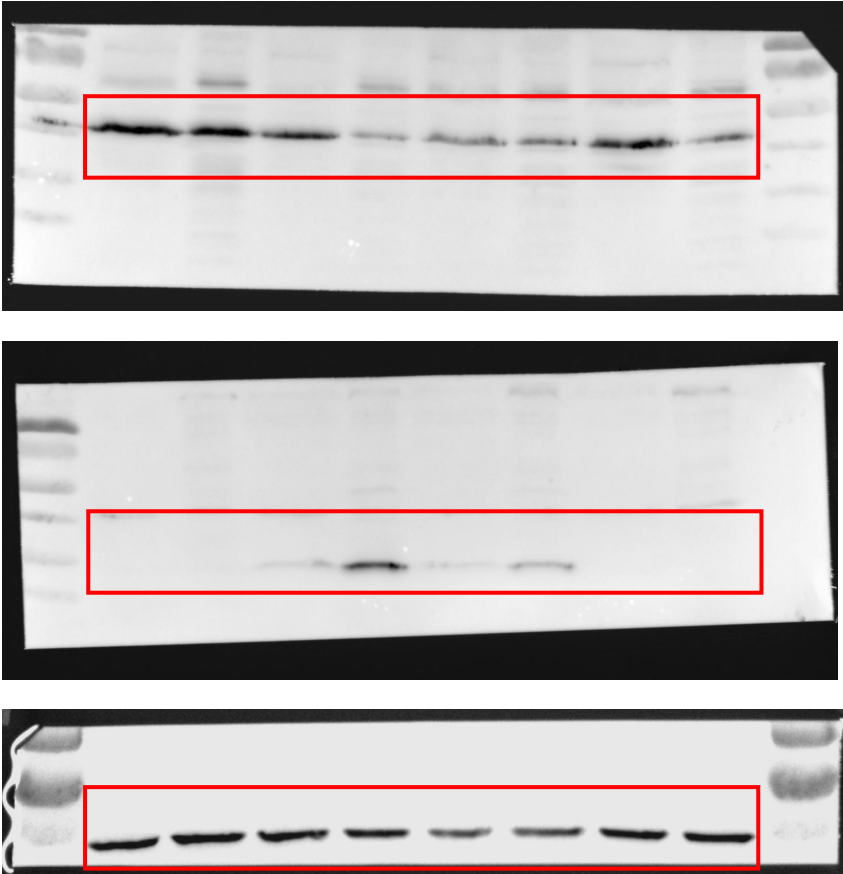

Figure 5J

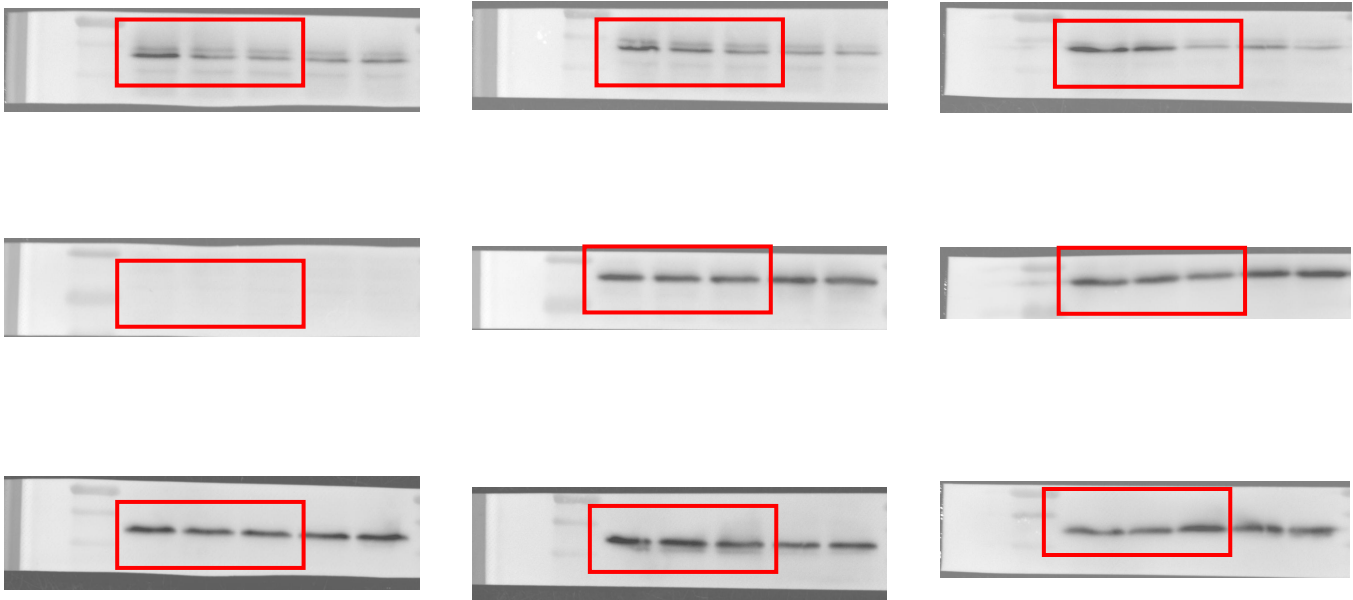

Figure 6l

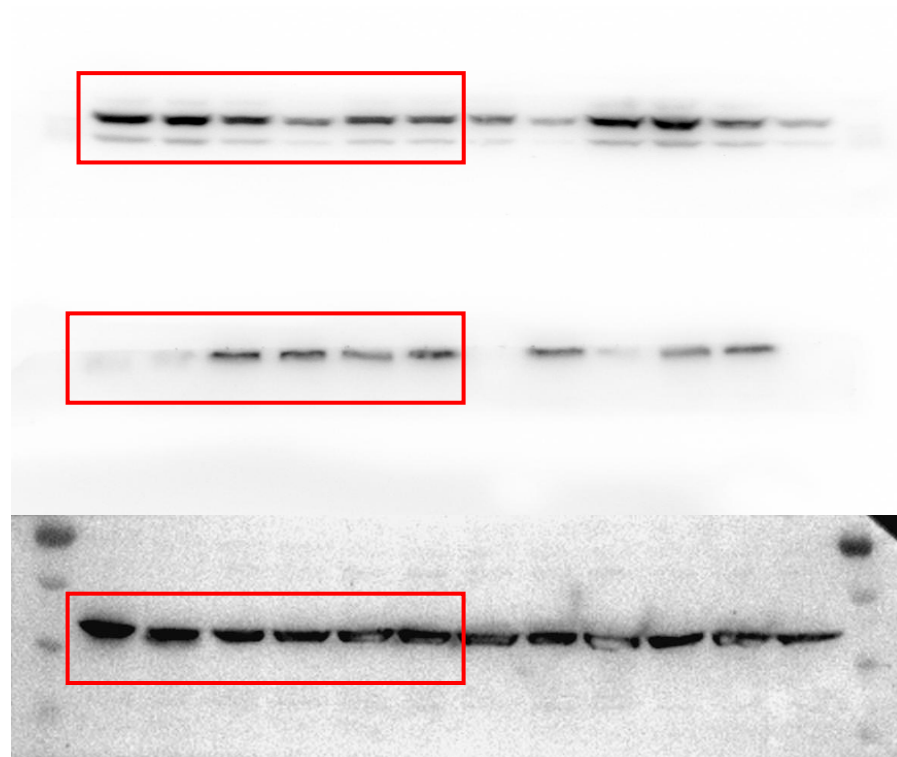

Figure 6K

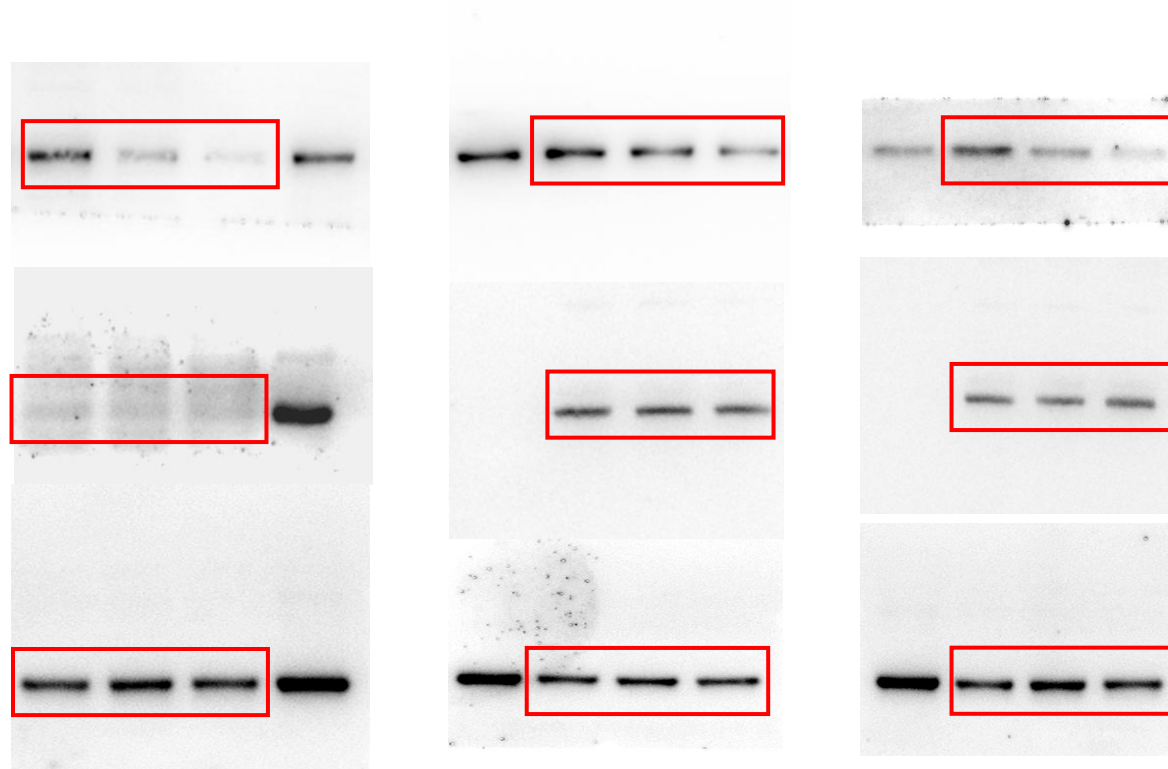

Supplement: Western Blots [file mmc2.pdf]
