# Supplementary material for: Investigating the role of baseline gut Akkermansia muciniphila and its co-metabolite palmitoleic acid in BCG vaccine efficacy: a preclinical study
Source: eBioMedicine. 2026 Jul 23;130:106404. doi: 10.1016/j.ebiom.2026.106404 (PMC13427514; doi:10.1016/j.ebiom.2026.106404)
Supplement: Supplementary Material [file mmc3.pdf]

#M21001

Goat Anti-Mouse IgG HRP

☐ 100 µl

☐ 1000 µl

Orders ■ 400-6123-828
orders@ab-mart.com
Web ■ www.ab-mart.com.cn

BACKGROUND

Chemiluminescent detection systems have emerged as the best all-around method for detection of Western blots. They eliminate the hazards associated with radioactive materials and toxic chromogenic substrates. The speed and sensitivity of these methods are unequalled by traditional alternatives. Because results are generated on film, it is possible to record and store data permanently, and blots detected with chemiluminescent methods are easily stripped for subsequent reprobing with additional antibodies. Horseradish peroxidase (HRP) conjugated secondary antibodies are utilized in conjunction with specifc chemiluminescent substrates to generate the light signal. Horseradish peroxidase-antibody conjugates have a very high turnover rate, giving good sensitivity with short reaction times.

DESCRIPTION

Affnity purifed Goat Anti-Mouse IgG antibody is conjugated to horseradish peroxidase.

STORAGE

Store at -20°C. Stable for one year from the date of shipment.

APPLICATIONS

This product has been optimized for use as a secondary antibody in chemiluminescent applications.

RECOMMENDED ANTIBODY DILUTION

ELISA	1:3000-1:8000
Western blotting	1:3000-1:8000
Dot blot	1:3000-1:8000

COMPANION PRODUCTS

#M21002 Goat Anti-Rabbit IgG-HRP

For *in vitro* research use only and not intended for use in humans or animals.

BioLegend
From Revvity

FITC anti-mouse CD4 Antibody

**Catalog# / Size**	100405 / 50 µg<br>100406 / 500 µg
**Clone**	GK1.5
**Regulatory Status**	RUO
**Other Names**	L3T4, T4
**Isotype**	Rat IgG2b, κ
**Description**	CD4 is a 55 kD protein also known as L3T4 or T4. It is a member of the Ig superfamily, primarily expressed on most thymocytes, a subset of T cells, and weakly on macrophages and dendritic cells. It acts as a coreceptor with the TCR during T cell activation and thymic differentiation by binding MHC class II and associating with the protein tyrosin kinase, lck.

Product Details

**Verified Reactivity**	Mouse
**Antibody Type**	Monoclonal
**Host Species**	Rat
**Immunogen**	Mouse CTL clone V4
**Formulation**	Phosphate-buffered solution, pH 7.2, containing 0.09% sodium azide.
**Preparation**	The antibody was purified by affinity chromatography, and conjugated with FITC under optimal conditions.
**Concentration**	0.5 mg/ml
**Storage & Handling**	The CD4 antibody solution should be stored undiluted between 2°C and 8°C, and protected from prolonged exposure to light. **Do not freeze.**
**Application**	FC - Quality tested
**Recommended Usage**	Each lot of this antibody is quality control tested by immunofluorescent staining with flow cytometric analysis. For flow cytometric staining, the suggested use of this reagent is ≤0.25 µg per million cells in 100 µl volume. It is recommended that the reagent be titrated for optimal performance for each application.
**Excitation Laser**	Blue Laser (488 nm)
**Application Notes**	Additional reported applications (for the relevant formats) include: blocking of $CD4^+$ T cell activation[1,4,11], thymocyte costimulation[3], *in vitro* and *in vivo* depletion[2,5-8], blocking of egg-sperm cell adhesion[1,4], immunohistochemical staining of acetone-fixed frozen sections[9,10], immunoprecipitation[1,2], and spatial biology (IBEX)[12,13]. The GK1.5 antibody is able to block CD4 mediated cell adhesion and T cell activation. Binding of GK1.5 antibody to CD4 T cells can be blocked by RM4-5 antibody, but not RM4-4 antibody. For *in vivo* studies or highly sensitive assays, we recommend Ultra-LEAF™ purified antibody (Cat. No. 100442) with a lower endotoxin limit than standard LEAF™ purified antibodies (Endotoxin < 0.01 EU/µg).

Application References

(PubMed link indicates BioLegend citation)

1. Dialynas DP, *et al.* 1983. *J. Immunol.* 131:2445. (Block, IP)
2. Dialynas DP, *et al.* 1983. *Immunol. Rev.* 74:29. (IP, Deplete)
3. Wu L, *et al.* 1991. *J. Exp. Med.* 174:1617. (Costim)
4. Godfrey DI, *et al.* 1994. *J. Immunol.* 152:4783. (Block)
5. Gavett SH, *et al.* 1994. *Am. J. Respir. Cell. Mol. Biol.* 10:587. (Deplete)
6. Schuyler M, *et al.* 1994. *Am. J. Respir. Crit. Care Med.* 149:1286. (Deplete)
7. Ghobrial RR, *et al.* 1989. *Clin. Immunol. Immunopathol.* 52:486. (Deplete)
8. Israelski DM, *et al.* 1989. *J. Immunol.* 142:954. (Deplete)
9. Zheng B, *et al.* 1996. *J. Exp. Med.* 184:1083. (IHC)
10. Frei K, *et al.* 1997. *J. Exp. Med.* 185:2177. (IHC)
11. Felix NJ, *et al.* 2007. *Nat. Immunol.* 8:388. (Block)
12. Radtke AJ, *et al.* 2020. *Proc Natl Acad Sci U S A.* 117:33455-65. (SB) PubMed

See More

Product Citations

1. Chuang H, *et al.* 2014. Nat Commun. 5:4602. PubMed
2. Chang YS, *et al.* 2020. Int J Mol Sci. 21:00. PubMed
3. Djokić V, *et al.* 2019. Front Immunol. 9:2891. PubMed
4. Katsumura KR, *et al.* 2018. Proc Natl Acad Sci U S A. 115:E10109. PubMed
5. Valanparambil RM, *et al.* 2017. PLoS Pathog. 13:e1006647. PubMed
6. Wang X, *et al.* 2019. Cell Res. 29:787. PubMed
7. Qiu Z, *et al.* 2022. J Exp Med. 219:. PubMed
8. Lin J, *et al.* 2022. Adv Sci (Weinh). 9:e2202633. PubMed
9. Hamdi L, *et al.* 2022. Ann Clin Transl Neurol. 9:1792. PubMed
10. Deák P, *et al.* 2022. Cell Rep. 41:111563. PubMed
11. Zhu C, *et al.* 2022. Chemotherapy. 67:211. PubMed
12. Pan Y, *et al.* 2023. Adv Sci (Weinh). 10:e2206792. PubMed

RRID AB_312690 (BioLegend Cat. No. 100405)
AB_312691 (BioLegend Cat. No. 100406)

Antigen Details

Structure Ig superfamily, 55 kD

Distribution Majority of thymocytes, T cell subset

Function TCR co-receptor, T cell activation

Ligand/Receptor MHC class II molecule

Cell Type Dendritic cells, T cells, Thymocytes, Tregs

Biology Area Immunology

Molecular Family CD Molecules

Antigen References

1. Barclay A, *et al.* 1997. The Leukocyte Antigen FactsBook Academic Press.
2. Bierer BE, *et al.* 1989. *Annu. Rev. Immunol.* 7:579.
3. Janeway CA. 1992. *Annu. Rev. Immunol.* 10:645.

Gene ID 12504

Related Protocols

- Cell Surface Flow Cytometry Staining Protocol

Other Formats

Brilliant Violet 421™ anti-mouse CD4, Ultra-LEAF™ Purified anti-mouse CD4, Spark Blue™ 515 anti-mouse CD4, Spark UV™ 387 anti-mouse CD4, PE anti-mouse CD4, Spark PLUS UV395™ anti-mouse CD4, APC anti-mouse CD4, Biotin anti-mouse CD4, FITC anti-mouse CD4, Brilliant Violet 510™ anti-mouse CD4, PE/Cyanine5 anti-mouse CD4, Brilliant Violet 711™ anti-mouse CD4, Brilliant Violet 605™ anti-mouse CD4, Alexa Fluor® 594 anti-mouse CD4, GoInVivo™ Purified anti-mouse CD4, Spark YG™ 593 anti-mouse CD4, Spark Blue™ 574 anti-mouse CD4 Antibody, Purified anti-mouse CD4, PerCP/Fire™ 780 anti-mouse CD4 Antibody, Alexa Fluor® 647 anti-mouse CD4, PerCP/Fire™ 806 anti-mouse CD4 Antibody, Alexa Fluor® 488 anti-mouse CD4,

Spark PLUS B488™ anti-mouse CD4, Spark YG™ 581 anti-mouse CD4 (Flexi-Fluor™), Spark PLUS YG581™ anti-mouse CD4, PE/Fire™ 744 anti-mouse CD4, Pacific Blue™ anti-mouse CD4, Spark NIR™ 685 anti-mouse CD4, KIRAVIA Blue 520™ anti-mouse CD4, PE/Fire™ 640 anti-mouse CD4, APC/Cyanine7 anti-mouse CD4, Brilliant Violet 750™ anti-mouse CD4, Spark Blue™ 550 anti-mouse CD4, PE/Cyanine7 anti-mouse CD4, Spark Violet™ 538 anti-mouse CD4, Brilliant Violet 785™ anti-mouse CD4, PE/Dazzle™ 594 anti-mouse CD4, APC/Fire™ 750 anti-mouse CD4, Spark Red™ 718 anti-mouse CD4 (Flexi-Fluor™), StarBright UltraViolet 795 anti-mouse CD4, Spark PLUS V475™ anti-mouse CD4, Alexa Fluor® 700 anti-mouse CD4, PerCP anti-mouse CD4, PerCP/Cyanine5.5 anti-mouse CD4, Brilliant Violet 650™ anti-mouse CD4, APC/Fire™ 810 anti-mouse CD4, PE/Fire™ 700 anti-mouse CD4

Product Data

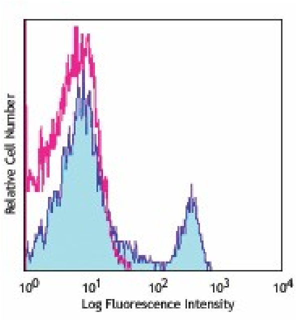

C57BL/6 mouse splenocytes were stained with CD4 (clone GK1.5) FITC (filled histogram) or rat IgG2b, κ FITC isotype control (open histogram).

For Research Use Only. Not for diagnostic or therapeutic use.

This product is supplied subject to the terms and conditions, including the limited license, located at www.biolegend.com/terms) ("Terms") and may be used only as provided in the Terms. Without limiting the foregoing, BioLegend products may not be used for any Commercial Purpose as defined in the Terms, resold in any form, used in manufacturing, or reverse engineered, sequenced, or otherwise studied or used to learn its design or composition without express written approval of BioLegend. Regardless of the information given in this document, user is solely responsible for determining any license requirements necessary for user's intended use and assumes all risk and liability arising from use of the product. BioLegend is not responsible for patent infringement or any other risks or liabilities whatsoever resulting from the use of its products.

BioLegend, the BioLegend logo, and all other trademarks are property of BioLegend, Inc. or their respective owners, and all rights are reserved.

8999 BioLegend Way, San Diego, CA 92121 www.biolegend.com
Toll-Free Phone: 1-877-Bio-Legend (246-5343) Phone: (858) 768-5800 Fax: (877) 455-9587

BioLegend
From Revvity

Version: 2 Revision Date: 01/21/2014

Brilliant Violet 510™ anti-mouse CD8a Antibody

**Catalog# / Size**	100751 / 125 µL<br>100752 / 50 µg
**Clone**	53-6.7
**Regulatory Status**	RUO
**Other Names**	T8, Lyt2, Ly-2
**Isotype**	Rat IgG2a, κ
**Description**	CD8, also known as Lyt-2, Ly-2, or T8, consists of disulfide-linked α and β chains that form the α(CD8a)/β(CD8b) heterodimer and α/α homodimer. CD8a is a 34 kD protein that belongs to the immunoglobulin family. The CD8 α/β heterodimer is expressed on the surface of most thymocytes and a subset of mature TCR α/β T cells. CD8 expression on mature T cells is non-overlapping with CD4. The CD8 α/α homodimer is expressed on a subset of γ/δ TCR-bearing T cells, NK cells, intestinal intraepithelial lymphocytes, and lymphoid dendritic cells. CD8 is an antigen co-receptor on T cells that interacts with MHC class I on antigen-presenting cells or epithelial cells. CD8 promotes T cell activation through its association with the TCR complex and protein tyrosine kinase lck.

Product Details

**Verified Reactivity**	Mouse
**Antibody Type**	Monoclonal
**Host Species**	Rat
**Immunogen**	Mouse thymus or spleen
**Formulation**	Phosphate-buffered solution, pH 7.2, containing 0.09% sodium azide and BSA (origin USA).
**Preparation**	The antibody was purified by affinity chromatography and conjugated with Brilliant Violet 510™ under optimal conditions.
**Concentration**	µg sizes: 0.2 mg/mL<br>µL sizes: lot-specific (to obtain lot-specific concentration and expiration, please enter the lot number in our Certificate of Analysis online tool.)
**Storage & Handling**	The antibody solution should be stored undiluted between 2°C and 8°C, and protected from prolonged exposure to light. **Do not freeze.**
**Application**	FC - Quality tested
**Recommended Usage**	Each lot of this antibody is quality control tested by immunofluorescent staining with flow cytometric analysis. For immunofluorescent staining using the µg size, the suggested use of this reagent is ≤0.5 µg per million cells in 100 µl volume. For immunofluorescent staining using the µl size, the suggested use of this reagent is 5 µl per million cells in 100 µl staining volume or 5 µl per 100 µl of whole blood. It is recommended that the reagent be titrated for optimal performance for each application.<br><br>Brilliant Violet 510™ excites at 405 nm and emits at 510 nm. The bandpass filter 510/50 nm is recommended for detection, although filter optimization may be required depending on other fluorophores used. **Be sure to verify that your cytometer configuration and software setup are appropriate for detecting this channel.** Refer to your instrument manual or manufacturer for support. Brilliant Violet 510™ is a trademark of Sirigen Group Ltd.<br><br>Learn more about Brilliant Violet™.<br><br>This product is subject to proprietary rights of Sirigen Inc. and is made and sold under license from Sirigen Inc. The purchase of this product conveys to the buyer a non-transferable right to use the

purchased product for research purposes only. This product may not be resold or incorporated in any manner into another product for resale. Any use for therapeutics or diagnostics is strictly prohibited. This product is covered by U.S. Patent(s), pending patent applications and foreign equivalents.

Excitation Laser Violet Laser (405 nm)

Application Notes Clone 53-6.7 antibody competes with clone 5H10-1 antibody for binding to thymocytes[3]. The 53-6.7 antibody has been reported to block antigen presentation via MHC class I and inhibit T cell responses to IL-2. This antibody has also been used for depletion of CD8a$^+$ cells. Additional reported applications (for the relevant formats) include: immunoprecipitation[1,3], *in vivo* and *in vitro* cell depletion[2,10,15], inhibition of CD8 T cell proliferation[3], blocking of cytotoxicity[3,4], immunohistochemical staining[5,6] of acetone-fixed frozen sections and zinc-fixed paraffin-embedded sections, and spatial biology (IBEX)[29,30]. Clone 53-6.7 is not recommended for immunohistochemistry of formalin-fixed paraffin sections. The Ultra-LEAF™ purified antibody (Endotoxin < 0.01 EU/µg, Azide-Free, 0.2 µm filtered) is recommended for functional assays or in vivo studies (Cat No. 100746).

Application References

(PubMed link indicates BioLegend citation)

1. Ledbetter JA, *et al.* 1979. *Immunol. Rev.* 47:63. (IHC, IP)
2. Hathcock KS. 1991. *Current Protocols in Immunology.* 3.4.1. (Deplete)
3. Takahashi K, *et al.* 1992. *P. Natl. Acad. Sci. USA* 89:5557. (Block, IP)
4. Ledbetter JA, *et al.* 1981. *J. Exp. Med.* 153:1503. (Block)
5. Hata H, *et al.* 2004. *J. Clin. Invest.* 114:582. (IHC)
6. Fan WY, *et al.* 2001. *Exp. Biol. Med.* 226:1045. (IHC)
7. Shih FF, *et al.* 2006. *J. Immunol.* 176:3438. (FC)
8. Kamimura D, *et al.* 2006. *J. Immunol.* 177:306.
9. Bouwer HGA, *et al.* 2006. *P. Natl. Acad. Sci. USA* 103:5102. (FC, Deplete)
10. Kao C, *et al.* 2005. *Int. Immunol.* 17:1607. PubMed
11. Ko SY, *et al.* 2005. *J. Immunol.* 175:3309. (FC) PubMed
12. Rasmussen JW, *et al.* 2006. *Infect. Immun.* 74:6590. PubMed

See More

Product Citations

1. Saha D *et al.* 2017. Cancer cell. 32(2):253-267 . PubMed
2. Li SX, *et al.* 2019. PLoS Pathog. 15:e1007611. PubMed
3. Wu R, *et al.* 2022. Nat Immunol. 23:1536. PubMed
4. Li Y, *et al.* 2023. Adv Mater. 35:e2208923. PubMed
5. Giampaolo S, *et al.* 2023. iScience. 26:106234. PubMed
6. Byrne PO, *et al.* 2023. Nat Commun. 14:1494. PubMed
7. del Rio ML, *et al.* 2023. Front Immunol. 14:1113858. PubMed
8. Seclì L, *et al.* 2023. J Immunother Cancer. 11:. PubMed
9. Lee K, *et al.* 2023. JCI Insight. 8:. PubMed
10. Wang X, *et al.* 2023. NPJ Vaccines. 8:76. PubMed
11. Luo J, *et al.* 2022. J Nanobiotechnology. 20:228. PubMed
12. Collin R, *et al.* 2020. J Immunol. 205:133. PubMed

RRID AB_2561389 (BioLegend Cat. No. 100751)
AB_2563057 (BioLegend Cat. No. 100752)

Antigen Details

Structure Ig superfamily, CD8α chain, 34 kD

Distribution Most thymocytes, T cell subset, some NK cells, lymphoid dendritic cells

Function Co-receptor for TCR

Ligand/Receptor MHC class I molecule

Antigen References

1. Barclay A, *et al.* 1997. The Leukocyte Antigen FactsBook Academic Press.
2. Zamoyska R. 1994. *Immunity* 1:243.
3. Ellmeier W, *et al.* 1999. *Annu. Rev. Immunol.* 17:523.

Gene ID 12525

Related Protocols

- Cell Surface Flow Cytometry Staining Protocol

Other Formats

Brilliant Violet 421™ anti-mouse CD8a, Brilliant Violet 570™ anti-mouse CD8a, Brilliant Violet 605™ anti-mouse CD8a, Ultra-LEAF™ Purified anti-mouse CD8a, Brilliant Violet 711™ anti-mouse CD8a, Brilliant Violet 510™ anti-mouse CD8a, Brilliant Violet 785™ anti-mouse CD8a, Brilliant Violet 650™ anti-mouse CD8a, Purified anti-mouse CD8a (Maxpar® Ready), Spark UV™ 387 anti-mouse CD8a, Spark Violet™ 423 anti-mouse CD8a Antibody, APC/Cyanine7 anti-mouse CD8a, Spark Blue™ 515 anti-mouse CD8a, APC/Fire™ 810 anti-mouse CD8a, PE/Dazzle™ 594 anti-mouse CD8a, GoInVivo™ Purified anti-mouse CD8a, Alexa Fluor® 594 anti-mouse CD8a, APC anti-mouse CD8a, Spark Blue™ 574 anti-mouse CD8a Antibody, FITC anti-mouse CD8a, Spark YG™ 593 anti-mouse CD8a (Flexi-Fluor™) Antibody, Biotin anti-mouse CD8a, Spark YG™ 581 anti-mouse CD8a (Flexi-Fluor™), PerCP anti-mouse CD8a, PerCP/Fire™ 780 anti-mouse CD8a, Alexa Fluor® 488 anti-mouse CD8a, Alexa Fluor® 647 anti-mouse CD8a, Spark PLUS V475™ anti-mouse CD8a, StarBright UltraViolet 795 anti-mouse CD8a, Spark PLUS YG581™ anti-mouse CD8a, PE anti-mouse CD8a, PE/Cyanine5 anti-mouse CD8a, Purified anti-mouse CD8a, TotalSeq™-A0002 anti-mouse CD8a, PE/Fire™ 810 anti-mouse CD8a, Spark Red™ 718 anti-mouse CD8a (Flexi-Fluor™), Spark NIR™ 685 anti-mouse CD8a, Spark Blue™ 550 anti-mouse CD8a, TotalSeq™-C0002 anti-mouse CD8a, TotalSeq™-B0002 anti-mouse CD8a, PE/Cyanine7 anti-mouse CD8a, Spark YG™ 570 anti-mouse CD8a, PE/Fire™ 640 anti-mouse CD8a, APC/Fire™ 750 anti-mouse CD8a, Spark PLUS UV395™ anti-mouse CD8a, StarBright UltraViolet 575 anti-mouse CD8a, Pacific Blue™ anti-mouse CD8a, Alexa Fluor® 700 anti-mouse CD8a, PerCP/Cyanine5.5 anti-mouse CD8a, PE/Fire™ 700 anti-mouse CD8a

Product Data

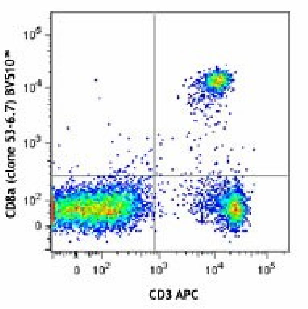

C57BL/6 mouse splenocytes were stained with CD3 APC and CD8a (clone 53-6.7) Brilliant Violet 510™.

For Research Use Only. Not for diagnostic or therapeutic use.

This product is supplied subject to the terms and conditions, including the limited license, located at www.biolegend.com/terms) ("Terms") and may be used only as provided in the Terms. Without limiting the foregoing, BioLegend products may not be used for any Commercial Purpose as defined in the Terms, resold in any form, used in manufacturing, or reverse engineered, sequenced, or otherwise studied or used to learn its design or composition without express written approval of BioLegend. Regardless of the information given in this document, user is solely responsible for determining any license requirements necessary for user's intended use and assumes all risk and liability arising from use of the product. BioLegend is not responsible for patent infringement or any other risks or liabilities whatsoever resulting from the use of its products.

BioLegend, the BioLegend logo, and all other trademarks are property of BioLegend, Inc. or their respective owners, and all rights are reserved.

8999 BioLegend Way, San Diego, CA 92121 www.biolegend.com
Toll-Free Phone: 1-877-Bio-Legend (246-5343) Phone: (858) 768-5800 Fax: (877) 455-9587

BioLegend
From Revvity

Version: 2 Revision Date: 10/14/2013

APC anti-mouse TNF-α Antibody

**Catalog# / Size**	506307 / 25 µg<br>506308 / 100 µg
**Clone**	MP6-XT22
**Regulatory Status**	RUO
**Other Names**	Tumor necrosis factor-α, Cachectin, Necrosin, Macrophage cytotoxic factor (MCF), Differentiation inducing factor (DIF), TNFSF-2, TNF-a, TNF-alpha
**Isotype**	Rat IgG1, κ
**Description**	TNF-α is secreted by macrophages, monocytes, neutrophils, T-cells, and NK-cells. Many transformed cell lines also secrete TNF-α. Monomeric mouse TNF-α is a 156 amino acid protein (N-glycosylated) with a reported molecular weight of 17.5 kD. TNF-α forms multimeric complexes; stable trimers are most common in solution. A 26 kD membrane form of TNF-α has also been described. TNF-α binding to surface receptors elicits a wide array of biologic activities including: cytolysis and cytostasis of many tumor cell lines *in vitro*, hemorrhagic necrosis of tumors *in vivo*, increased fibroblast proliferation, and enhanced chemotaxis and phagocytosis in neutrophils.

Product Details

**Verified Reactivity**	Mouse
**Antibody Type**	Monoclonal
**Host Species**	Rat
**Immunogen**	*E. coli*-expressed, recombinant mouse TNF-α
**Formulation**	Phosphate-buffered solution, pH 7.2, containing 0.09% sodium azide.
**Preparation**	The antibody was purified by affinity chromatography, and conjugated with APC under optimal conditions.
**Concentration**	0.2 mg/ml
**Storage & Handling**	The antibody solution should be stored undiluted between 2°C and 8°C, and protected from prolonged exposure to light. **Do not freeze.**
**Application**	ICFC - Quality tested
**Recommended Usage**	Each lot of this antibody is quality control tested by intracellular immunofluorescent staining with flow cytometric analysis. For flow cytometric staining, the suggested use of this reagent is ≤ 0.25 µg per 10^6 cells in 100 µl volume. It is recommended that the reagent be titrated for optimal performance for each application.
**Excitation Laser**	Red Laser (633 nm)
**Application Notes**	**ELISA Capture**: The purified MP6-XT22 antibody is useful as the capture antibody in a sandwich ELISA when used in conjunction with the biotinylated Poly5160 antibody (Cat. No. 516003) as the detection antibody and recombinant mouse TNF-α (Cat. No. 575209) as the standard.<br>**Flow Cytometry**[6,11,12]:The fluorochrome-labeled MP6-XT22 antibody is useful for intracellular immunofluorescent staining and flow cytometric analysis to identify TNF-a-producing cells within mixed cell populations.<br>**Neutralization**[1,5,10,16,17]:The MP6-XT22 antibody can neutralize the bioactivity of natural or recombinant TNF-α. The LEAF™ purified antibody (Endotoxin < 0.1 EU/µg, Azide-Free, 0.2 µm filtered) is recommended for neutralization of mouse TNF-α bioactivity *in vivo* and *in vitro*(Cat. No. 506310). For *in vivo* studies or highly sensitive assays, we recommend Ultra-LEAF™ purified antibody (Cat. No. 506332) with a lower endotoxin limit than standard LEAF™ purified antibodies (Endotoxin < 0.01 EU/µg).<br>**Additional reported applications (for the relevant formats) include**: Western blotting, immunohistochemical staining of paraformaldehyde-fixed, saponin-treated frozen tissue sections[7-9] *in vivo* detection[5], immunofluorescence, and immunocytochemistry.

Note: For testing mouse TNF-α in serum, plasma or supernatant, BioLegend's ELISA Max™ Sets (Cat. No. 430901) are specially developed and recommended.

Application References

(PubMed link indicates BioLegend citation)

1. Abrams J, *et al.* 1992. *Immunol. Rev.* 127:5. (Neut)
2. Abrams J, *et al.* 1995. *Curr. Prot. Immunol.* John Wiley and Sons, New York. Unit 6.20
3. Mo X, *et al.* 1995. *J. Virol.* 69:1288.
4. Sarawar S, *et al.* 1994. *J. Immunol.* 153:1246.
5. Via C, *et al.* 2001. *J. Immunol.* 167:6821. (Neut)
6. Infante-Duarte C, *et al.* 2000 *J. Immunol.* 165:6107. (FC)
7. Jacobs M, *et al.* 2000. *Immunology* 100:494. (IHC)
8. Marinova-Mutachieva L, *et al.* 1997. *Clin. Exp. Immunol.* 107:507. (IHC)
9. Williams RO, *et al.* 2000. *J. Immunol.* 165:7240. (IHC)
10. Scanga CA, *et al.* 1999. *Infect. Immun.* 67:4531. (Neut)
11. Akilov OE, *et al.* 2007. *J. Leukoc. Biol.* 2007;10.1189/jlb.0706439. (FC)
12. Lawson BR, *et al.* 2007. *J. Immunol.* 178:5366. (FC)

See More

Product Citations

1. Toubai T, *et al.* 2017. Blood Adv. 1.095138889. PubMed
2. Yu X, *et al.* 2020. Nat Commun. 11:1110. PubMed
3. Battistello E, *et al.* 2023. Mol Cell. 83:1216. PubMed
4. Yang QC, *et al.* 2023. iScience. 26:106916. PubMed
5. Tang M, *et al.* 2023. Sci Adv. 9:eade6624. PubMed
6. Ozga AJ, *et al.* 2022. Immunity. 55:82. PubMed
7. Yang Y, *et al.* 2022. FEBS J. 289:5279. PubMed
8. Iberg CA, *et al.* 2022. Cell Rep. 39:110657. PubMed
9. Jeong GU, *et al.* 2022. Microbiol Spectr. 10:e0109122. PubMed
10. Chae CS, *et al.* 2022. Cancer Discov. 12:1904. PubMed
11. Luo B, *et al.* 2022. Oxid Med Cell Longev. 2022:8965903. PubMed
12. Yang H, *et al.* 2023. Cell Death Differ. 30:560. PubMed

RRID

AB_315428 (BioLegend Cat. No. 506307)
AB_315429 (BioLegend Cat. No. 506308)

Antigen Details

Structure

TNF superfamily; dimer/trimer; 17.5-150 kD (Mammalian)

Bioactivity

Paracrine/endocrine mediator of inflammatory and immune functions; selectively cytotoxic for transformed cells; endothelial cell alterations; chemoattractant

Cell Sources

Activated monocytes, neutrophils, macrophages, T cells, B cells, NK cells, LAK cells

Cell Targets

Monocytes, neutrophils, macrophages, T cells, fibroblasts, endothelial cells, osteoclasts, adipocytes, astroglia, microglia

Receptors

TNFRSF1A (TNF-R1, CD120a, TNFR-p60 Type β, p55); TNFRSF1B (TNF-R2, CD120b, TNFR-p80 Type A, p75)

Cell Type

Tregs

Biology Area

Immunology, Innate Immunity

Molecular Family

Cytokines/Chemokines

Antigen References

1. Fitzgerald K, *et al.* Eds. 2001. The Cytokine FactsBook. Academic Press, San Diego.
2. Beutler B, *et al.* 1988. *Annu. Rev. Biochem.* 57:505.
3. Beutler B, *et al.* 1989. *Annu. Rev. Immunol.* 7:625.
4. Tracey K, *et al.* 1993. *Crit. Care Med.* 21:S415.

Regulation

Processed by TACE for secretion; upregulated by interferons, IL-2, GM-CSF, substance P, bradykinin, PAF, immune complexes, and cyclooxygenase; downregulated by IL-6, TGF-β, vitamin D3, prostaglandin E2, and PAF antagonists

Gene ID

21926

Related Protocols

- [Surface and Intracellular Cytokine Staining for Flow Cytometry - Video]
- [Intracellular Flow Cytometry Staining Protocol]

Other Formats

Brilliant Violet 421™ anti-mouse TNF-α, Ultra-LEAF™ Purified anti-mouse TNF-α, Brilliant Violet 605™ anti-mouse TNF-α, Brilliant Violet 650™ anti-mouse TNF-α, Purified anti-mouse TNF-α (Maxpar® Ready), Brilliant Violet 510™ anti-mouse TNF-α, FITC anti-mouse TNF-α, APC anti-mouse TNF-α, Biotin anti-mouse TNF-α, Spark NIR™ 685 anti-mouse TNF-α, Alexa Fluor® 700 anti-mouse TNF-α, Brilliant Violet 711™ anti-mouse TNF-α, PE anti-mouse TNF-α, Purified anti-mouse TNF-α, Alexa Fluor® 488 anti-mouse TNF-α, PerCP/Cyanine5.5 anti-mouse TNF-α, Alexa Fluor® 647 anti-mouse TNF-α, TotalSeq™-C1529 anti-mouse TNF-α Antibody, Brilliant Violet 750™ anti-mouse TNF-α, PE/Cyanine7 anti-mouse TNF-α, Pacific Blue™ anti-mouse TNF-α, GoInVivo™ Purified anti-mouse TNF-α, Brilliant Violet 785™ anti-mouse TNF-α, APC/Cyanine7 anti-mouse TNF-α, PE/Dazzle™ 594 anti-mouse TNF-α, TotalSeq™-B1529 anti-mouse TNF-α Antibody

Product Data

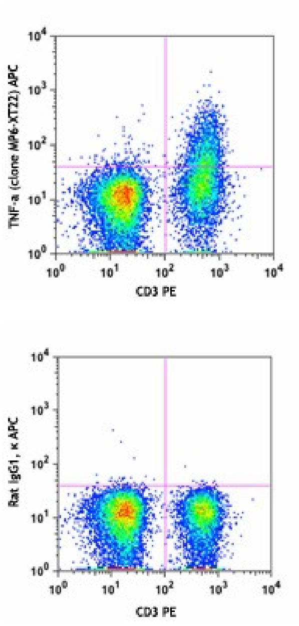

PMA + Ionomycin-stimulated C57BL/6 mouse splenocytes (in the presence of monensin) were stained with CD3 PE, fixed, permeabilized and then stained with TNF-α (clone MP6-XT22) APC (top) or rat IgG1, κ APC isotype control (bottom).

For Research Use Only. Not for diagnostic or therapeutic use.

This product is supplied subject to the terms and conditions, including the limited license, located at www.biolegend.com/terms) ("Terms") and may be used only as provided in the Terms. Without limiting the foregoing, BioLegend products may not be used for any Commercial Purpose as defined in the Terms, resold in any form, used in manufacturing, or reverse engineered, sequenced, or otherwise studied or used to learn its design or composition without express written approval of BioLegend. Regardless of the information given in this document, user is solely responsible for determining any license requirements necessary for user's intended use and assumes all risk and liability arising from use of the product. BioLegend is not responsible for patent infringement or any other risks or liabilities whatsoever resulting from the use of its products.

BioLegend, the BioLegend logo, and all other trademarks are property of BioLegend, Inc. or their respective owners, and all rights are reserved.

8999 BioLegend Way, San Diego, CA 92121 www.biolegend.com
Toll-Free Phone: 1-877-Bio-Legend (246-5343) Phone: (858) 768-5800 Fax: (877) 455-9587

PE/Cyanine7 anti-mouse IFN-γ Antibody

**Catalog# / Size**	505825 / 25 µg<br>505826 / 100 µg
**Clone**	XMG1.2
**Regulatory Status**	RUO
**Other Names**	Interferon-γ, Immune interferon, Type II interferon, T cell interferon, Macrophage-activating factor (MAF)
**Isotype**	Rat IgG1, κ
**Description**	IFN-γ is a potent multifunctional cytokine which is secreted primarily by activated NK cells and T cells. Originally characterized based on anti-viral activities, IFN-γ also exerts anti-proliferative, immunoregulatory, and proinflammatory activities. IFN-γ can upregulate MHC class I and II antigen expression by antigen-presenting cells.

Product Details

**Verified Reactivity**	Mouse
**Antibody Type**	Monoclonal
**Host Species**	Rat
**Immunogen**	*E. coli*-expressed, recombinant mouse IFN-γ
**Formulation**	Phosphate-buffered solution, pH 7.2, containing 0.09% sodium azide.
**Preparation**	The antibody was purified by affinity chromatography, and conjugated with PE/Cyanine7 under optimal conditions.
**Concentration**	0.2 mg/ml
**Storage & Handling**	The antibody solution should be stored undiluted between 2°C and 8°C, and protected from prolonged exposure to light. **Do not freeze.**
**Application**	ICFC - Quality tested
**Recommended Usage**	Each lot of this antibody is quality control tested by intracellular immunofluorescent staining with flow cytometric analysis. For flow cytometric staining, the suggested use of this reagent is ≤1.0 µg per million cells in 100 µl volume. It is recommended that the reagent be titrated for optimal performance for each application.
**Excitation Laser**	Blue Laser (488 nm)<br>Green Laser (532 nm)/Yellow-Green Laser (561 nm)
**Application Notes**	**ELISA[1-4,11,14] or ELISPOT[5] Detection:** The biotinylated XMG1.2 antibody is useful as a detection antibody for a sandwich ELISA or ELISPOT assay, when used in conjunction with purified R4-6A2 antibody (Cat. No. 505702/505706) as the capture antibody and recombinant mouse IFN-γ (Cat. No. 575309) as the standard.<br>**ELISA or ELISPOT Capture:** The purified XMG1.2 antibody is useful as a capture antibody for a sandwich ELISA or ELISPOT assay, when used in conjunction with biotinylated R4-6A2 antibody (Cat. No. 505704) as the detection antibody and recombinant mouse IFN-γ (Cat. No. 575309) as the standard. The LEAF™ purified antibody is suggested for ELISPOT capture (Cat. No. 505812).<br>**Flow Cytometry[7,8,12,13,16]:** The fluorochrome-labeled XMG1.2 antibody is useful for intracellular immunofluorescent staining and flow cytometric analysis to identify IFN-γ-producing cells within mixed cell populations.<br>**Neutralization[1-3,9,10]:** The XMG1.2 antibody can neutralize the bioactivity of natural or recombinant IFN-γ. The LEAF™ purified antibody (Endotoxin <0.1 EU/µg, Azide-Free, 0.2 µm filtered) is recommended for neutralization of mouse IFN-γ bioactivity *in vivo* and *in vitro* (Cat. No. 505812). For *in vivo* studies or highly sensitive assays, we recommend Ultra-LEAF™ purified

antibody (Cat. No. 505834) with a lower endotoxin limit than standard LEAF™ purified antibodies (Endotoxin <0.01 EU/µg).
Additional reported applications (for the relevant formats) include: Western blotting, immunohistochemical staining of frozen tissue sections[6,22,23], and immunocytochemistry.
Note: For testing mouse IFN-γ in serum, plasma or supernatant, BioLegend's ELISA Max™ Sets (Cat. No. 430801 to 430806) are specially developed and recommended.

Application References

(PubMed link indicates BioLegend citation)

1. Abrams J, *et al.* 1992. *Immunol. Rev.* 127:5. (ELISA, Neut)
2. Sander B, *et al.* 1993. *J. Immunol. Meth.* 166:201. (ELISA, Neut)
3. Abrams J, *et al.* 1995. *Curr. Prot. Immunol.* John Wiley and Sons, New York. Unit 6.20. (ELISA, Neut)
4. Yang X, *et al.* 1993. *J. Immunoassay* 14:129. (ELISA)
5. Klinman D, *et al.* 1994. *Curr. Prot. Immunol.* John Wiley and Sons, New York. Unit 6.19. (ELISPOT)
6. Sander B, *et al.* 1991. *Immunol. Rev.* 119:65. (IHC)
7. Ferrick D, *et al.* 1995. *Nature* 373:255. (FC)
8. Ko SY, *et al.* 2005. *J. Immunol.* 175:3309. (FC) PubMed
9. Peterson KE, *et al.* 2000. *J. Virol.* 74:5363. (Neut)
10. DeKrey GK, *et al.* 1998. *Infect. Immun.* 66:827. (Neut)
11. Dzhagalov I, *et al.* 2007. *J. Immunol.* 178:2113. (ELISA)
12. Lawson BR, *et al.* 2007. *J. Immunol.* 178:5366. (FC)

See More

Product Citations

1. Madireddi S, *et al.* 2014. J Exp Med. 211:1433. PubMed
2. Harsha Krovi S, *et al.* 2020. Nat Commun. 4.790277778. PubMed
3. Laura C Burzynski *et al.* 2019. Immunity. 50(4):1033-1042 . PubMed
4. Wang X, *et al.* 2019. Cell Res. 29:787. PubMed
5. Alghamri MS, *et al.* 2022. ACS Nano. 16:8729. PubMed
6. He Y, *et al.* 2022. Adv Healthc Mater. 11:e2200905. PubMed
7. Guo A, *et al.* 2022. Nature. 607:135. PubMed
8. Kedmi R, *et al.* 2022. Nature. 610:737. PubMed
9. Deák P, *et al.* 2022. Cell Rep. 41:111563. PubMed
10. Qiu C, *et al.* 2022. Front Bioeng Biotechnol. 10:1027619. PubMed
11. Wilson NG, *et al.* 2023. iScience. 26:105991. PubMed
12. Le DT, *et al.* 2023. iScience. 26:106059. PubMed

RRID

AB_1595591 (BioLegend Cat. No. 505825)
AB_2295770 (BioLegend Cat. No. 505826)

Antigen Details

Structure
Cytokine; dimer; 40-80 kD (Mammalian)

Bioactivity
Antiviral/antiparasitic activities; inhibits proliferation; enhances MHC class I and II expression on APCs

Cell Sources
$CD8^+$ and $CD4^+$ T cells, NK cells

Cell Targets
T cells, B cells, macrophages, NK cells, endothelial cells, fibroblasts

Receptors
IFN-γRα (CDw119) dimerized with IFN-γRβ (AF-1)

Cell Type
Tregs

Biology Area
Cell Biology, Immunology, Neuroinflammation, Neuroscience

Molecular Family
Cytokines/Chemokines

Antigen References

1. Fitzgerald K, *et al.* Eds. 2001. The Cytokine FactsBook. Academic Press, San Diego.
2. De Maeyer E, *et al.* 1992. *Curr. Opin. Immunol.* 4:321.
3. Farrar M, *et al.* 1993. *Annu. Rev. Immunol.* 11:571.
4. Gray P, *et al.* 1987. *Lymphokines* 13:151.

Regulation
Upregulated by IL-2, FGF-basic, EGF; downregulated by 1-α-25-Dihydroxy vitamin D3, dexamethasone

Gene ID
15978

Related Protocols

- [Surface and Intracellular Cytokine Staining for Flow Cytometry - Video]
- [Intracellular Flow Cytometry Staining Protocol]

Other Formats

Brilliant Violet 421™ anti-mouse IFN-γ, Brilliant Violet 711™ anti-mouse IFN-γ, Brilliant Violet 650™ anti-mouse IFN-γ, Brilliant Violet 605™ anti-mouse IFN-γ, Ultra-LEAF™ Purified anti-mouse IFN-γ, Brilliant Violet 785™ anti-mouse IFN-γ, Brilliant Violet 510™ anti-mouse IFN-γ, Purified anti-mouse IFN-γ (Maxpar® Ready), Purified anti-mouse IFN-γ, Spark NIR™ 685 anti-mouse IFN-γ, PE anti-mouse IFN-γ, Spark UV™ 387 anti-mouse IFN-γ, Brilliant Violet 750™ anti-mouse IFN-γ, Alexa Fluor® 700 anti-mouse IFN-γ, APC anti-mouse IFN-γ, Biotin anti-mouse IFN-γ, FITC anti-mouse IFN-γ, PE/Dazzle™ 594 anti-mouse IFN-γ, APC/Cyanine7 anti-mouse IFN-γ, TotalSeq™-C1526 anti-mouse IFN-γ Antibody, GoInVivo™ Purified anti-mouse IFN-γ, Pacific Blue™ anti-mouse IFN-γ, PerCP/Cyanine5.5 anti-mouse IFN-γ, PE/Cyanine7 anti-mouse IFN-γ, APC/Fire™ 750 anti-mouse IFN-γ, Alexa Fluor® 488 anti-mouse IFN-γ, Alexa Fluor® 647 anti-mouse IFN-γ, Spark PLUS UV395™ anti-mouse IFN-γ

Product Data

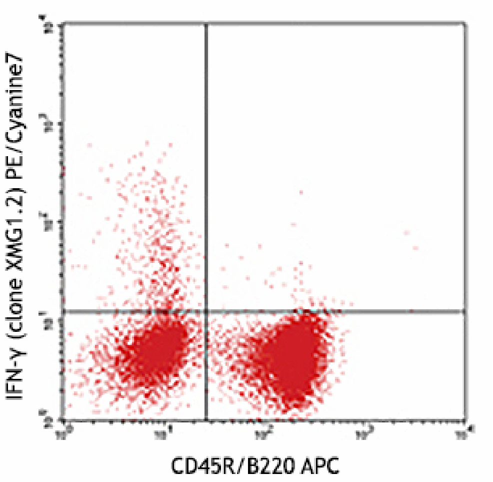

PMA/Ionomycin-stimulated (6hrs) C57BL/6 mouse splenocytes stained with B220 (RA3-6B2) APC and XMG1.2 PE/Cyanine7

For Research Use Only. Not for diagnostic or therapeutic use.

This product is supplied subject to the terms and conditions, including the limited license, located at www.biolegend.com/terms) ("Terms") and may be used only as provided in the Terms. Without limiting the foregoing, BioLegend products may not be used for any Commercial Purpose as defined in the Terms, resold in any form, used in manufacturing, or reverse engineered, sequenced, or otherwise studied or used to learn its design or composition without express written approval of BioLegend. Regardless of the information given in this document, user is solely responsible for determining any license requirements necessary for user's intended use and assumes all risk and liability arising from use of the product. BioLegend is not responsible for patent infringement or any other risks or liabilities whatsoever resulting from the use of its products.

BioLegend, the BioLegend logo, and all other trademarks are property of BioLegend, Inc. or their respective owners, and all rights are reserved.

8999 BioLegend Way, San Diego, CA 92121 www.biolegend.com
Toll-Free Phone: 1-877-Bio-Legend (246-5343) Phone: (858) 768-5800 Fax: (877) 455-9587

IFN-gamma Protein, Human

目录号:	HY-P7025
同用名:	rHuIFN-γ; IFNG; IFN-gamma; Interferon gamma
种属:	Human
表达系统:	E. coli
蛋白编号:	P01579 (Q24-Q166)
基因ID:	3458
分子量:	Approximately 16-18 kDa, based on SDS-PAGE under reducing conditions.

产品信息

氨基酸序列	Q D P Y V K E A E N L K K Y F N A G H S D V A D N G T L F L G I L K N W K E E S<br>D R K I M Q S Q I V S F Y F K L F K N F K D D Q S I Q K S V E T I K E D M N V K<br>F F N S N K K K R D D F E K L T N Y S V T D L N V Q R K A I H E L I Q V M A E L<br>S P A A K T G K R K R S Q M L F R G R R A S Q
生物活性	The ED_{50} is <1 ng/mL as measured by its ability to inhibit the proliferation of HT-29 cells., corresponding to a specific activity of >1 × 10^6 units/mg.
性状	Lyophilized powder
组分	1.Lyophilized from a 0.22 μm filtered solution of PBS.<br>2.Lyophilized from a 0.22 μm filtered solution of PBS, 5 % trehalose, 5% mannitol, pH 7.4.<br>3.Lyophilized from a 0.22 μm filtered solution of PBS, 5% trehalose, 5% mannitol, pH 7.4.<br>4.Lyophilized from a 0.22 μm filtered solution of PBS, pH 7.4, 8% trehalose.<br>Please refer to the lot-specific COA for specific buffer information.
内毒素含量	<1 EU/μg, determined by LAL method.
复溶方法	It is not recommended to reconstitute to a concentration less than 100 μg/mL in ddH_2O. For long term storage it is recommended to add a carrier protein (0.1% BSA, 5% HSA, 10% FBS or 5% Trehalose).
保存条件&期限	Stored at -20°C for 2 years from date of receipt. After reconstitution, it is stable at 4°C for 1 week or -20°C for longer (with carrier protein). It is recommended to freeze aliquots at -20°C or -80°C for extended storage.
运输条件	Room temperature in continental US; may vary elsewhere.

描述

研究背景	Human Interferon-gamma (hIFNγ) is naturally produced by $CD4^+$ T helper cell type 1 (Th1) lymphocytes, $CD8^+$ cytotoxic

lymphocytes, natural killer (NK) cells, B cells, NKT cells, and professional antigen-presenting cells (APCs). Secretion of hIFNγ by NK cells and APCs is important in early host reactions against infection while production of hIFNγ by T lymphocytes is important in the adaptive immune response. hIFNγ shows antiviral and antitumor activity and is involved in complex interactions of cellular metabolism and differentiation[1]. Interferon-gamma (IFN-γ) is a cytokine with potent immunomodulatory propertiy. IFN-γ activates cells via a different receptor than IFN-α and IFN-β, which accounts for the different physiological properties of the proteins[2].

REFERENCES

[1]. Jiang Z, et al., IFI16 directly senses viral RNA and enhances RIG-I transcription and activation to restrict influenza virus infection. Nat Microbiol. 2021 Jul;6(7):932-945.

[2]. Gao L, et al., MiR-873/PD-L1 axis regulates the stemness of breast cancer cells. EBioMedicine. 2019 Mar;41:395-407.

[3]. Razaghi A, et al. Review of the recombinant human interferon gamma as an immunotherapeutic: Impacts of production platforms and glycosylation. J Biotechnol. 2016 Dec 20;240:48-60.

[4]. Fam CM, et al. PEGylation improves the pharmacokinetic properties and ability of interferon gamma to inhibit growth of a human tumor xenograft in athymic mice. J Interferon Cytokine Res. 2014 Oct;34(10):759-68.

关注 MCE 中国 公众号，
获取前沿资讯、最新活动和
实用科研工具

MedChemExpress (MCE) 只为有资质的科研机构、医药企业基于科学研究或药证申报的用途提供医药研发服务，
不为任何个人或者非科研性质的、非用于药证申报使用等其他用途提供服务

Tel: 400-820-3792; 021-58955995　Fax: 021-53700325　E-mail: tech@MedChemExpress.cn
Master of Bioactive Molecules — 您身边的生物活性分子大师

购买产品将视为接受 MCE 的销售条款及条件

MCE® MedChemExpress

Product Data Sheet

抑制剂 · 化合物库 · 蛋白

M-CSF Protein, Human (223a.a, HEK293, His)

目录号:	HY-P70488
同用名:	Macrophage Colony-Stimulating Factor 1; CSF-1; M-CSF; MCSF; Lanimostim; CSF1
种属:	Human
表达系统:	HEK293
蛋白编号:	P09603-1 (E33-R255)
基因ID:	1435
分子量:	Approximately 34-50 kDa, based on SDS-PAGE under reducing conditions, due to the glycosylation.

产品信息

氨基酸序列	E E V S E Y C S H M I G S G H L Q S L Q R L I D S Q M E T S C Q I T F E F V D Q<br>E Q L K D P V C Y L K K A F L L V Q D I M E D T M R F R D N T P N A I A I V Q L<br>Q E L S L R L K S C F T K D Y E E H D K A C V R T F Y E T P L Q L L E K V K N V<br>F N E T K N L L D K D W N I F S K N C N N S F A E C S S Q D V V T K P D C N C L<br>Y P K A I P S S D P A S V S P H Q P L A P S M A P V A G L T W E D S E G T E G S<br>S L L P G E Q P L H T V D P G S A K Q R P P R
生物活性	Measured in a cell proliferation assay using M-NFS-60 mouse myelogenous leukemia lymphoblast cells. The ED_{50} for this effect is 2-10 ng/mL, corresponding to a specific activity is ≤6.69×10^5 units/mg.
性状	Lyophilized powder
组分	1.Lyophilized from a 0.22 μm filtered solution of 20 mM PB, 150 mM NaCl, pH 7.4.<br>2.Lyophilized from a 0.22 μm filtered solution of 20 mM PB, 150 mM NaCl, 4% Mannitol, pH 7.2.<br>Please refer to the lot-specific COA for specific buffer information.
内毒素含量	<0.01 EU/μg, determined by LAL method.
复溶方法	It is not recommended to reconstitute to a concentration less than 100 μg/mL in ddH_2O. For long term storage it is recommended to add a carrier protein (0.1% BSA, 5% HSA, 10% FBS or 5% Trehalose).
保存条件&期限	Stored at -20°C for 2 years from date of receipt. After reconstitution, it is stable at 4°C for 1 week or -20°C for longer (with carrier protein). It is recommended to freeze aliquots at -20°C or -80°C for extended storage.
运输条件	Room temperature in continental US; may vary elsewhere.

描述

研究背景	M-CSF Protein is a vital cytokine involved in regulating the survival, proliferation, and differentiation of hematopoietic

	precursor cells, particularly mononuclear phagocytes like macrophages and monocytes. It plays a crucial role in innate immunity and inflammatory processes by promoting the release of pro-inflammatory chemokines. Additionally, M-CSF Protein is essential for osteoclast proliferation and differentiation, regulating bone resorption, and normal bone development. It is also necessary for normal male and female fertility. Moreover, M-CSF Protein contributes to the reorganization of the actin cytoskeleton, facilitating membrane ruffle formation, cell adhesion, and cell migration. Furthermore, it plays a role in lipoprotein clearance. M-CSF Protein can exist in different forms, such as homodimer or heterodimer configurations, and it interacts with CSF1R.

关注 MCE 中国 公众号，
获取前沿资讯、最新活动和
实用科研工具

MedChemExpress (MCE) 只为有资质的科研机构、医药企业基于科学研究或药证申报的用途提供医药研发服务，
不为任何个人或者非科研性质的、非用于药证申报使用等其他用途提供服务

Tel: 400-820-3792; 021-58955995　　Fax: 021-53700325　　E-mail: tech@MedChemExpress.cn

Master of Bioactive Molecules — 您身边的生物活性分子大师

购买产品将视为接受 MCE 的销售条款及条件

Actin

Vendor: MilliporeSigma
Catalog #: MAB1501
View Antibody Link

Simple Western Antibody Datasheet

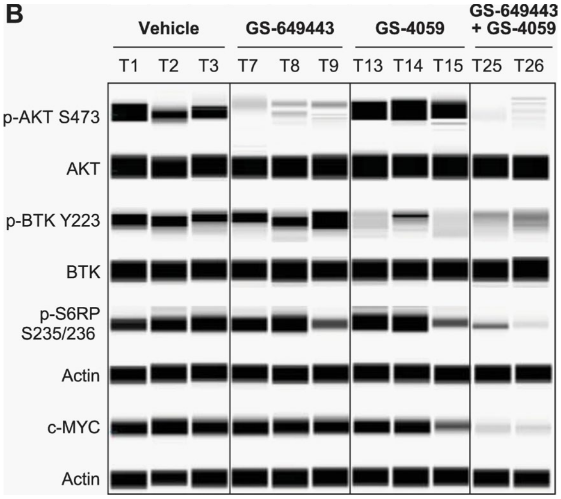

Figure 2B: In vivo inhibition of PI3Kδ and BTK leads to TMD8 tumor regression.(A) Response of randomized TMD8 xenograft tumors (n = 13 per group) treated with PI3Kδ inhibitor (GS-649443, 1 or 5 mg/kg, BID), BTK inhibitor (ONO/GS-4059, 3 or 10 mg/kg, BID) or combination. Tumor volumes are expressed as mean ± SEM with P<0.05, P <0.0001 as compared to vehicle animals. (B) Tumors from vehicle, GS-649443 (5 mg/kg), ONO/GS-4059 (10 mg/kg) or GS-649443 + ONO/GS-4059 (5 mg/kg + 10 mg/kg) were collected 2 hours post morning dose on day 3 of dosing, ground and lysed. Protein expression was analyzed using Simple Western; T = tumor. (C) Average of same tumors (from (B)) for each treatment group (n = 3 f... See reference below for more information.

Image collected and cropped by CiteAb from: PLoS One. 2017 Feb 8;12(2):e0171221.

Under copyright license: CC BY

Antibody	
Name	Anti-Actin Antibody, clone C4
Target Antigen	Actin
Reactant	Mus Musculus (House Mouse)
Antibody Type	Primary
Host	Mouse
Clonality	Monoclonal
Alternate Identifier	RRID: AB_2223041

Assay	
Sample Type	Spinal cord
Antibody Dilution	1:100
Separation Type	Size
Observed kDa	Not stated
Publications (6 found)	Clin Cancer Res. 2024 Aug 15;30(16):3622-3639. // J Clin Invest. 2023 Dec 7;134(4):e176311. // Biology (Basel). 2023 Jun 12;12(6):845. // Cell Death Dis. 2023 Mar 25;14(3):213. // J Physiol. 2017 Dec 1;595(23):7167-7183. // PLoS One. 2017 Feb 8;12(2):e0171221.

For additional information on this antibody view antibody link.

This antibody is cited for use with Simple Western™ technology. To learn about Simple Western technology, available antibodies, or to submit new antibodies, visit the links below. For additional information, please contact:

support@proteinsimple.com

Simple Western Systems   Simple Western Antibody Database   Submit Antibody Validation Data

For research use or manufacturing purposes only. Trademarks and registered trademarks are the property of their respective owners.

bio-techne // Global Developer, Manufacturer, and Supplier of High-Quality Reagents, Analytical Instruments, and Precision Diagnostics.

INCLUDES R&D Systems™ Novus Biologicals™ Tocris Bioscience™ ProteinSimple™ ACD™ ExosomeDx™ Asuragen® Lunaphore™

Revision 3

Store at -20C

#8635

IkappaB beta (7B4) Mouse Monoclonal Antibody

CST Logo

Orders: 877-616-CELL (2355)
orders@cellsignal.com

Support: 877-678-TECH (8324)

Web: info@cellsignal.com
cellsignal.com

3 Trask Lane | Danvers | Massachusetts | 01923 | USA

For Research Use Only. Not for Use in Diagnostic Procedures.

Applications:	Reactivity:	Sensitivity:	MW (kDa):	Source/Isotype:	UniProt ID:	Entrez-Gene Id:
W	H	Endogenous	48	Mouse IgG1	#Q15653	4793

Product Usage Information

Application	Dilution
Western Blotting	1:1000

Storage

Supplied in 10 mM sodium HEPES (pH 7.5), 150 mM NaCl, 100 µg/ml BSA, 50% glycerol and less than 0.02% sodium azide. Store at –20°C. Do not aliquot the antibody.

Specificity/Sensitivity

IkappaB beta (7B4) Mouse Monoclonal Antibody recognizes endogenous levels of total IκBβ protein.

Source / Purification

Monoclonal antibody is produced by immunizing animals with a recombinant protein specific to a carboxy terminal fragment of human IκBβ protein.

Background

The NF-κB/Rel transcription factors are present in the cytosol in an inactive state complexed with the inhibitory IκB proteins (1-3). Activation occurs via phosphorylation of IκBα at Ser32 and Ser36 followed by proteasome-mediated degradation that results in the release and nuclear translocation of active NF-κB (3-7). IκBα phosphorylation and resulting Rel-dependent transcription are activated by a highly diverse group of extracellular signals including inflammatory cytokines, growth factors, and chemokines. Kinases that phosphorylate IκB at these activating sites have been identified (8). The regulation of IκBβ and IκBε is similar to that of IκBα. However, the phosphorylation and ubiquitin-mediated degradation of these proteins occurs with much slower kinetics (9). IKK phosphorylation of IκBβ occurs at Ser19 and Ser23, while IκBε can be phosphorylated at Ser18 and Ser22 (10).

Background References

1. Baeuerle, P.A. and Baltimore, D. (1988) *Science* 242, 540-6.
2. Beg, A.A. and Baldwin, A.S. (1993) *Genes Dev* 7, 2064-70.
3. Finco, T.S. et al. (1994) *Proc Natl Acad Sci USA* 91, 11884-8.
4. Brown, K. et al. (1995) *Science* 267, 1485-8.
5. Brockman, J.A. et al. (1995) *Mol Cell Biol* 15, 2809-18.
6. Traenckner, E.B. et al. (1995) *EMBO J* 14, 2876-83.
7. Chen, Z.J. et al. (1996) *Cell* 84, 853-62.
8. Karin, M. and Ben-Neriah, Y. (2000) *Annu Rev Immunol* 18, 621-63.
9. Hoffmann, A. et al. (2002) *Science* 298, 1241-5.
10. Shirane, M. et al. (1999) *J Biol Chem* 274, 28169-74.

Species Reactivity

Species reactivity is determined by testing in at least one approved application (e.g., western blot).

Western Blot Buffer

IMPORTANT: For western blots, incubate membrane with diluted primary antibody in 5% w/v BSA, 1X TBS, 0.1% Tween® 20 at 4°C with gentle shaking, overnight.

Applications Key

W: Western Blotting

Cross-Reactivity Key

H: Human

Trademarks and Patents

Cell Signaling Technology is a trademark of Cell Signaling Technology, Inc.

All other trademarks are the property of their respective owners. Visit cellsignal.com/trademarks for more information.

限制使用

除非 CST 的合法授书代表以书面形式书行明确同意，否书以下条款适用于 CST、其关书方或分书商提供的书品。任何书充本条款或与本条款不同的客书条款和条件，除非书 CST 的合法授书代表以书面形式书独接受，否书均被拒书，并且无效。

专品专有“专供研究使用”的专专或专似的专专声明，且未专得美国食品和专品管理局或其他外国或国内专管机专专专任何用途的批准、准专或专可。客专不得将任何专品用于任何专断或治专目的，或以任何不符合专专声明的方式使用专品。CST 专售或专可的专品提供专作专最专用专的客专，且专用于研专用途。将专品用于专断、专防或治专目的，或专专售（专独或作专专成）或其他商专目的而专专专品，均需要 CST 的专独专可。客专：(a) 不得专独或与其他材料专合向任何第三方出售、专可、出借、捐专或以其他方式专专或提供任何专品，或使用专品制造任何商专专品，(b) 不得复制、修改、逆向工程、反专专、反专专专品或以其他方式专专专专专品的基专专专或技专，或使用专品开专任何与 CST 的专品或服专专争的专品或服

专， (c) 不得更改或专除专品上的任何商专、商品名称、徽专、专利或版专声明或专专，(d) 只能根据 CST 的专品专售条款和任何适用文档使用专品， (e) 专遵守客专与专品一起使用的任何第三方专品或服专的任何专可、服专条款或专似专专

Revision 3

#8635

IkappaB beta (7B4) Mouse Monoclonal Antibody

Cell Signaling Technology Logo

Western blot analysis of extracts from various cell lines using IκBβ (7B4) Mouse mAb.

Western Blotting Image 1: IkappaB beta (7B4) Mouse Monoclonal Antibody

Western blot analysis of extracts from MCF7 cells, untreated (-) or treated with Human Tumor Necrosis Factor-α (hTNF-α) (20 ng/ml, 4 hr; +), using IκBβ (7B4) Mouse mAb (upper) and α-Tubulin (11H10) Rabbit mAb #2125 (lower).

Western Blotting Image 2: IkappaB beta (7B4) Mouse Monoclonal Antibody

For Research Use Only

6*His, His-Tag Monoclonal antibody

Catalog Number:66005-1-Ig 1203 Publications

proteintech®
Antibodies | ELISA kits | Proteins
www.ptglab.com

Basic Information

Catalog Number:
66005-1-Ig

Source:
Mouse

Isotype:
IgG1

GenBank Accession Number:

GeneID (NCBI):

UNIPROT ID:
HISTAG

Full Name:

Calculated MW:
0.84 kDa

Purification Method:
Protein G purification

CloneNo.:
1B7G5

Recommended Dilutions:
WB: 1:5000-1:50000
IP: 0.5-4.0 ug for 1.0-3.0 mg of total protein lysate
IF/ICC: 1:200-1:800
FC: 0.20 ug per 10^6 cells in a 100 µl suspension

Applications

Tested Applications:
WB, IF/ICC, FC, IP, ELISA

Cited Applications:
WB, IHC, IF, FC, IP, CoIP, ChIP, ELISA

Species Specificity:
recombinant protein

Cited Species:
human, mouse, rat, chicken, hamster, yeast

Positive Controls:

WB : recombinant protein,

IP : Transfected HEK-293 cells,

IF/ICC : Transfected HEK-293 cells,

FC : Transfexted HEK-293 cells,

Background Information

Protein tags are protein or peptide sequences located either on the C- or N- terminal of the target protein, which facilitates one or several of the following characteristics: solubility, detection, purification, localization and expression. His-tag is often used for affinity purification and binding assays. Expressed His-tagged proteins can be purified and detected easily because the string of histidine residues binds to several types of immobilized metal ions, including nickel, cobalt and copper, under specific buffer conditions. The His-tag antibody is a useful tool for monitoring of the His-tagged proteins, and recognizes His-tags placed at N-terminal, C-terminal, and internal regions of fusion proteins expressed in bacteria, insect, and mammalian cells.

Notable Publications

Author	Pubmed ID	Journal	Application
Yanan Shao	36177860	Mol Plant Pathol	WB
Yueke Lin	36178239	EMBO Rep	WB,IP
Hao Yang	27708221	Oncotarget	

Storage

Storage:
Store at -20°C. Stable for one year after shipment.
Storage Buffer:
PBS with 0.02% sodium azide and 50% glycerol, pH7.3
Aliquoting is unnecessary for -20°C storage

Selected Validation Data

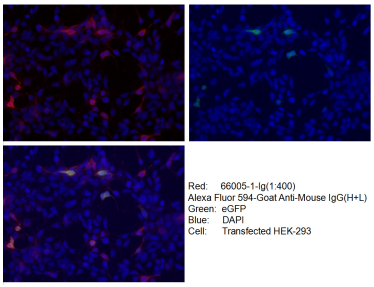

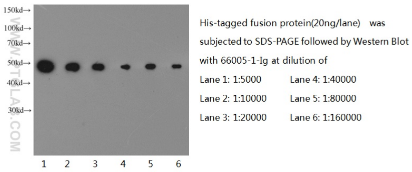

Western blot of 6*His-tagged fusion protein with anti-6*HIS tag (66005-1-Ig) at various dilutions.

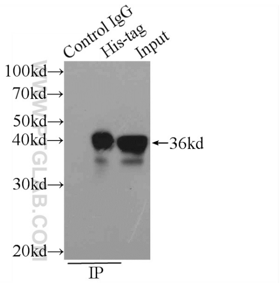

Immunofluorescent analysis of (-20°C Ethanol) fixed Transfected HEK-293 cells using 66005-1-Ig (6*His, His-Tag antibody) at dilution of 1:400 and Alexa Fluor 594-Conjugated AffiniPure Goat Anti-Mouse IgG(H+L).

IP result of anti-6*His, His-Tag (IP:66005-1-Ig, 7ug; Detection:66005-1-Ig 1:10000) with Transfected HEK-293 cells lysate 300ug.

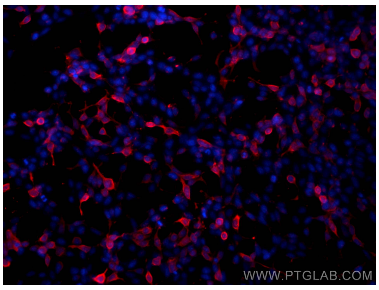

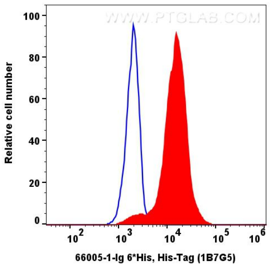

Immunofluorescent analysis of (-20°C Ethanol) fixed Transfected HEK-293 cells using 6*His, His-Tag antibody (66005-1-Ig, Clone: 1B7G5) at dilution of 1:800 and CoraLite®594-Conjugated AffiniPure Goat Anti-Mouse IgG(H+L) (SA00013-1).

1x10^6 His tagged FGFR2 Transfected HEK-293 cells were surface stained with 0.2 μg 6*His, His-Tag Monoclonal antibody (66005-1-Ig, Clone:1B7G5, red) and CoraLite488-conjugated Goat Anti-Mouse IgG(H+L) (Cat.NO. SA00013-1). Mouse IgG1 isotype control (66360-1-Ig, Clone: 1F8D3, blue) was parallel stained as control. Cells were not fixed.

For Research Use Only

Alpha Tubulin Monoclonal antibody

Catalog Number:66031-1-Ig Featured Product 1439 Publications

proteintech®
Antibodies | ELISA kits | Proteins
www.ptglab.com

Basic Information

Catalog Number:
66031-1-Ig

Source:
Mouse

Isotype:
IgG2b

Immunogen Catalog Number:
AG18034

GenBank Accession Number:
BC009314

GeneID (NCBI):
10376

UNIPROT ID:
P68363

Full Name:
tubulin, alpha 1b

Calculated MW:
50 kDa

Observed MW:
50-55 kDa

Purification Method:
Protein A purification

CloneNo.:
1E4C11

Recommended Dilutions:
WB: 1:20000-1:100000
IP: 0.5-4.0 ug for 1.0-3.0 mg of total protein lysate
IHC: 1:200-1:1000
IF/ICC: 1:500-1:2000
FC (Intra): 0.40 ug per 10^6 cells in a 100 μl suspension

Applications

Tested Applications:
WB, IHC, IF/ICC, FC (Intra), IP, ELISA

Cited Applications:
WB, IHC, IF, IP, CoIP

Species Specificity:
human, mouse, rat, canine

Cited Species:
human, mouse, rat, rabbit, monkey, chicken, zebrafish, sheep, goat, tick

Note-IHC: suggested antigen retrieval with TE buffer pH 9.0; (*) Alternatively, antigen retrieval may be performed with citrate buffer pH 6.0

Positive Controls:

WB : HeLa cells, HEK-293 cells, HepG2 cells, Jurkat cells, K-562 cells, HSC-T6 cells, NIH/3T3 cells, 4T1 cells

IP : HeLa cells,

IHC : human tonsillitis tissue, human liver cancer tissue, human colon cancer tissue

IF/ICC : HeLa cells, HepG2 cells, MCF-7 cells

FC (Intra) : HeLa cells,

Background Information

There are five tubulins in human cells: alpha, beta, gamma, delta, and epsilon. Tubulins are conserved across species. They form heterodimers, which multimerize to form a microtubule filament. An alpha and beta tubulin heterodimer is the basic structural unit of microtubules. The heterodimer does not come apart once formed. The alpha and beta tubulins, which are each about 55 kDa MW, are homologous but not identical. Alpha tubulin is useful for scientists across fields as an internal control due to its high, ubiquitous expression pattern. Tubulin expression may vary according to resistance to antimicrobial and antimitotic drugs. This antibody specifically recognizes Tubulin Alpha.

Notable Publications

Author	Pubmed ID	Journal	Application
Sirwan Sleman	36179070	Viral Immunol	WB
Pengcheng Ma	36179027	Sci Adv	WB
Wei Wen	33133097	Front Immunol	WB

Storage

Storage:
Store at -20°C. Stable for one year after shipment.
Storage Buffer:
PBS with 0.02% sodium azide and 50% glycerol, pH7.3
Aliquoting is unnecessary for -20°C storage

Selected Validation Data

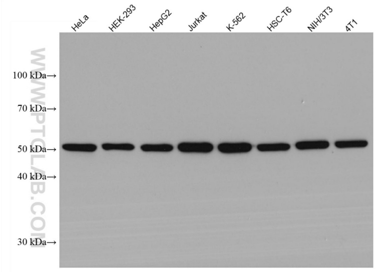

Various lysates were subjected to SDS PAGE followed by western blot with 66031-1-Ig (Alpha Tubulin antibody) at dilution of 1:200000 incubated at room temperature for 1.5 hours.

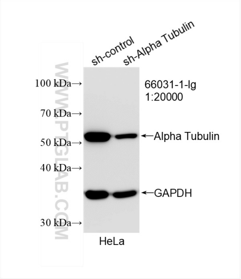

WB result of Alpha Tubulin antibody (66031-1-Ig; 1:20000; incubated at room temperature for 1.5 hours) with sh-Control and sh-Alpha Tubulin transfected HeLa cells.

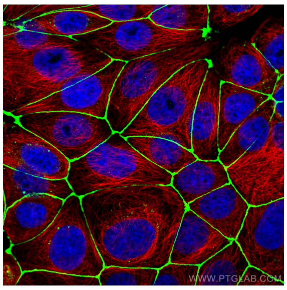

Immunofluorescent analysis of (4% PFA) fixed MCF-7 cells using Alpha Tubulin antibody (66031-1-Ig, Clone: 1E4C11) at dilution of 1:1000 and CoraLite®594-Conjugated AffiniPure Goat Anti-Rabbit IgG(H+L), ZO-1 antibody (21773-1-AP, green).

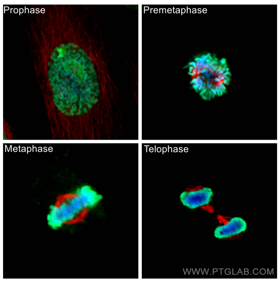

Immunofluorescent analysis of (4% PFA) fixed HeLa cells using Alpha Tubulin antibody (66031-1-Ig, Clone: 1E4C11) at dilution of 1:1000 and CoraLite®594-Conjugated AffiniPure Goat Anti-Mouse IgG(H+L), Histone H1.2 antibody (19649-1-AP, green).

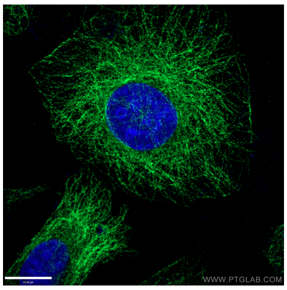

Immunofluorescent analysis of (-20℃ Ethanol) fixed HepG2 cells using 66031-1-Ig(alpha Tubulin antibody) at dilution of 1:100 and Alexa Fluor 488-conjugated AffiniPure Goat Anti-Mouse IgG(H+L).

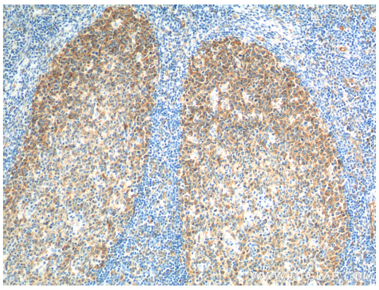

Immunohistochemical analysis of paraffin-embedded human tonsillitis tissue slide using 66031-1-Ig (alpha Tubulin antibody at dilution of 1:200 (under 10x lens). Heat mediated antigen retrieval with Tris-EDTA buffer (pH 9.0).

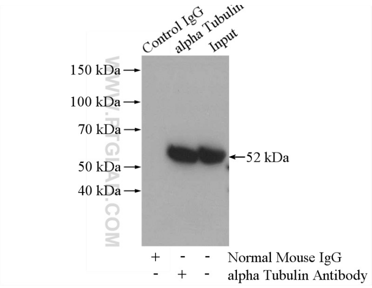

IP result of anti-Alpha Tubulin (IP:66031-1-Ig, 5ug; Detection:11224-1-AP 1:1000) with HeLa cells lysate 2800ug.

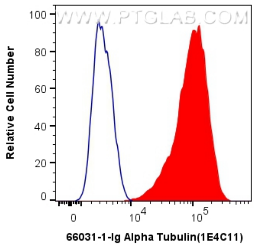

1X10^6 HeLa cells were intracellularly stained with 0.4 ug Anti-Human Alpha Tubulin (66031-1-Ig, Clone:1E4C11) and CoraLite®488-Conjugated AffiniPure Goat Anti-Mouse IgG(H+L) at dilution 1:1000 (red), or 0.4 ug Mouse IgG2b Isotype Control (MPC-11) (65128-1-Ig, Clone: MPC-11) (blue). Cells were fixed with 4% PFA and permeabilized with Flow Cytometry Perm Buffer.

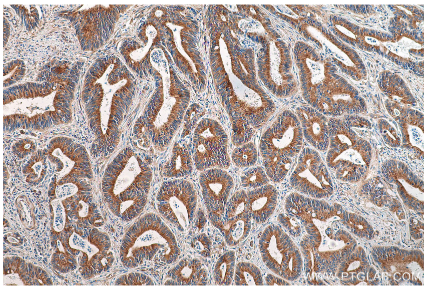

Immunohistochemical analysis of paraffin-embedded human colon cancer tissue slide using 66031-1-Ig (Alpha Tubulin antibody) at dilution of 1:1000 (under 10x lens). Heat mediated antigen retrieval with Tris-EDTA buffer (pH 9.0).

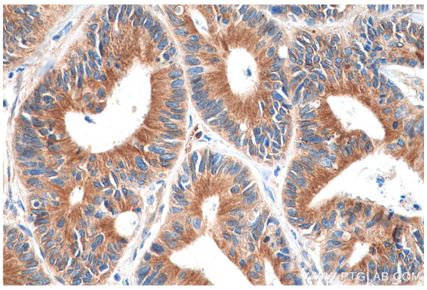

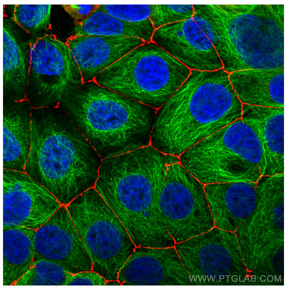

Immunohistochemical analysis of paraffin-embedded human colon cancer tissue slide using 66031-1-Ig (Alpha Tubulin antibody) at dilution of 1:1000 (under 40x lens). Heat mediated antigen retrieval with Tris-EDTA buffer (pH 9.0).

Immunofluorescent analysis of (4% PFA) fixed MCF-7 cells using Alpha Tubulin antibody (66031-1-Ig, Clone: 1E4C11) at dilution of 1:1000 and CoraLite®488-Conjugated AffiniPure Goat Anti-Mouse IgG(H+L), ZO-1 antibody (21773-1-AP, red).

Kinlogix

产品信息

J774A.1

小鼠腹水单核细胞瘤细胞

[Kinlogix cat: KC9041]

- **形态特征**：单核细胞样
- **生长特征**：贴壁
- **种属**： 小鼠源
- **组织来源**：腹水
- **疾病**：网状细胞肉瘤

规格及存储

◆ **常规出库**：

T25 培养瓶，1x10^{6}cells 活细胞

请及时放置于细胞培养箱(37℃,5%CO_2)

◆ **冻存株出库**：

同批次 2 管，1x10^{6}cells/管

请及时存储于液氮（-196℃）

使用范围

本产品仅限于科学研究。

培养条件

DMEM,high glucose+10%FBS

推荐培养体系：

- **基础培养基**：

DMEM,high glucose(Kinlogix cat:M1001,或 Gibco cat:11995,或同配方)

- **血清**：南美胎牛血清(Kinlogix cat:M301)

- **添加剂**：\

配套完全培养基（Kinlogix Cat: KC9041M）

传代方法：1:3 传代（培养面积比）
传代方式：该细胞不能用胰酶消化，用细胞刮刀或细胞铲进行处理。
换液频率：2~3 天换液 1 次
倍增时间：\
冻存液配方：DMEM,high glucose+10%FBS+10%DMSO
难度等级：++
培养要点：需用刮刀传代

特征特性：

该细胞来源于 BABL/cN 小鼠，有抗体依赖的吞噬作用，生长受硫酸葡聚糖、PPD 和 LPS 的抑制；可产生 IL-1β 和大量的溶菌酶。

广州科珞捷生物技术有限公司
Guangzhou Kinlogix Biotech Co.,Ltd.

Kinlogix

产品信息

RAW264.7

小鼠单核巨噬细胞

[Kinlogix cat: KC9001]

- **形态特征：**淋巴细胞样
- **生长特征：**贴壁
- **种属：** 小鼠源
- **组织来源：**腹水
- **疾病：**白血病

规格及存储

◆ **常规出库：**

T25 培养瓶，1x10^{6}cells 活细胞

请及时放置于细胞培养箱(37℃,5%CO_2)

◆ **冻存株出库：**

同批次 2 管，1x10^{6}cells/管

请及时存储于液氮（-196℃）

使用范围

本产品仅限于科学研究。

培养条件

DMEM,high glucose+10%FBS

推荐培养体系：

- **基础培养基：**

DMEM,high glucose(Kinlogix cat:M1001,或 Gibco cat:11995,或同配方)

- **血清：**南美胎牛血清(Kinlogix cat:M301)

- **添加剂：**\

配套完全培养基（Kinlogix Cat: KC9001M）

传代方法：1:3 传代（培养面积比）
传代方式：该细胞不能用胰酶消化，用细胞刮刀或细胞铲进行处理。
换液频率：每周换液 2-3 次
倍增时间：11 hours (from cell counting), 12 hours (from absorbance) (DOI=10.5897/IJBMBR2013.0154); ~30 hours (CLS).
冻存液配方：DMEM,high glucose+10%FBS+10%DMSO
难度等级：++
培养要点：需要用刮刀传代，建议高密度培养。细胞刮刀(Kinlogix cat:M2012)

特征特性：

此细胞株源自 BALB/c 小鼠由 Abelson 鼠科白血病病毒诱导的肿瘤。可产生溶菌酶；sIg-, Ia-，Thy-1.2-。为检测到病毒颗粒的分泌，XC 斑点形成试验阴性。该细胞可以胞饮中性红并吞噬乳胶颗粒与酵母聚糖；可以经抗体依赖分裂绵羊红细胞与肿瘤靶细胞；LPS 或 PPD 处理 2 天可诱导分裂红细胞，但对肿瘤靶细胞无作用。

广州科珞捷生物技术有限公司
Guangzhou Kinlogix Biotech Co.,Ltd.

Kinlogix

产品信息

HEK-293

人胚肾细胞

[Kinlogix cat: KC4002]

- **形态特征：**上皮细胞样
- **生长特征：**贴壁
- **种属：** 人源
- **组织来源：**肾
- **疾病：**转化细胞

规格及存储

◆ **常规出库：**

T25 培养瓶，$1x10^6$cells 活细胞

请及时放置于细胞培养箱(37℃,5%CO_2)

◆ **冻存株出库：**

同批次 2 管，$1x10^6$cells/管

请及时存储于液氮（-196℃）

使用范围

本产品仅限于科学研究。

培养条件

DMEM,high glucose+10%FBS

推荐培养体系：

- **基础培养基：**

DMEM,high glucose(Kinlogix cat:M1001,或 Gibco cat:11995,或同配方)

- **血清：**南美胎牛血清(Kinlogix cat:M301)

- **添加剂：**\

配套完全培养基（Kinlogix Cat: KC4002M）

传代方法：1:3-1:5 传代（培养面积比）

传代方式：消化 30 秒

换液频率：2~3 天换液 1 次

倍增时间：~30 hours (CLS); ~24-30 hours (DSMZ)

冻存液配方：DMEM,high glucose+10%FBS+10%DMSO

难度等级：++

培养要点：细胞贴壁性较差，容易飘起来，37℃静置可重新贴壁

特征特性：

早期报道中指出该细胞基因组中含有腺病毒 5（Ad5）基因组的左侧端和右侧端的 DNA，但是现在明确了只存在其左侧端的 DNA。经过对 Ad5 的插入点的克隆测序发现，Ad5 的 1～4344 位线性核苷酸整合入细胞染色体 19q13.2。该细胞为人类腺病毒载体扩增的宿主。可表达异常的玻连蛋白的细胞表面受体，由整合素 β1 亚单位和玻连蛋白受体 α-v 亚单位组成。生物安全级别为 2 级。

广州科珞捷生物技术有限公司
Guangzhou Kinlogix Biotech Co.,Ltd.
